# Allostery Wiring Map for Kinesin Energy Transduction and Its Evolution*[Fn FN2]

**DOI:** 10.1074/jbc.M116.733675

**Published:** 2016-08-08

**Authors:** Jessica Richard, Elizabeth D. Kim, Hoang Nguyen, Catherine D. Kim, Sunyoung Kim

**Affiliations:** From the Department of Biochemistry and Molecular Biology, Louisiana State University School of Medicine & Health Sciences Center, New Orleans, Louisiana 70112

**Keywords:** enzyme mechanism, kinesin, mechanotransduction, molecular motor, mutagenesis, ATP hydrolysis, double-mutant cycle analysis, long-range thermodynamic coupling, residue co-evolution, statistical coupling analysis

## Abstract

How signals between the kinesin active and cytoskeletal binding sites are transmitted is an open question and an allosteric question. By extracting correlated evolutionary changes within 700+ sequences, we built a model of residues that are energetically coupled and that define molecular routes for signal transmission. Typically, these coupled residues are located at multiple distal sites and thus are predicted to form a complex, non-linear network that wires together different functional sites in the protein. Of note, our model connected the site for ATP hydrolysis with sites that ultimately utilize its free energy, such as the microtubule-binding site, drug-binding loop 5, and necklinker. To confirm the calculated energetic connectivity between non-adjacent residues, double-mutant cycle analysis was conducted with 22 kinesin mutants. There was a direct correlation between thermodynamic coupling in experiment and evolutionarily derived energetic coupling. We conclude that energy transduction is coordinated by multiple distal sites in the protein rather than only being relayed through adjacent residues. Moreover, this allosteric map forecasts how energetic orchestration gives rise to different nanomotor behaviors within the superfamily.

## Introduction

Biological motors function by converting the chemical energy of ATP hydrolysis into mechanical work in the cell. Thus, molecular motors are free energy transducers, *i.e.* free energy from the active site is redistributed through the motor protein and ultimately produces a new protein conformational state. Diverse microtubule (MT)-based^2^ functions arise in part from differences in their mechanotransduction cycle. For example, members of certain kinesin families are capable of transporting cargo, whereas others modify the MT track (reviewed in Ref. 1).

**FIGURE 1.**
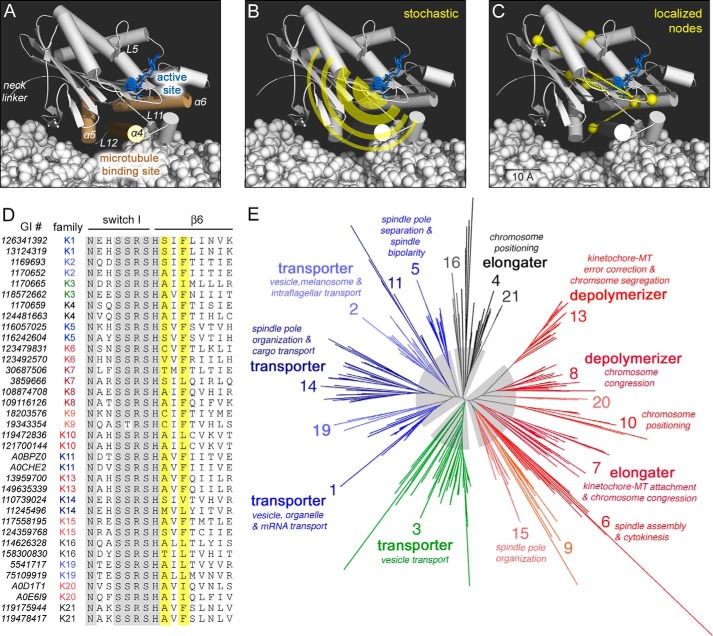
**Kinesin structure, function, and sequence are linked.** *A*, structural motifs identified within a kinesin motor domain (Protein Data Bank code 4HNA). *B* and *C*, two models of free energy dissipation in motors are stochastic dissipation (*B*) or routed to a discrete set of residues within the motor domain (*C*). A 10 Å scale bar is shown to estimate relative distances. *D*, SATé sequence alignment of switch I loop and β6 strand. Sequence representatives from each kinesin family (designated as families 1–11, 13–16, and 19–21) are included and annotated with the NCBI GI number. Strictly conserved positions are in *gray*, and SCA positions are in *yellow. E*, SATé kinesin phylogeny in radial form. Provided are labels for kinesin family number, kinesin-MT interaction, and ascribed cellular roles, if known. Clades are highlighted in *gray*; protein members and branches are *blue* and *green* for kinesin transporter clades and *red* and *black* for kinesin MT-modifier clades.

Our goal here is identification of key residues that choreograph transduction between the active site and the microtubule-binding site (see Fig. 1*A*). It is anticipated that this set of residues couples components that catalyze the free energy-donating reaction with the free energy-accepting ones that result in directed motion. Furthermore, this wiring network should be shared among kinesins and predict adjustment of mechanotransduction between motor families. Such knowledge would reveal mutations that can be used to systematically tune motor protein function.

By definition, mechanotransduction is one form of allostery, given that its quintessential property is long range communication. Long range effects in kinesin have been reported. In the first type of study, allosteric mechanisms are inferred from comparisons of well populated conformational states (2, 3) and are primarily descriptive. In the second, molecular dynamics calculations describe dynamic properties of motor proteins as thermally stochastic and yet asymmetric (see Fig. 1*B*; reviewed in Refs. 4–6). Theoretical treatments of allostery in other systems also show promise in uncovering fundamental principles of energy conversion, such as the idea that energy storage and transmission can occur in waves in secondary structures (reviewed in Refs. 7 and 8). Recent models define allostery as a thermodynamic phenomenon (9) and suggest that localized protein nodes can harvest and concentrate most of the energy at a few sites (Fig. 1*C*). This, in turn, would minimize energy dissipation (10–13).

However, the above efforts do not provide biochemical evidence of coupling, nor do they map a cascade of local induced-fit events that sequentially propagate over a long distance, *i.e.* a molecular wire (14, 15). To bridge this information gap, residue co-evolution has emerged as an important principle in the study of allostery. Statistical coupling analysis (SCA) identifies allosteric pathways in a polypeptide chain (16, 17). By monitoring amino acid distributions across a multiple sequence alignment, SCA identifies compensatory mutations that occurred during the course of evolution within a given protein family. Double-mutant cycle analysis showed that experimentally measured ΔΔ*G*_binding_ for one PDZ ligand-binding site correlated with SCA-derived ΔΔ*G*_stat_ values. These data led to the conclusion that (i) specific, distal residues in the protein were thermodynamically and energetically linked and (ii) SCA could uncover a network of linked residues that mediate an allosteric response.

Here, we use SCA to map a residue network for energy transduction that evolves across kinesin isoforms. We tested whether such identified residues, which are 7–30 Å apart in the motor domain, were thermodynamically coupled. Our mathematical and experimental results provide new information about the interrelation of energy from ATP hydrolysis to allosteric changes in this motor.

## Results

### 

#### 

##### We Lowered the Noise Typically Associated with Residue Co-evolution Analysis by Rigorous Pruning of Kinesin Sequences

A limiting factor in correlated mutation analysis, such as SCA, is a low signal-to-noise ratio (18). The data input for SCA is a multiple sequence alignment (MSA), and statistical correlations resulting from the analysis are dependent on the quality of this MSA. Low signal may arise due to insufficient data. High noise can arise from relatedness between sequences and errors in the sequence databases themselves.

To address the above points, the sequence dataset first must be large and varied to adequately reflect the evolutionary divergence of the protein family (16). Database searches readily yielded a large volume of kinesin sequences. At the initiation of this study, >3000 sequences were reported. However, 40% of sequences in public databases have either sequencing or annotation errors (19–21). By their own record, no SCA study (supplemental Table S1) described filtering of sequence entries for errors; only removal of sequences with particular forms of divergence (indels, or insertions and deletions) is documented.

We manually curated a kinesin sequence dataset that was checked for duplications, fragmented sequences, or sequences without a reference. Afterward, all redundant sequences, *i.e.* ≥95% sequence identity, were removed. The final dataset (supplemental Table S2) contained 726 motor domain sequences from all known kinesin families (22–25), 78 taxa, and all superkingdoms. This edited dataset has a greater number of sequences than found in most other residue co-evolution studies (supplemental Table S1).

##### For the Motor Field, Inaugural Usage of the SATé Algorithm Improved Bioinformatic Organization of the Kinesin Superfamily

The curated dataset was used as input for SATé, a maximum likelihood co-estimating algorithm (26) that performs MSA and phylogeny calculations in tandem. This approach evades errors in the starting alignment by constantly breaking and reorganizing both. The algorithm outperforms traditional two-phase methodologies (26–29). SATé produced a well resolved MSA (supplemental Files S1 and S2) and phylogeny (supplemental Files S3 and S4) for kinesin motor domain sequences.

The reliable sequence alignment is necessary to compare sequence changes across kinesin families and determine statistical relationships. SATé was successful in this respect. A sample of the MSA is provided in Fig. 1*D*. The switch I active site motif shown is strictly conserved (*gray highlight*) and contains only two variable positions (N*XX*SSR); most alignment methodologies are capable of resolving such conserved motifs. Adjacent to switch I in linear sequence is the β6 strand, which has greater sequence variability. Only the first N-terminal residue of β6 (His) is conserved across kinesin families. In comparison, the commonly used Clustal is known to produce poor alignments and gaps in analysis of ≥100 sequences (supplemental Fig. S1). Thus, we conclude that the above steps likely will improve the signal-to-noise ratio in residue co-evolution analysis.

In addition, the SATé algorithm increased the evolutionary resolution of kinesin clades in the phylogenetic tree (Fig. 1*E* and supplemental Files S3 and S4). Tree branches (Fig. 1*E*) are annotated with family number, mechanotransduction outcome, and cellular function. SATé predicted 18 kinesin families: the original 13 (22–25), 3 additions in Ref. 30, a new family (Kinesin-20) from the chromista and protozoa superkingdoms, and a novel orphan group (pseudoKinesin-21), currently unique to fungal taxa. These expansions to the kinesin superfamily are due to the increased taxonomic diversity included in our dataset.

Family member assignment in this SATé tree is consistent with prior phylogenetic models (22–25). Slight differences in family member composition are attributed to our unrooted calculations *versus* prior rooted assumptions. For example, two kinesins differ in family assignment from prior analyses: *Saccharomyces cerevisiae* Smy1 and *Drosophila melanogaster* Nod. ScSmy1 has been used as a divergent root in some prior kinesin phylogenies (24, 25), but not others. In our work, which incorporated extensive species and kingdom diversity, ScSmy1 is a kinesin-1, as in Ref. 22. DmNod is a second example of a kinesin that has inconsistent assignment between phylogenetic reports; it is a kinesin-4 here.

In the SATé tree (Fig. 1*E*), we observe novel correlations between kinesin-MT interaction and clade organization. Our dendogram has four distinct deep nodes, or clades, in the tree structure (Fig. 1*E*, *gray pie highlights*). Kinesins capable of processive motion and transport of cellular cargo reside in two clades (Fig. 1*E*, *blue* and *green*); these clades include kinesins that translocate to the plus ends of MTs (kinesin-1, -2, -3, -5, and -11) and to the MT minus ends (kinesin-14). Motors that modify MT tracks by elongation (kinesin-7), ambiguous molecular means (kinesin-4), or depolymerization (kinesin-13 and -8) occupy the remaining two clades (Fig. 1*E*, *black* and *red*).

To our knowledge, this segregation of processive transport motors in distinct clades from motors that modify MT-track dynamics is unique. Prior reports had either poor clade resolution or mixed kinesin families with disparate functions in a clade. We emphasize that our kinesin tree is an outcome of both data and algorithmic improvements. Long sought, robust association between sequence and function now is achievable with current methods.

##### Allosteric Networks for Kinesin Energy Transduction, Microtubule Binding, and Adenine Association Are Defined and Are Interconnected

**FIGURE 2.**
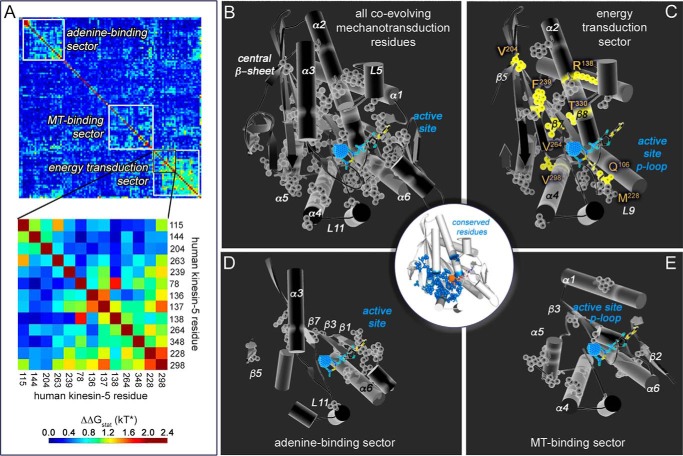
**Our allostery wiring map consists of a network of 65 co-evolving residues in the kinesin motor domain.** *A*, SCA output is a heat map matrix with residues clustered according to the degree of coupling between residue positions shown on the *x* and *y* axes. Heat map legend denotes the corresponding statistical coupling energies (kT*). Co-evolution was detected for residues within a range of 36–86% identity. The *inset* is a zoomed view of the indicated region with human Eg5 kinesin sequence residue labels. *B*, structural view of kinesin SCA residue side chains. SCA residues are shown as *spheres* on a kinesin motor domain structure (Protein Data Bank code 3HQD). Secondary structure motifs are labeled in *white. C–E*, predicted energy transduction sector (*C*), adenine-binding sector (*D*), and MT-binding sector residues (*E*) are shown as *spheres* on a clipped motor domain structure. Positions that co-evolve with the active site SCA residue Gln^106^ are shown in *yellow*. Kinesin residues that are conserved (*blue*, >90% identity) are shown in *circular inset* with a *white background*.

Using the above SATé MSA as the input, SCA detected 65 intercorrelated kinesin residues, which is equivalent to 18% of the motor domain residues (ΔΔ*G*_stat_ = 1.0–2.4 kT* in Fig. 2*A*, Table 1, and supplemental Table S2). Hereafter, they are referred to as “SCA residues.” This result was consistent with the observation that only a subset of residues (10–30%) co-evolve within a polypeptide chain (31). We note that SCA residues identified are not the same as residues that are conserved (Fig. 2, *white center circle*).

**TABLE 1:**
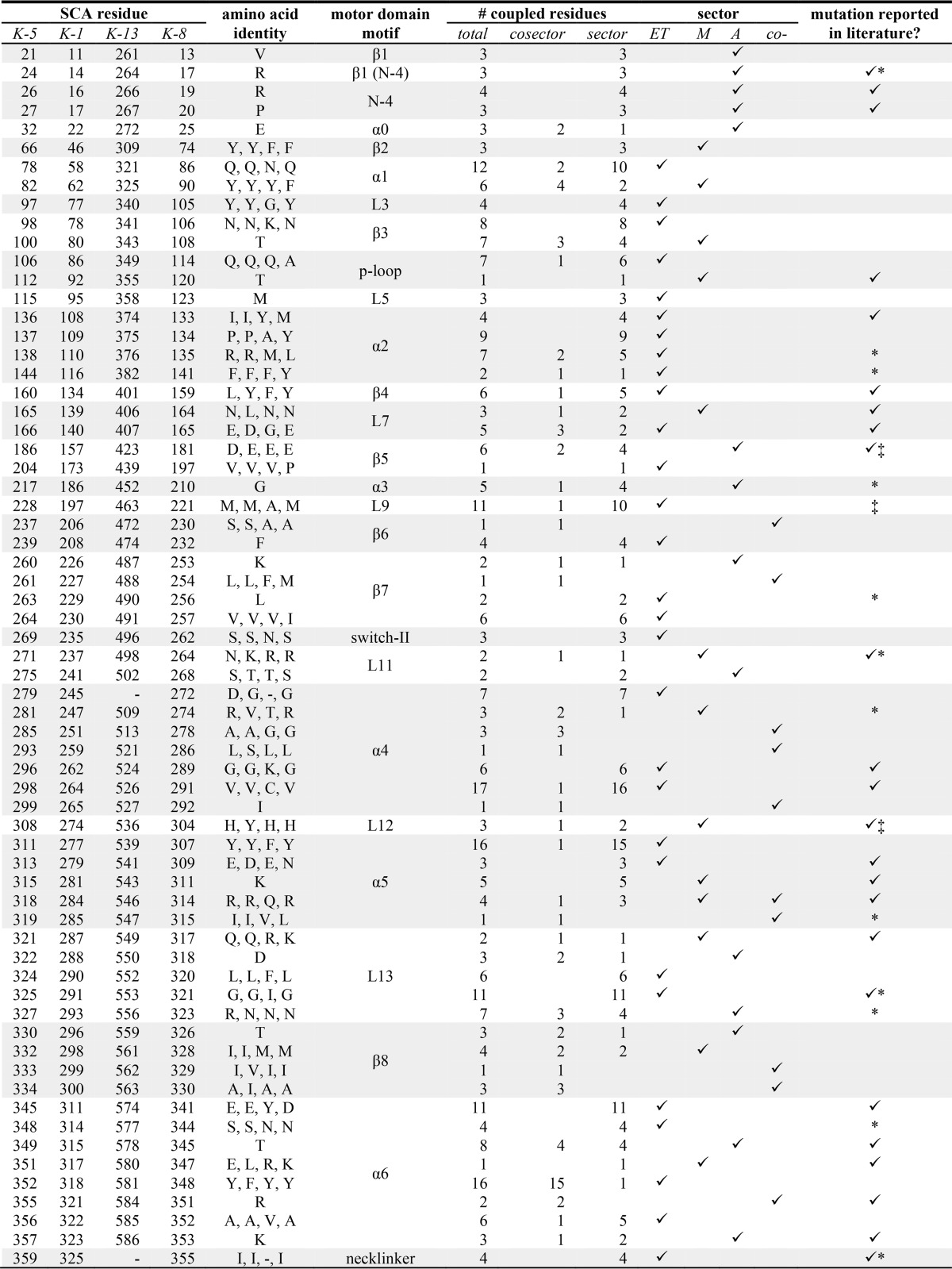
**Kinesin residues in the SCA network** The residues listed are from human kinesins: Eg5 (*K-5*, GI 116242604), Kif5b (*K-1*; GI 417216), Kif2C (*K-13*; GI 20141607), and Kif18A (*K-8*; GI 66774137), *i.e.* Tyr^46^ is in Kif5b, and Phe^309^ is in Kif2C at the homologous coordinate β2 strand position. The number of correlations and subdivision into residues within a single sector and those between sectors (co-sector) are listed. Energy transduction (ET), microtubule-binding (M), and adenine-binding (A) sectors are labeled. Positions mutated in *in vitro* studies are indicated by ✓, identified polymorphisms are indicated by *, and/or those associated with disease are indicated by ‡. - indicates a gap in the sequence alignment.

The clustered output matrix in heatmap form showed that the majority of kinesin residues did not co-evolve (ΔΔG_stat_ ≤ 0.6 kT*; Fig. 2*A*). A sample of residue correlations, using human Eg5 (kinesin-5) residue numbering, is shown in Fig. 2*A* (*inset*). Mutation of Eg5 residue 298 is statistically coupled to residues 228, 348, 264, and 137 (ΔΔG_stat_ ≥ 1 kT*). On the other hand, mutation of residue 298 is not statistically coupled to residues 144 and 204 (ΔΔG_stat_ < 1 kT*).

SCA residues occupy three distinct regions in the clustered output matrix (Fig. 2*A*, *white boxes*, and supplemental Fig. S2), termed protein sectors (32). When shown on a crystal structure, SCA residues are found to form a contiguous surface within the kinesin motor domain (Fig. 2*B*). We forecast that one sector is responsible for energy transduction (Fig. 2*C*). Two other sectors may have roles in binding the adenine moiety of ATP/ADP (Fig. 2*D* and Ref. 33) and the MT track (Fig. 2*E*). Kinesin protein sectors are quasi-independent (32). Co-sector residues link the individual sectors (34) and are conduits for allosteric cross-talk between sectors. Thus, our map contains three different allosteric networks for energy transduction, MT binding, and adenine association/dissociation in the motor domain; following functional expectation, they are interconnected by SCA.

**FIGURE 3.**
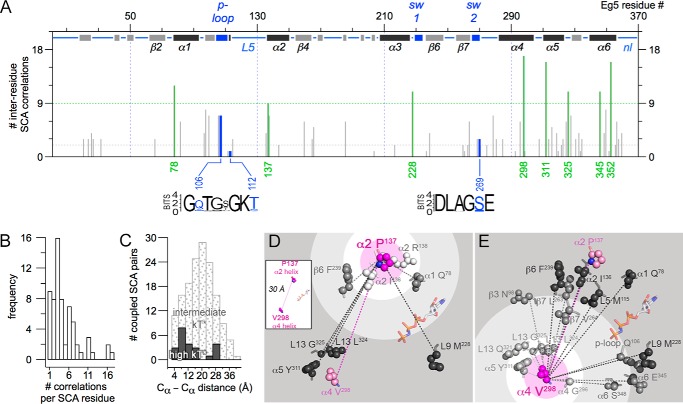
**Statistical correlations exist between multiple kinesin residues that are separated by large distances.** *A*, number of co-evolving correlations for each Eg5 residue in N- to C-terminal sequence order. Hub residues (≥9 correlations) are in *green*. SCA residues within the active site sequence WebLogos are in *blue*. Abbreviations: *sw1*, switch I; *sw2*, switch II; *nl*, necklinker. *B*, bar graph of the number of correlations per SCA residue. *C*, histogram of C_α_–C_α_ distances between coupled pairs of residues. Intermediate statistical couplings (1–1.39 kT*) are in *dotted gray*; high coupling (1.4–2.4 kT*) is shown in *black. D* and *E*, structural view of SCA residues correlated with Pro^137^ (*D*) or (*E*) Val^298^ in a kinesin-5 crystal structure (Protein Data Bank code 3HQD). Overlaid *concentric circles* delineate C_α_–C_α_ distances of 4 Å (*pink*), 10 Å (*white*), 20 Å (*light gray*), and 30 Å (*dark gray*) from residue of focus. Side chains and C_α_ atoms are shown as *spheres* and *sticks*, respectively; *dashed lines* indicate that two residues are statistically correlated. Residues are labeled with amino acid identity, Eg5 number, and secondary structure motif that they occupy.

We expected that the strict sequence conservation of active site loops (33) would bar these switch loops from co-evolution discovery. Switch SCA residues identified are known variable positions (*X*) in the sequence consensus or are residues with greater sequence variability than previously appreciated. SCA detects co-evolution between motor domain residues and two positions in the kinesin p-loop, GQT*XX*GK(S/T), and in switch II, D*XX*G*X*E (underlined here; Fig. 3*A*, in *blue* in WebLogos). Our data suggest that active site motifs contain classically defined, strictly conserved residues that are critical for active site chemistry that generate catalytic free energy (2) and variable SCA positions that communicate allosterically with the rest of the motor domain.

Our SCA model links residues in the active site with the MT-binding site, necklinker, and drug-binding L5 loop (Table 1 and Fig. 3*A*). SCA residues are highly clustered in the MT-binding site, including three helices that comprise the binding surface (α4, α5, and α6), as well as the surrounding loops. The first residue in both the neck linker and L5 are identified by SCA; additional residues in these loops are not easily detectable by SCA analyses, as they are indels and highly variable. Thus, our map of kinesin energetic architecture is complete for the core motor domain but will not include residue positions, centrally located in variable loops, that are potentially a significant, modular source of functional divergence.

##### Higher Order Co-evolutionary Coupling and Large Inter-residue Distances Were Common in the Kinesin Allosteric Network

The number of statistical correlations per kinesin SCA residue ranges from 1 to 17 (Fig. 3, *A* and *B*). Nine kinesin positions are statistically paired to only one other motor domain residue (Fig. 3*B*). The remaining SCA residues co-evolve with more than one kinesin residue, with a mode of three statistically correlated positions per residue. Eight residues are statistically correlated with at least nine other residues and are classified as hub residues (Fig. 3*A*, *green lines*). We conclude that higher order coupling is prevalent in kinesin SCA residues.

Also, we determined the C_α_–C_α_ inter-residue distances between coupled SCA residues (Fig. 3*C*); they ranged from those in close proximity to those that span the motor domain radius. The shortest inter-residue C_α_–C_α_ distances measured (4–6 Å) arose from nearest neighbors in the linear sequence (residues *i* and *i* + 1) or as positional neighbors within a helical turn (residues *i* and *i* + 4). We point out that inter-residue distances >6 Å are defined in this work as distal, as there is no spatial contact in tertiary structure. There is only one exception: Arg^26^/Glu^32^ (C_α_–C_α_ *d*_ij_ **=** 11.9 Å) participates in a direct interaction. The remaining residue pairs either do not have side chains arranged appropriately for an interaction or do not have compatible side chain groups.

Our measurements (Fig. 3*C*) showed that 90% of the statistical correlations between kinesin residue pairs are distal to one another. For residue pairs with intermediate levels of statistical coupling (ΔΔ*G*_stat_ = 1.0–1.39 kT*), the frequency of C_α_–C_α_ inter-residue distances had a normal distribution (Fig. 3*C*, *dotted fill*). Residue pairs with high co-evolutionary coupling (ΔΔ*G*_stat_ = 1.4–2.4 kT*) exhibited a bimodal distribution of inter-residue distances: those separated by 4–16 and 24–28 Å (Fig. 3*C*, *black fill*).

The relationship between number of and distance between correlated residues is illustrated using Pro^137^ and Val^298^, a pair of co-evolving residues in our analysis. These two SCA residues are located on opposite sides of the motor domain, separated by 30 Å (Fig. 3*D*, *inset*). Because each of these residues is a hub, there are 17 other residues that co-evolve with Pro^137^ and/or Val^298^. There are two groups of residues that correlate with these hub positions. The first group is a small set of residues proximal to Pro^137^ or Val^298^. Proximal residues are nearest neighbor contacts separated by 4–6 Å (Ile^136^ and Arg^138^ in Fig. 3*D* and Gly^296^ in Fig. 3*E*). Identification of such residue pairs that are in the immediate environment is consistent with expected local propagation of energy from a site of perturbation. The second group of correlated residues is comprised of residues at distances ≥6 Å. Correlated residues in the 6–11 Å range are not in direct contact with either Pro^137^ or Val^298^ (Phe^239^ and Gln^78^ in Fig. 3*D* and Leu^324^, Gly^325^, Gln^321^, and Tyr^311^ in Fig. 3*E*).

##### Statistical Coupling, Predicted by SCA, Was Tested by Mutagenesis of Residues Outside of the Active Site and Not in Contact with Each Other

The majority of SCA and residue co-evolution studies do not have experimental validation; these include prior analyses of motor proteins (35, 36). Although there is support that statistically dependent residues can be distant in space (37–40), only 2 of the >30 published SCA models (supplemental Table S1) provide experimental evidence that co-evolving residues pairs are energetically coupled (17, 41). Second, it is argued that energetic coupling is not a unique property to co-evolving residues (42).

**FIGURE 4.**
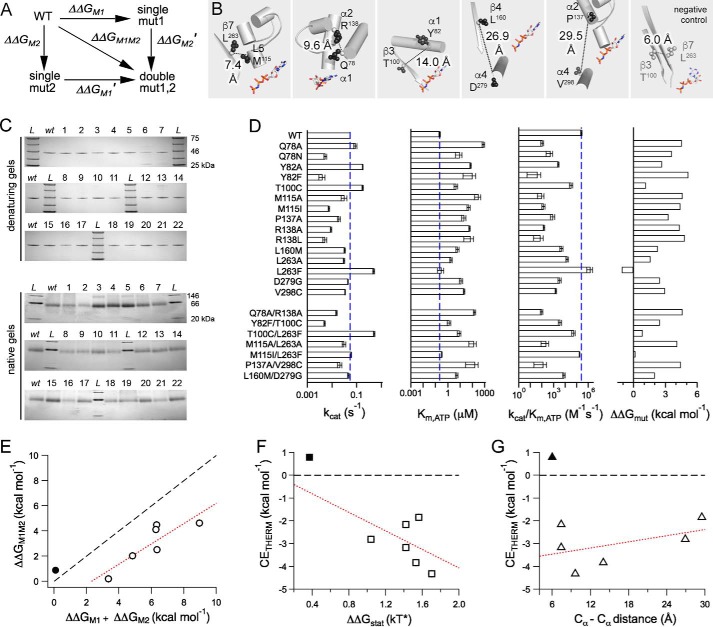
**The allostery wiring map was experimentally validated in human kinesin-5.** *A*, double-mutant cycle analysis scheme. *B*, statistically coupled residue pairs chosen for mutagenesis. Eg5 residue side chains (*spheres*) and C_α_–C_α_ distances are shown in Protein Data Bank code 3HQD; the AMPPNP molecule is a visual reference to location of active site. *C*, purified motor proteins were analyzed by denaturing and native gel electrophoresis. *Lane L*, molecular weight ladder; *lane wt*, wild type human Eg5 motor domain; *lane 1*, Q78A; *lane 2*, Q78N; *lane 3*, Y82A; *lane 4*, Y82F; *lane 5*, T100C; *lane 6*, M115A; *lane 7*, M115I; *lane 8*, P137A; *lane 9*, R138A; *lane 10*, R138L; *lane 11*, L160M; *lane 12*, L263A; *lane 13*, L263F; *lane 14*, D279G; *lane 15*, V298C; *lane 16*, Q78A/R138A; *lane 17*, Y82F/T100C; *lane 18*, T100C/L263F; *lane 19*, M115A/L263A; *lane 20*, M115I/L263F; *lane 21*, P137A/V298C; *lane 22*, L160M/D279G. *D*, ATP hydrolysis rate (*k*_cat_), nucleotide affinity (*K_m_*_,ATP_), and catalytic efficiency (*k*_cat_/*K_m_*_,ATP_) for wild type and mutant Eg5. Shown are averages of 2–7 measurements, and *error bars* represent standard deviation. These data were used to calculate ΔΔG of ATP hydrolysis. *E*, ΔΔ*G* for the double mutant is plotted against the sum of ΔΔ*G* for the corresponding two single mutants for our negative control (●) and the SCA residue pairs (○). *F*, correlation between experimentally derived, thermodynamic coupling energy (CE_THERM_) and statistical coupling energy (ΔΔ*G*_stat_) calculated by SCA. *G*, CE_THERM_ is plotted against C_α_–C_α_ distance between a residue pair. For *E–G*, the *black dashed line* indicates an additive relationship, and the *red dashed line* is fit to the experimentally derived data.

Thus, our next step was to determine whether the SCA residue network maps a path for catalytic free energy transduction. Practical considerations preclude testing a full map of all energetic interactions in a kinesin. To experimentally test all residue correlations predicted by SCA, 930 mutations and a minimum of 34,000 assays would be required. Six statistically coupled residue pairs, defined by SCA, and one negative control pair (T100C/L263F) were chosen (Table 2). Testing of six coupled residue pairs has a statistical power >95% and a *p*-value of 0.01. Our negative control residue pair has an associated 90% statistical power and a *p*-value of 0.05. The selected residue pairs had a wide range of C_α_–C_α_ distances (50% of the 10 bins in Fig. 3*C*) and vary widely in the number of residues to which they are coupled (54% of the 13 bins in Fig. 3*B*). They also vary in their location within the motor domain (Table 2 and Fig. 4*B*). However, none are located within active site motifs, and therefore, the experimental measurements that follow in this work would not arise from binding substrate or product. They also cannot be derived from changes in catalytic ability in the active site.

**TABLE 2:**
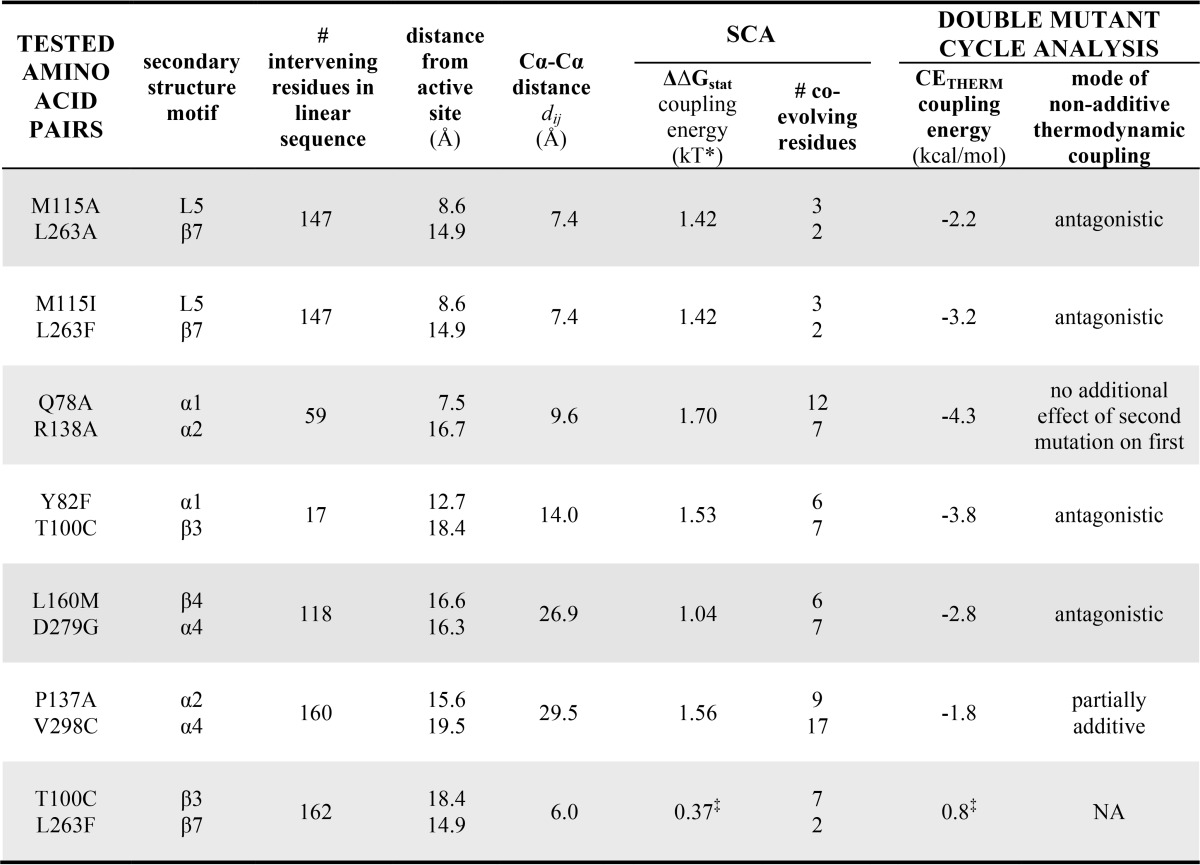
**Kinesin residues chosen for testing correlation between C_α_–C_α_ inter-residue distance and energetic coupling** Human kinesin 5 (Eg5) residue numbers are listed along with the secondary structure motif they occupy. The C_α_–C_α_ distances were measured within Protein Data Bank code 3HQD (2.2 Å resolution; Ref. 2) in PyMOL. A statistical coupling energy is given for each tested residue pair, as determined by SCA. Interaction energy (CE_THERM_) was determined from experiment; NA, not applicable. ‡ indicates the residue pair, Thr^100^-Leu^263^, served as a negative control, as confirmed by the low ΔΔG_stat_ and CE_THERM_ values.

Our last consideration was whether non-additivity is related to choice of amino acid substitutions. Thus, we generated two mutations per residue. One substitution was alanine, which typically replaces the original residue with a smaller one and minimizes the possibility of new interactions being formed. The second substitution was a naturally occurring substitution, culled from other kinesin motor domain sequences.

The established method for experimentally testing thermodynamic coupling is double-mutant cycle analysis (Fig. 4*A* and Refs. 17 and 43–45). If the change in free energy (ΔΔ*G*) for two single mutants is additive and equal to that of the corresponding double mutant for a residue pair, the residues are independent and therefore not coupled. However, if these are not equivalent, the residues are thermodynamically linked.

Catalytic activity (46, 47) was our Δ*G* readout; our model system was human Eg5 (Fig. 4*B*). This first assessment of energetic linkages in the kinesin motor domain was performed in the absence of MTs. However, we recognize that interaction with the cytoskeletal track may be an important source of evolutionary pressure on energy transduction. Mutations in Eg5 were generated and bacterially expressed (Table 2). T100A, Q78N/R138L, and Y82A/T100A had solubility issues and were not studied further. Wild type and mutant Eg5 proteins were purified to near homogeneity, as is evident from denaturing SDS-PAGE (Fig. 4*C*). In native gel electrophoresis, the 22 mutant proteins in this study had similar gel mobility to wild type (Fig. 4*C*), suggesting that mutations did not result in significant changes in the protein fold.

##### Substitution of SCA Network Residues Impacted Catalytic Parameters

Both single-site and double-site mutation of human kinesin-5 resulted in variable effects on ATP hydrolysis activity (Fig. 4*D*). To determine how well kinesin mutants bind substrate and how quickly substrate is converted into product once bound, ATP hydrolysis was measured as a function of nucleotide concentration (*n* = 2–7; data not shown). For the substitutions tested herein, 1242 assays were required. Non-linear regression analysis (Equation 1 under “Experimental Procedures”) was used to calculate *k*_cat_ and *K_m_*_,ATP_. Our *k*_cat_ and *K_m_*_,ATP_ values for wild type Eg5 (Fig. 4*D*, *vertical blue dashed lines*) are comparable with other reports (46, 48).

Although all the SCA residues tested in this study were distal from the active site (Table 2 and Fig. 4*B*), we observed a range of effects on the catalytic constant *k*_cat_. Of our single-site mutations, four substitutions had increased *k*_cat_, compared with the wild type Eg5 *k*_cat_ (0.05 ± 0.00 s^−1^; Fig. 4*D*, *vertical blue dashed lines*). The turnover numbers for Y82A (0.17 ± 0.00 s^−1^) and T100C (0.17 ± 0.01 s^−1^) were 3-fold greater than wild type (Fig. 4*D*); the *k*_cat_ for L263F (0.49 ± 0.04 s^−1^) was 8-fold greater than wild type. Ten single-site substitutions exhibited decreased *k*_cat_: Q78N, Y82F, M115I, M115A, P137A, R138A, R138L, L160M, L263A, and V298C. The rate for D279G was closest to wild type. Four mutants (M115A, L160M, L263A, and V298C) had turnover rates that were approximately half that of wild type Eg5. Y82F had the lowest measurable *k*_cat_ (0.004 ± 0.001 s^−1^), a 15-fold decrease from its parent protein.

Of the double mutants, the lowest turnover rate was 0.005 s^−1^ for Y82F/T100C. The highest was 0.51 ± 0.03 s^−1^ for T100C/L263F (Fig. 4*D*). The *k*_cat_ for Q78A/R138A, P137A/V298C, and M115A/L263A ranged between 0.02 and 0.03 s^−1^. The M115I/L263F and L160M/D279G kinesin samples had nearly wild type *k*_cat_ rates.

Despite the range of increased, neutral, and lowered ATP hydrolysis rates observed above, mutation of residues distant from the active site decreased the kinesin-5 efficiency in converting substrates into products. Catalytic efficiency (*k*_cat_/*K_m_*_,ATP_) was reduced for all substitutions except L263F (Fig. 4*D*). This was largely a result of increased values for the Michaelis constant for all substitutions (*K_m_*_,ATP_ in Fig. 4*D*); mutation, regardless of residue location or number per protein, resulted in 2–4,000-fold reduction in nucleotide affinity. L263F and M115I/L263F had similar *K_m_*_,ATP_ to wild type.

Wild type Eg5 had a specificity constant of 2.5 × 10^5^ m^−1^ s^−1^. The L263F single mutant exhibited a 10-fold increase in catalytic efficiency (1.6 × 10^6^ m^−1^ s^−1^), whereas the M115I/L263F double mutant retained a catalytic efficiency of 1.8 × 10^5^ m^−1^ s^−1^. Eleven substitutions resulted in 1000–10,000-fold reduction in catalytic efficiency (Fig. 4*D*): R138L, R138A, P137A, M115I, M115A, Y82F, Q78N, Q78A, P137A/V298C, M115A/L263A, and Q78A/R138A.

##### Most Tested Mutations Were Allosteric and Introduced Energy Barriers to Kinesin ATP Hydrolysis

Using the determined catalytic efficiencies above, we then calculated the change in free energy for generation of single-site mutations (ΔΔ*G*_M_) and for double mutations (ΔΔ*G*_M1M2_ in Equation 2 under “Experimental Procedures” and in Fig. 4*D*). One double mutant, M115I/L263F, had a ΔΔ*G*_M1M2_ of 0.2 kcal m^−1^, or effectively 0. One single substitution, L263F, had a ΔΔ*G*_M_ of −1.1 kcal m^−1^ (Fig. 4*D*). Thus, L263F is a facilitating mutation that decreased free energy barriers for catalysis, compared with wild type kinesin-5. Our results demonstrate that distant residues remote from the active site can have marked effects on catalysis.

##### Double-mutant Cycle Analysis Validated Thermodynamic Linkage for SCA Residue Pairs

In Fig. 4*E*, we show a scatter plot for changes in free energies of catalysis for double mutants against the sums of free energy changes for two single mutants. Data points that fall on the *dashed black line* would be additive and thus not thermodynamically linked. Non-additive pairs may fall above or below the 1:1 *dashed black line*.

For our negative control (residues not predicted to co-evolve by statistical methods), the catalytic effect of the double mutation T100C/L263F is not appreciably greater than the sum of the effect of single mutations (Fig. 4*E*, *filled circle*). Fig. 4*E* shows that the thermodynamic coupling energy (CE_THERM_) for our negative control, T100C/L263F, is 0.89 kcal m^−1^. Coupling energies smaller than 1–1.5 kcal m^−1^ are essentially additive, if experimental error is considered (49). For kinesin residue pairs that were statistically predicted to co-evolve, the sum of the constituent single mutation effects was larger than the effect of the double mutant (Fig. 4*E*, *open circles*). The quantitative interpretation is that all six tested SCA residue pairs are non-additive and thus energetically coupled. For one pair of residues, we confirmed thermodynamic linkage with both substitution strategies (M115I/L263F and M115A/L263A). Moreover, our experimental data showed that non-additive effects followed a linear relationship, with the sum of ΔΔ*G*_M1_ and ΔΔ*G*_M2_ being 2.0 kcal m^−1^ greater than ΔΔ*G*_M1M2_ (Fig. 4*E, red dotted line*).

For kinesin-5, there is a direct relationship between predicted, statistical residue linkages and thermodynamic coupling in experiment. In Fig. 4*F*, we show that there is a linear correlation in this system between statistical coupling energy (ΔΔ*G*_stat_) and experimental, thermodynamic coupling energy (CE_THERM_ or the interaction energy of the double mutant compared with its single point counterparts; Equation 3 under “Experimental Procedures”). The more strongly statistically correlated residue pairs are also the most energetically coupled residue pairs. We conclude that thermodynamic linkages between kinesin residues were accurately predicted by SCA.

##### Residue Pairs, Even Separated by 30 Å Distances, Were Energetically Coupled

Double-mutant cycle analysis can be used to measure the strength of the intramolecular pairwise interactions. We investigated the correlation between non-additivity and the spatial distance between mutation sites. CE_THERM_, or the strength of the intramolecular interactions, slightly decreases as inter-residue C_α_–C_α_ distance increased from 7 to 30 Å (Fig. 4*G*). It is notable that even for *d*_ij_ = 30 Å, the coupling energy was still significant. Our experimental data provide support for long distance energetic coupling in the kinesin motor domain. Thermodynamic linkages are not simply due to nearest neighbor interactions.

## Discussion

### 

#### 

##### For the First Time, Our Phylogenetic Tree Partitions Clades into Accepted Kinesin-MT Functions

Current phylogenetic efforts in the literature have exploded because of the availability of computational power and access to enormous amounts of genomic data. Overall, there are three trends in such studies. The first is application of multiple data sources to address a given phylogenetic question; however, such sources often are conflicting, and each has its own level of sequence error. Second, different inference techniques, with independent strengths and handicaps, are employed. Third, algorithms based on either probabilistic or combinatorial methods can provide supporting or conflicting statistical values for nodes of a given tree topology.

Frequently missing in such efforts is evidence of whether these outcomes provide meaningful representations of protein sequences. Derived from maximum likelihood techniques, our tree has a unique topology that correlates kinesin function with sequence organization. This improvement is nontrivial, because this phylogenetic inference model is built from a large sequence dataset of 78 individual taxa, nearly doubling the prior number of taxa evaluated. Such tree and sequence alignment outcomes exceed sole reliance on measures of statistical support (*i.e.* bootstrap proportions, Bayesian posterior probabilities).

Improvements in bioinformatics models and phylogenetic methods have far-reaching impact. First, success of statistical coupling analysis or any other residue co-evolution study is absolutely predicated on the sequence input being robust. Our extensive efforts in sequence validation and multiple sequence alignment were critical in obtaining a molecular network map of kinesin allostery that had faithful agreement between calculation and experiment. In addition, robust phylogenetic inference models allow tracing of ancestral states, a future focus of our work.

##### Three Intramolecular Communication Pathways Are Conserved in the Kinesin Superfamily

We discovered an allosteric network of residues with high statistical free energy (ΔΔ*G*_stat_); they are organized in a continuous path across the kinesin motor domain (Fig. 2*B*). Our work showed that statistical free energies of five pairs, or ten residues, in the network model were correlated with free energy of catalysis in one kinesin: thermodynamic coupling between altered kinesin residues (CE_THERM_ or energy linkage) was directly measured and found to be non-additive. Most reported double-mutant cycles make use of only one set of mutants. For example, statistical free energies of PDZ domains were tested with thermodynamic free energies of residue interactions for one residue position of one of the PDZ domains. Our work confirms that the SCA-identified communication pathway is based on energetic coupling of catalytic free energy. Our work also shows that energetic coupling is a unique property to co-evolving residues.

Our data support the conclusion that thermodynamic coupling across long distances dominates how energy is transduced to different motor domain sites. Several studies in the literature support our findings. Long range non-additivity is expected in allosteric proteins (50) but also is common in many protein classes (51). In addition, energy in a protein can jump from site to site, covering large distances (52). A complex arrangement of interactions between distant amino acids is prevalent. However, neither our data nor the data in Refs. 16 and 17 provide a structural mechanism for coupling or the time scale of communication propagation.

We also provide new information on the energetic value and mechanism of kinesin epistasis. Free energy departures from additivity measure the amount of cooperativity or anticooperativity between the effects of the two measured residues. Mechanisms for five possible interactions that beget non-additive relationships have been proposed (44). One set of mutants, P137A and V298C, exhibited a partially additive interaction (Table 2). Antagonistic, non-additive interactions were defined for four mutant pairs: Y82F and T100C, L160M and D279G, M115A and L263A, and M115I/L263F. For Q78A and R138A, the second mutation had no additional effect on the first mutation. None of our double-mutant pairs exhibited a synergistic interaction. Thus, only cooperative interactions were detected in the kinesin residue pairs tested. From Fig. 4*E*, we can estimate that the free energy of Eg5 catalysis has a cooperative contribution of *e*^2.0^ for our energy transduction residue pairs. We speculate that higher order coupling within the allosteric residue network underlies the cooperative effect across many residues, not just the tested residue pair. Lastly, our data argue that catalytic energy distribution in kinesin is not stochastically routed through the protein matrix.

##### In the Kinesin Motor Domain, the Intramolecular Residue Network for Energy Transduction Has a Unique Architecture That Allows Amplification to Multiple Sites

Energy dissipation in the kinesin motor is localized to discrete residues, both proximal and distant. We postulate that, even if only a few residues at the active site are energetically coupled, there are multiple destination sites for energy distribution, leading to signal amplification not only to the MT-binding site but also other sites. Conversely, signals from the surface of the motor domain can be reversed to the active site, which may serve as an efficient energy-accumulating center.

Our allosteric model provides a framework for kinesin energy transduction to localized nodes in the motor domain. For example, release of the hydrolysis product is essential for establishing a conformational cycle in which energy is converted to work; it may be triggered by changes in the ionic interaction with the β-phosphate, such as Lys^111^ in the p-loop (2). However, the exact nature of this signal is unknown.

Our energy transduction model points toward the adjacent Thr^112^ and Gln^106^ as conduits for energy transfer in the motor domain (Fig. 3*A*, WebLogo). Active site SCA residues are predicted to allosterically communicate with amino acids at a distance from the switch loops. Thr^112^ is energetically coupled to one residue, Lys^315^; Gln^106^ is coupled to six residues in the energy transduction sector (Fig. 2*C*, *yellow residue*). Furthermore, Ser^269^ in switch II is coupled to three residues.

Such coupling connects the active site with the MT-binding site, as well as the transducer and necklinker. The transducer, comprised of L5 and the central β-sheet, is proposed to relay information between the active and polymer binding sites via correlated conformational changes in both kinesins and myosins (46, 53–55). The necklinker is a flexible element that propels a motor head forward during processive stepping (56–58).

##### Improved Understanding of Allostery and Energy Transduction in Kinesins Has Broad Implications for the Mechanisms of Non-canonical Kinesins

**FIGURE 5.**
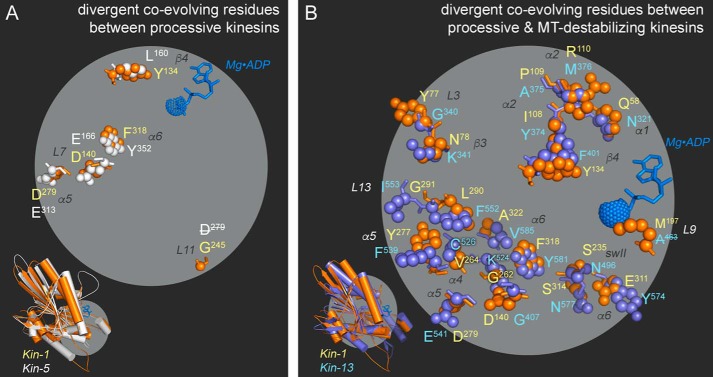
**Our kinesin mechanotransduction wiring map predicts residues that give rise to different nanomotor behaviors.** Co-evolving SCA residues that are divergent in amino acid identity between (*A*) human kinesin-1 (*orange*; Kif5b, Protein Data Bank code 1BG2) and human kinesin-5 (*white*; Eg5, Protein Data Bank code 1II6) and (*B*) human kinesin-1 (*orange*; Kif5b, Protein Data Bank code 1BG2) and human kinesin-13 (*blue*; Kif2C, Protein Data Bank code 2HEH). In both panels, the motor domain structures are aligned in the *insets* for orientation purposes. Residue number is listed alongside the corresponding secondary structure motif. A residue label with a *horizontal strike out* indicates a position that was unresolved in the crystal structure.

An important issue is whether the estimate of the statistical energies in one kinesin is transferable to another kinesin. Using our allosteric map, we compared the residue networks for two representatives in the kinesin superfamily. First, the mechanotransduction map of human kinesin-1 (Kif5b) was compared with that of human kinesin-5 (Eg5), two motors that are capable of processive motion, albeit to different extents and for different cellular purposes. Of the 65 SCA positions, 5 have a change in amino acid identity between these two motors (Fig. 5*A*); only 8% of the SCA positions are divergent and 92% of the SCA residue positions are identical between kinesin-1 and kinesin-5. Divergent residues are in β4 and the MT-binding site (L11/α4, α5, α6, and L7).

However, when we compare two motors at opposite ends of the mechanotransduction spectrum, the results are different. Between kinesin-1, a processive cargo transport motor, and kinesin-13, a non-processive MT-depolymerizing motor, 20 SCA positions have differing residues (Fig. 5*B*). This sequence divergence only occurs in the energy transduction sector; residues in the microtubule- and adenine-binding sectors are the same in human kinesin-1 and kinesin-13. Divergent SCA positions are found in the MT-binding site (along the length of α6, and clustered in α4 and α5), active site (switch II), various loops (L13, L3, and L9), the α1 and α2 helices, and β-sheet (β3 and β4). Divergence in energy transduction may be correlated with the different cellular functions of kinesin-1 and kinesin-13.

We submit that this work is significant on several fronts. This effort to advance our understanding of mechanotransduction provides practical rules for the rational engineering of motor function: prior rational design studies have tested 24 residues in our SCA network and measured perturbation to kinesin function (Table 1). A second possible avenue of use is prediction of which residues distal from the drug-binding L5 loop will confer resistance upon mutation. Lastly, it is our aspiration that it will assist us in defining the molecular basis for disease-causing mutations. Human polymorphisms are an *in vivo* test of our allosteric network. Disease-related SCA residues disrupt both network and cross-talk between allosteric networks and ultimately result in irreparable defects to mechanotransduction.

## Experimental Procedures

### 

#### 

##### Sequence Identification and Curation

A normal mode query of the SMART database for motor domain sequences from all members of the kinesin superfamily resulted in 3127 sequence hits. From this search, 1808 kinesin motor domain amino acid sequences that represent the entire superfamily were evaluated in this study. Misannotated sequences, fragments, and duplicates were removed to obtain a final dataset of unique, annotated sequences (supplemental Table S2). In the first check, sequences lacking a publication reference or cross-reference to NCBI were removed (*n* = 468 eliminated sequences). Second, sequences that were fragmented, incomplete at either the amino end or the carboxyl end or whose record was removed from NCBI were deleted from the dataset. Third, sequences were checked for duplications to remove syntenic homologs and other potential sources of noise. Using an in-house MATLAB (Mathworks) algorithm, the pairwise identity was then determined for each of the sequences, and those with greater than 95% identity were removed from the database (*n* = 525). The resulting data set contained 726 curated and unique kinesin sequences.

##### Computational Algorithms: SATé and SCA

The MSA and unrooted phylogeny were co-calculated in tandem using SATé version 1 (26, 59). The Holder laboratory (Kansas University) provided a software expansion that allowed amino acid sequences as input for SATé analysis. Parameters for SATé were: maximum subproblem size, 20% of data set; merger algorithm, OPAL (60); tree estimator, RAxML (61); number of CPUs used, 2; alignment algorithm, MAFFT (62); break strategy, centroid; and iteration limit, 20 after best maximum likelihood score. Run time for the SATé output was 70 days, and the maximum likelihood score was −302211. Phylogenies were visualized with Dendroscope (63). Rapid bootstrapping was conducted on the SATé output alignment using RAxML-HPCv8.2.8 (61, 64) on the CIPRES Science Gateway v3.3 (65) with 1000 iterations. Bootstrap confidence values were mapped onto the SATé tree using TreeGraph2 (66), and bootstrapped values ≥80% were annotated using iTOL v3.2.2 (67). Four supplemental files are provided. Supplemental Files S1 and S2 are the original and annotated SATé MSA files, respectively. Supplemental Files S3 and S4 are the original and annotated SATé phylogeny files. The SCA code was obtained from the Ranganathan laboratory and run with MATLAB; the input for SCA was the SATé-derived multiple sequence alignment of 726 kinesin motor domain sequences.

##### Structural Analyses

The crystal structures of kinesins (kinesin-5, 1II6 and 3HQD; kinesin-1, 1BG2; and kinesin-13, 2HEH); (2, 68, 69) were obtained from the Protein Data Bank and studied using PyMOL (Schrödinger). The criteria for residues that are nearest-neighbor, or in contact with one another, were defined as C_α_–C_α_ *d*_ij_ < 6 Å, and residues that are long range, or distal, are defined as *d*_ij_ ≥ 6 Å. A threshold value of 4 Å would be sufficient to eliminate residues interacting directly via van der Waals-like potentials. However, in a double-mutant protein, the new two residues might be in contact, depending on size differences in amino acid sidechains and relative orientations of their bonds. The value of 6 Å was chosen to ensure that the two residues are in contact neither in the wild type nor in the double-mutant structures.

##### Site-directed Mutagenesis, Bacterial Protein Expression, and Protein Purification

Wild type, human Eg5, encoding residues 1–369, in pET21d plasmid (Novagen) with a His_6_ tag served as the template (46, 70). Sixteen Eg5 single point mutations were generated for this study: Q78A, Q78N, Y82A, Y82F, T100A, T100C, M115A, M115I, P137A, R138A, R138L, L160M, L263A, L263F, D279G, and V298C. None of these residues are in the active site. Only one residue, Met^115^, contributes to the known allosteric loop 5. Thus, none of the chosen residues play a role in substrate binding, product release, or positioning of ligands. As such, perturbation of these residues tested their participation in distal (allosteric or indirect) effects on kinesin function.

Also generated were nine Eg5 double mutants: T100C/L263F, M115A/L263A, M115I/L263F, P137A/V298C, Q78A/R138A, Q78N/R138L, Y82A/T100A, Y82F/T100C, and L160M/D279G. Mutagenic primers (Integrated DNA Technologies) were designed in the 5′-3′ and 3′-5′ directions. Double mutants were generated using two rounds of mutagenesis. Following mutagenesis by PCR using Phusion polymerase (New England Biolabs), template DNA was digested with Dpn1 (New England Biolabs) at 37 °C, and unmethylated DNA was transformed into JM109 cells (Promega). All mutations were confirmed by DNA sequencing.

WT and all Eg5 mutants were expressed in BL21-Codon Plus (DE3)-RIL (Stratagene) in LB medium under ampicillin selection. Expression was induced at *A*_600 nm_ between 0.4 and 0.7 with 0.5 mm isopropyl β-d-thiogalactopyranoside. The cells were grown for either 5 h at 25 °C or overnight at 18 °C. The cells were harvested and pellets stored at −80 °C. The pellets were resuspended in 50 mm HEPES (pH 7.5), 75 mm NaCl, 1 mm PMSF, 0.1 mm MgATP, 1 mm DTT, 0.04 mg/ml DNase, and 0.6 mg/ml lysozome; the cells were lysed using an Emulsiflex (Avestin). Purification of WT and most mutant Eg5 proteins was performed by cation exchange chromatography. Protein was eluted with 250 mm NaCl as previously described (46). Final protein samples (>90% purity) were stored in 50 mm HEPES (pH 7.5), 125 mm NaCl, 0.1 mm NaATP, 0.1 mm MgCl_2_, 0.5 mm DTT, and 10% (v/v) glycerol. One exception to the purification strategy was M115A/L263A, which was purified by nickel affinity chromatography with a gradient elution (protein was eluted with 170 mm imidazole). The final protein sample was stored in 90 mm HEPES (pH 8.0), 45 mm NaCl, 0.9 mm NaATP, 1.8 mm MgCl_2_, 0.9 mm 2-mercaptoethanol, 153 mm imidazole, and 10% (v/v) glycerol. All protein samples were flash frozen and stored at −80 °C. Protein concentration was determined by Bradford assay (Coomassie Plus protein assay reagent; Thermo Scientific) with BSA as the standard. Concentrations ranged from 0.7 to 4.0 mg/ml or a purification yield of 3–20 mg/liter.

Samples were analyzed by SDS-PAGE using NuPage Novex 4–12% Bis-Tris gels (Invitrogen) and, under native conditions, using native PAGE Novex 4–16% Bis-Tris gels (Invitrogen). For denatured samples, SDS sample buffer was added with a final concentration of 62.5 mm Tris, pH 6.8, 10% glycerol, 100 mm DTT, 1.2% (w/v) SDS, and 0.01% (w/v) bromphenol blue. Samples were then boiled at 100 °C. Native samples were prepared in 1× NativePAGE sample buffer and G-250 additive (Invitrogen).

##### ATPase Activity Assays

Steady state kinetics of Eg5 were monitored using a NADH coupled enzymatic assay (46, 71) in a SpectraMax M2e spectrophotometer (Molecular Devices) in 96-well plate format (47). In measurements of basal ATP hydrolysis rates in the presence of saturating 1 mm MgATP, motor protein concentration was as follows: L263F and T100C/L263F, 0.625 μm; T100C and Y82A, 1.5 μm; WT, Q78A, M115A, L263A, M115A/L263A, M115I/L263F, P137A, V298C, and P137A/V298C, 2.5 μm; and R138A, M115I, Q78N, Y82F, R138L, Q78A/R138A, and Y82F/T100C, 5 μm. Measurements of 12 ATP concentrations, ranging from 238 nm to 1 mm MgATP, were used to determine the dependence of kinesin catalysis on ATP concentration. For these reported *k*_cat_ and *K_m_* experiments, 1242 assays were performed.

##### Kinetic Parameters and ΔG Calculations

ATP dependence data were averaged and plotted in IgorPro (WaveMetrics) using non-linear regression analysis:

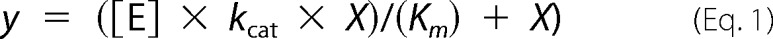


Experimentally determined ΔΔ*G* of mutation was calculated (43) as

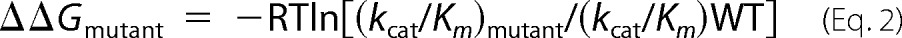


Coupling energy (CE_THERM_) from the double-mutant cycle analysis was determined as

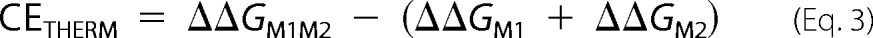
 in which M1M2 is the double mutant, and M1 and M2 are single-site mutants.

## Author Contributions

J. R. collected the kinesin sequences, manually curated the sequences, performed the SATé calculations, and analyzed all bioinformatic outputs; in addition, J. R. generated all the mutations, expressed and purified the proteins, and conducted all experimental benchwork. E. D. K. and C. D. K. performed the initial SCA analyses and wrote in-house algorithms for sequence evaluation. E. D. K. performed bootstrap analysis. H. N. assisted with obtaining activity data on four mutants in this report. S. K. conceived, designed, and coordinated the study and analyzed data with J. R. S. K. and J. R. wrote the manuscript and prepared the figures and tables. All authors reviewed the results and approved the final version of the manuscript.

## Supplementary Material

Supplemental Data

## Footnotes

2 The abbreviations used are:

MT: microtubule
L5: loop 5
SCA: statistical coupling analysis
NCBI: National Center for Biotechnology Information
SATé: simultaneous alignment and tree estimation algorithm
MSA: multiple sequence alignment
Eg5: human kinesin-5
p-loop: phosphate-binding loop
AMPPNP: adenosine 5′-(β,γ-iminotriphosphate).
