# Supplementary material for: Allostery Wiring Map for Kinesin Energy Transduction and Its Evolution
Source: J Biol Chem. 2016 Aug 8;291(40):20932–45. doi: 10.1074/jbc.M116.733675 (PMC5076506; doi:10.1074/jbc.M116.733675)
Supplement: Supplemental Data [file supp_291_40_20932__index.html]

Allostery Wiring Map for Kinesin Energy Transduction and Its Evolution — Energetic Coupling between Distant Kinesin Residues — Supplemental Data 

# Allostery Wiring Map for Kinesin Energy Transduction and Its Evolution

## Supplemental Data

**Files in this Data Supplement:**

- Supplement
- SI File 1
- SI File 2
- SI File 3
- SI File 4
