# Supplementary material for: Allostery Wiring Map for Kinesin Energy Transduction and Its Evolution
Source: J Biol Chem. 2016 Aug 8;291(40):20932–45. doi: 10.1074/jbc.M116.733675 (PMC5076506; doi:10.1074/jbc.M116.733675)
Supplement: Supplemental Data [file supp_M116.733675_Supplement.pdf]

SUPPLEMENT FOR
ALLOSTERY WIRING MAP FOR KINESIN ENERGY TRANSDUCTION AND ITS EVOLUTION

Jessica Richard, Elizabeth D. Kim, Hoang Nguyen, Catherine D. Kim, and Sunyoung Kim

From the Department of Biochemistry and Molecular Biology, Louisiana State University School of Medicine & Health Sciences Center, New Orleans, LA 70112

LIST OF FILES

Table S1. SCA has been used to analyze residue coevolution in 39 different protein families. The protein family analyzed by SCA, the model isoform used to analyze SCA results, the number of sequences in the MSA, and the number of amino acid residues in the model isoform are listed for each publication. We note whether a thermodynamic linkage was demonstrated between co-evolving residues.

Table S2. Database for 726 kinesin protein sequences analyzed. Sequences identifiers include NCBI sequence record number (GI), gene ID, and locus name. The table is sorted in alphabetical order by taxa. For each protein sequence, the number of amino acids (aa) in the full-length protein and motor domain residues are listed. Motor type is designated as N-terminal (N), middle (M), or C-terminal (C) to describe location of the motor domain within the full-length protein. References correspond to the sequencing projects or publications.

Figure S1. SATé produces better resolution in sequence alignments over Clustal. Portions of the full kinesin motor domain sequence alignment calculated with (A) SATé and (B) Clustal are shown as examples. A single sequence is a representative from each of the indicated kinesin families. Secondary structures are indicated at the top of each portion of the alignment. Gaps correspond to indels in the full sequence alignment.

Figure S2. Matrices for the energy transduction, MT-binding, and adenine-binding sectors. An enlarged view of the matrix in Fig 2 that corresponds to the (A) energy transduction, (B), MT-binding, and (C) adenine-binding sectors is provided so that the individual residues comprising each sector can be identified. The residues listed are from human Kinesin-5 as in column 1 of Table 1.

SI File 1. SATé-generated MSA (.zip file). The unzipped file is a .fasta file containing the SATé MSA of 726 kinesin motor domain sequences for use in the reader's own analyses. Sequence identifiers are the NCBI GI number and the range of residues reported for the motor domain.

SI File 2. Annotated Excel file of the SATé-generated MSA (.xlsx file). This file contains the SATé MSA of 726 kinesin motor domain sequences in annotated format. This file allows cross-referencing of SCA residues to other kinesin sequences or family members. The human kinesin-1, -5, -8, and -13 sequences listed in Table 1 are annotated in column A. NCBI GI numbers for each sequence along with the range of residue numbers reported for the motor domain are provided as sequence identifiers. Kinesin family numbers are assigned for each sequence; the alignment is ordered by by kinesin family number. Each position of the alignment is represented in a single cell and the MSA position number is annotated in the top row. SCA positions are indicated by grey highlight and the human Kinesin-5 residue number for each SCA position is listed. The human Kinesin-5 (GI:116242604) sequence is highlighted in blue , as it is the experimental model in the manuscript.

SI File 3. SATé-generated phylogenetic tree of 726 kinesin motor domain sequences (.zip file). The unzipped file is a .nex file of the unrooted kinesin phylogeny, determined by SATé. This file is provided for reader's use of our data in their own analyses. As in SI Files 1 and 2, sequence identifiers are the NCBI GI number and the residue range reported for the motor domain. This file also contains the values of all confidence measures as determined by rapid bootstrapping from 1000 replicates.

SI File 4. Annotated SATé generated phylogeny of 726 kinesin motor domain sequences (.pdf file). This file of the unrooted kinesin SATé phylogeny in circular representation. Generated from Interactive Tree of Life and downloadable from http://itol.embl.de/shared/SunyoungKimLab, this high resolution PDF file allows the reader to enlarge individual clades or branches of interest and examine the individual sequence annotations. In this pdf, families are colored and annotated with kinesin family nomenclature. As in the previous SI files, sequences are identified by their NCBI GI number, and residue range listed is defined by the public databases as to be inclusive of the motor domain; these sequences identifiers are listed along the perimeter of the phylogeny. Guides (dashed grey lines) are provided to visually assist matching individual tree leaves with the corresponding sequence identifier. Human kinesin-1, -5, -8, and -13 sequences, referenced in Table 1 of the main manuscript, are labeled in large white font and the guide lines are colored according to the representative kinesin family. Bootstrap values ≥80% are shown as grey circles on tree branches: smallest circles are 80% confidence measures, whereas the largest circles are 100% confidence values.

TABLE S1. SCA has been used to analyze residue coevolution in 39 different protein families. The protein family analyzed by SCA, the model isoform used to analyze SCA results, the number of sequences in the MSA, and the number of amino acid residues in the model isoform are listed for each publication. We note whether a thermodynamic linkage was demonstrated between co-evolving residues.

Protein family	Model isoform	Number of sequences	Number of residues	Demonstrated thermodynamic linkage	Ref
PDZ	$PDZ3^{psd-95}$	274	100	Yes	(1)
Class A GPCR	Bovine rhodopsin	940	348	No	(2)
Globin family (hemoglobin)	Human hemoglobin	880	560	No	(2)
Chymotrypsin family of serine proteases	Bovine trypsin	616	223	No	(2)
Guanine nucleotide binding proteins	Bovine G protein α subunit	717	353	No	(3)
Nuclear hormone receptor ligand binding domain	Human RXR	560	462	No	(4)
S1A family of serine proteases	Rat trypsin	1470	240	No	(5)
HSP70/110 chaperones	*E. coli* DnaK	926	605	No	(6)
Dihydrofolate reductase	*E. coli* DHFR	418	196	No	(7)
Cyclin dependent kinase inhibitor 2A and cyclin	Human CDKN2A and CDK6	159	427	No	(8)
WW domain	Nedd4.3	292	37	Yes	(9)
WW domain	Nedd4.3	120	36	No	(10)
GlycosylPhosphatidylInositol 12	*L. major* GlnNAC deacetylase		304		(11)
HIV-1 proteins (15 different proteins in total)		2000	23-553	No	(12)
GH1 β-glucosidases	*S. frugiperda* β-glycosidase	768	509	No	(13)
Voltage dependent K^+ channels	Shaker-IR	360	420	No	(14)
Kinesins		242	320	No	(15)
Myosins	*Dictyostelium* myosin II	709	761	No	(16)
Class II prolyl-tRNA synthetases	*E. co*li ProRS	492	567	No	(17)
Voltage dependent K^+ channels	Shaker	360	420	No	(18)
Antigen 85C in esterase	*M. tuberculosis* ag85C	846	121	No	(19)
Arsenic repressor	*S. aureus* CzrA	3000	140	No	(20)
ATP-binding cassette transporter family	Human CFTR	493	1480	No	(21)
Thiamine pyrophosphate-dependent enzymes	*E. coli* transketolase	382	680	No	(22)
Aspartokinase family	*C. glutamicum* aspartokinase	500	160	No	(23)
FtsZ	*M. jannaschii* FtsZ	223	350	No	(24)
Protein kinases	Human Pak2	482	250	No	(25)
Protein kinases	cAMP-dependent protein	1112	223	No	(26)
Leucyl-tRNA synthetase	T. thermophilus leuRS	484	876	No	(27)
Aspartic proteinase	*T. reesei* trichodermapepsin	1207	329	No	(28)
Ubiquitin-conjugating enzyme (E2)	UbcH5b	345	147	No	(29)
DNA cytosine C^5 methyltransferases	M.HhaI	389	327	No	(30)
Per/Arnt/Sim (PAS) family	*A. sativa* LOV2	1104	146	No	(31)
Dihydrofolate reductase	*E. coli* DHFR	418	159	No	(31)
Rhodopsin-like receptors (Class A GPCRs)	Human CXCR4	717	320	No	(32)
Plant peroxidases	Royal palm tree peroxidase	393	323	No	(33)
GroEL chaperonin		499	587	No	(34)
GroES cochaperonin		487	139	No	(34)
Membrane proteins				No	(35)

TABLE S2. Database for 726 kinesin protein sequences analyzed. Sequence identifiers include NCBI sequence record number (GI), gene ID, and locus name. The table is sorted in alphabetical order by taxa. For each protein sequence, the number of amino acids (aa) in the full-length protein and motor domain residues are listed. Motor type is designated as N-terminal (N), middle (M), or C-terminal (C) to describe location of the motor domain within the full-length protein. References correspond to the sequencing projects or publications.

GI	Gene ID	NCBI Prot ID	Protein Name	Taxa	No. aa in full length protein	Motor type (N/M/C)	Motor domain	Ref.
108869408	AAEL014084	EAT33633	Eg5	*A. aegypti*	1040	N	18-366	(36)
108872782	AAEL010942	EAT37007	KIF3A	*A. aegypti*	678	N	11-353	(36)
108872952	AAEL010803	EAT37177	Ncd	*A. aegypti*	637	C	297-628	(36)
108873164	AAEL010619	EAT37389	putative protein	*A. aegypti*	1044	N	2-282	(36)
108873223	AAEL010568	EAT37448	KIF4A	*A. aegypti*	1173	N	8-352	(36)
108874708	AAEL009227	EAT38933	kinesin heavy chain	*A. aegypti*	896	N	4-352	(36)
108875071	AAEL008890	EAT39296	kinesin heavy chain	*A. aegypti*	588	N	2-333	(36)
108875436	AAEL008542	EAT39661	kinesin heavy chain subunit	*A. aegypti*	931	N	3-295	(36)
108880159	AAEL004241	EAT44384	kinesin heavy chain	*A. aegypti*	397	N	51-375	(36)
108880160	AAEL004235	EAT44385	Klp10A	*A. aegypti*	718	M	241-581	(36)
108881536	AAEL002987	EAT45761	kinesin heavy chain	*A. aegypti*	1252	N	5-352	(36)
108881663	AAEL002877	EAT45888	KIF 17	*A. aegypti*	572	N	2-341	(36)
108882659	AAEL001986	EAT46884	KIF 1B	*A. aegypti*	1151	M	168-544	(36)
108882984	AAEL001648	EAT47209	kinesin family member 21	*A. aegypti*	1006	N	1-350	(36)
108883110	AAEL001582	EAT47335	kinesin	*A. aegypti*	1788	N	1-311	(36)
108883886	AAEL000822	EAT48111	KLP 68D	*A. aegypti*	788	N	15-351	(36)
108883941	AAEL000773	EAT48166	kinesin heavy chain	*A. aegypti*	698	N	95-443	(36)
122068686	5565492	Q17BU3	Kif 1A	*A. aegypti*	1644	N	2-358	(36)
154270889	HCAG_08619	XP_001536298	kinesin heavy chain	*A. capsulatus*	958	N	9-342	(37)
154272009	HCAG_07966	XP_001536857	putative protein	*A. capsulatus*	1174	N	77-422	(37)
154277914	HCAG_05255	XP_001539788	putative protein	*A. capsulatus*	812	N	2-388	(37)
154278860	HCAG_04083	XP_001540243	putative protein	*A. capsulatus*	1035	M	249-614	(37)
154279780	HCAG_04543	XP_001540703	putative protein	*A. capsulatus*	999	N	7-386	(37)
154283097	HCAG_02515	XP_001542344	putative protein	*A. capsulatus*	598	N	45-392	(37)
154284868	HCAG_00275	XP_001543229	putative protein	*A. capsulatus*	1674	N	6-373	(37)
154284952	HCAG_00317	XP_001543271	putative protein	*A. capsulatus*	1740	N	48-423	(37)
158284540	XM_307305	XP_307305	AGAP012471-PA	*A. gambiae*	683	N	5-347	(38)
158284863	XM_307936	XP_307936	AGAP002248-PA	*A. gambiae*	762	C	422-747	(39)
158285385		XP_308280	AGAP007592-PA	*A. gambiae*	1944	N	3-356	(39)
158285565	XM_308374	XP_308374	AGAP007502-PA	*A. gambiae*	1092	N	22-371	(39)
158286488	XM_308782	XP_308782	AGAP006989-PA	*A. gambiae*	632	N	80-466	(39)
158286815	XM_308943	XP_308943	AGAP006803-PA	*A. gambiae*	937	N	7-345	(39)
158289228	XM_310979	XP_310979	AGAP000159-PA	*A. gambiae*	725	M	249-579	(39)
158289936	XM_311552	XP_311552	AGAP010396-PA	*A. gambiae*	781	N	17-344	(39)
158290992	XM_312517	XP_312517	AGAP002427-PA	*A. gambiae*	647	N	11-344	(39)
158295904	XM_557151	XP_557151	AGAP006472-PA	*A. gambiae*	2261	N	4-328	(39)
158296752	XM_317097	XP_317097	AGAP008357-PA	*A. gambiae*	1054	N	51-292	(39)
158297149	XM_317425	XP_317425	AGAP008035-PA	*A. gambiae*	972	M	126-511	(39)

GI	Gene ID	NCBI Prot ID	Protein Name	Taxa	No. aa in full length protein	Motor type (N/M/C)	Motor domain	Ref.
158297459	XM_317685	XP_317685	AGAP007815-PA	*A. gambiae*	1033	N	17-361	(39)
158298183	XM_318377	XP_318377	AGAP003925-PA	*A. gambiae*	1268	N	2-365	(39)
158300830	XM_320651	XP_320651	AGAP011874-PA	*A. gambiae*	627	N	27-361	(39)
158563989	1275256	Q7PHR1	Kif1A	*A. gambiae*	1644	N	2-358	(39)
110294501		ABG66709	kinesin heavy chain 1	*A. californica*	979	N	6-326	(40)
1170619	827876	Q07970	Atk 1	*A. thaliana*	793	C	429-775	(41)
1170620	828826	P46864	Atk 2	*A. thaliana*	745	C	385-727	(42)
1170621	835556	P46875	Atk 3	*A. thaliana*	754	C	394-736	(43)
4314358	AC006340	AAD15569	putative kinesin heavy chain	*A. thaliana*	1068	M	425-727	(44)
4539324	AL035679	CAB38825	kinesin 4539324	*A. thaliana*	1121	N	98-488	(45)
4580395	AC007171	AAD24373	putative kinesin-like spindle protein	*A. thaliana*	1076	N	48-401	(44)
5541717	ATF28P10	CAB41097	kinesin 4678306	*A. thaliana*	1070	M	164-458	(46)
6056206	ATAC009400	AAF02823	putative kinesin-like centromere protein	*A. thaliana*	459	N	45-329	(46)
6523035	AL132976	CAB62303	kinesin 6523035	*A. thaliana*	1075	N	24-381	(46)
6526975	AB028468	BAA88112	kinesin 6526975	*A. thaliana*	823	N	3-325	(47)
6714287	AC013354	AAF25983	F15H18.10	*A. thaliana*	1162	M	619-972	(48)
7339486	AL162459	CAB82809	kinesin	*A. thaliana*	1058	N	46-399	(46)
7406433	AL162874	CAB85542	kinesin 7406433	*A. thaliana*	664	N	94-397	(49)
7573331	AL163818	CAB87801	kinesin heavy chain-like protein	*A. thaliana*	439	N	1-308	(46)
7594566	AL163975	CAB88133	kinesin 7594566	*A. thaliana*	1229	N	92-430	(46)
8745335	AF193768	AAF78894	phragmoplast-associated kinesin-related protein 1	*A. thaliana*	1292	N	92-426	(50)
11132972	818192	P82266	K125	*A. thaliana*	1056	N	10-368	(44)
15218418	NM_105884	NP_177370	kinesin	*A. thaliana*	1195	M	489-820	(48)
15237622	NM_123496	NP_198947	kinesin	*A. thaliana*	961	M	420-735	(49)
20197911	AC006841	AAD23684	AC006841_14 putative kinesin heavy chain	*A. thaliana*	1058	N	104-421	(44)
22327641	NM_124156	NP_199593	FRA1	*A. thaliana*	1035	N	10-371	(49)
22327992	NM_125486	NP_200901	putative kinesin	*A. thaliana*	1294	N	5-361	(49)
22329432	T12M4.14	NP_172389	kinesin	*A. thaliana*	1010	M	432-727	(48)
22331006	NM_112036	NP_187809	kinesin	*A. thaliana*	1030	N	69-388	(49)
22331291	NM_113271	NP_189009	kinesin 12B	*A. thaliana*	1313	N	97-431	(46)
30687506	NM_102022	NP_173592	kinesin (MKRP1)	*A. thaliana*	890	N	75-394	(48)
34921410	F2P16.12	O81635	Atk 4	*A. thaliana*	987	M	392-724	(49)
42565046	NM_112906	NP_188650	kinesin 42565046	*A. thaliana*	1114	M	103-436	(46)
75097784	dl3115c	O23274	kinesin	*A. thaliana*	1662	N	20-425	(45)
75173334	837799	Q9FZ06	Ark 3	*A. thaliana*	919	M	112-409	(51)
75277250	T32N15.10	O22240	putative kinesin	*A. thaliana*	767	M	288-600	(49)
75311662	830911	Q9LX99	GRIMP	*A. thaliana*	1273	M	142-454	(52)
75317980	At2g47500	O22260	putative kinesin heavy chain	*A. thaliana*	861	M	330-650	(44)
110737312	At2g37420	BAF00602	putative kinesin heavy chain	*A. thaliana*	1039	N	46-399	(53)
110739024	At1g73860	BAF01431	kinesin	*A. thaliana*	1025	M	507-846	(53)
145338627	NM_112614	NP_188362	POK1	*A. thaliana*	2066	M	167-505	(46)
145338697	NM_112791	NP_188535	POK2	*A. thaliana*	2771	M	192-530	(46)
186525204	NM_122296	NP_197779	ATP binding / MT motor	*A. thaliana*	739	N	54-314	(49)
193806750	At3g54870	Q9SV36	Ark 1	*A. thaliana*	1051	M	145-439	(51)
193806751	839306	Q9LPC6	Ark 2	*A. thaliana*	894	N	60-400	(51)
238481396	NM_122637	NP_198107	ATP binding / MTmotor	*A. thaliana*	762	M	127-456	(49)

GI	Gene ID	NCBI Prot ID	Protein Name	Taxa	No. aa in full length protein	Motor type (N/M/C)	Motor domain	Ref.
240254101	NM_101713	NP_173290	ATP binding / MT motor	*A. thaliana*	725	M	151-481	(48)
240254119	NM_101860	NP_173434	ATP binding / MT motor	*A. thaliana*	970	M	124-458	(48)
240256264	NM_120750	NP_196285	ATP binding / MT motor	*A. thaliana*	986	N	66-394	(49)
51701679	4619679	Q8J1G1	Kip 2	*A. gossypii*	685	M	113-446	(54)
45185914	4619762	NP_983630	ACR228Cp	*A. gossypii*	1129	N	52-425	(54)
121700144	ACLA_086080	XP_001268337	kinesin	*A. clavatus*	649	N	2-343	(55)
121700378	ACLA_087330	XP_001268454	kinesin heavy chain subunit	*A. clavatus*	929	N	9-330	(55)
121709389	ACLA_066170	XP_001272407	kinesin (BimC)	*A. clavatus*	1189	N	75-419	(55)
121710506	ACLA_091410	XP_001272869	kinesin	*A. clavatus*	1007	N	9-379	(55)
121711191	ACLA_005380	XP_001273211	kinesin (KipA)	*A. clavatus*	762	N	16-372	(55)
121713944	ACLA_016030	XP_001274583	kinesin	*A. clavatus*	655	N	92-449	(55)
121713972	ACLA_016170	XP_001274597	kinesin	*A. clavatus*	1748	N	49-440	(55)
121716118	ACLA_072750	XP_001275668	kinesin	*A. clavatus*	1633	N	6-370	(55)
70987448	XM_744044	XP_749137	Kip A	*A. fumigatus*	955	M	214-570	(56)
145236585	An06g01130	XP_001390940	An06c0070	*A. niger*	929	N	11-331	(57)
145238370	An07g07570	XP_001391832	An07c0220	*A. niger*	1010	N	6-376	(57)
145241043	An08g10640	XP_001393168	An08g10640	*A. niger*	751	N	2-351	(57)
145245976	An12g01500	XP_001395237	An12c0060	*A. niger*	1190	N	86-432	(57)
145247797	An13g00050	XP_001396147	An13g00050	*A. niger*	946	M	213-561	(57)
145249752	An14g05920	XP_001401215	An14g05920	*A. niger*	1750	N	49-442	(57)
145249804	An14g06180	XP_001401241	An14c0190	*A. niger*	658	N	93-450	(57)
145250765	An15g03580	XP_001396896	An15c0130	*A. niger*	1640	N	5-368	(57)
115387289	ATEG_01972	XP_001211150	putative protein	*A. terreus*	627	M	101-422	(58)
115389040	ATEG_02847	XP_001212025	putative protein	*A. terreus*	934	M	242-589	(58)
115389982	ATEG_03318	XP_001212496	putative protein	*A. terreus*	743	N	2-351	(58)
115399560	ATEG_06191	XP_001215369	putative protein	*A. terreus*	1745	N	29-421	(58)
115401696	ATEG_07815	XP_001216436	putative protein	*A. terreus*	1642	N	7-369	(58)
115442786	ATEG_09578	XP_001218200	putative protein	*A. terreus*	997	N	9-379	(58)
115491037	ATEG_00060	XP_001210146	putative protein	*A. terreus*	1175	N	75-425	(58)
156084037	BBOV_IV003380	XP_001609502	putative kinesin	*B. bovis*	618	C	252-544	(59)
156085767	BBOV_II007750	XP_001610293	putative kinesin	*B. bovis*	601	N	9-340	(59)
1170673		P46874	Klp 2	*B. mori*	378	N	11-347	(60)
118480522	EF092447	ABK92267	kinesin-like protein b	*B. mori*	482	N	4-325	(61)
118480530	EF092451	ABK92271	kinesin-like protein c	*B. mori*	489	N	58-454	(61)
182511222	NM_001123350	NP_001116822	kinesin-like protein KHC	*B. mori*	964	N	10-333	(61)
182511224	NM_001123351	NP_001116823	kinesin-like protein a	*B. mori*	627	M	160-492	(61)
187281809	NM_001126251	NP_001119723	kinesin-like protein Ncd	*B. mori*	620	C	277-608	(61)
76618834	XM_588573	XP_588573		*B. taurus*	1032	N	7-327	(62)
113911953	BC122794	AAI22795	similar to kinesin heavy chain isoform 5C	*B. taurus*	348	N	6-327	(62)
116248182	768014	Q2NL05	Kif 2A	*B. taurus*	660	M	176-505	(62)
145566785	777770	A0JN40	Kif 3C	*B. taurus*	792	N	9-363	(62)
155372019	NM_001101147	NP_001094617	kinesin family member 2C	*B. taurus*	723	M	255-584	(63)
158705863	538921	A6H750	Kif 2B	*B. taurus*	683	M	212-541	(62)
160707937	NM_001110788	NP_001104258	kinesin family member 3B	*B. taurus*	743	N	17-358	(64)
205815452	506294	A6QPL4	Kif 22	*B. taurus*	662	N	48-361	(62)
154289830	BC1G_15944	XP_001545520	putative protein	*B. fuckeliana*	1009	N	45-378	(65)
154291982	BC1G_14918	XP_001546569	putative protein	*B. fuckeliana*	733	N	2-331	(65)
154299063	BC1G_11844	XP_001549952	putative protein	*B. fuckeliana*	1008	M	243-601	(65)
154304427	BC1G_09089	XP_001552618	putative protein	*B. fuckeliana*	1846	N	7-372	(65)
154312475	BC1G_05840	XP_001555565	putative protein	*B. fuckeliana*	933	N	83-433	(65)
154314058	BC1G_04972	XP_001556354	putative protein	*B. fuckeliana*	1701	N	17-404	(65)
154319592	BC1G_02277	XP_001559113	kinesin heavy chain	*B. fuckeliana*	929	N	3-323	(65)

GI	Gene ID	NCBI Prot ID	Protein Name	Taxa	No. aa in full length protein	Motor type (N/M/C)	Motor domain	Ref.
1170664	176179	P34540	KINH	*C. elegans*	815	N	9-329	(66)
1176597	191697	P45962	Klp 3	*C. elegans*	598	C	244-565	(67)
1707073	U80450.1		kinesin-like protein 15	*C. elegans*	587	C	246-569	(67)
3874906	Z81048	CAB02839	C41G7.2	*C. elegans*	587	C	246-569	(67)
3878522	Z92807	CAB07264	K11D9.1a	*C. elegans*	747	M	320-648	(67)
3880931	Y43F4B.6	CAA16335	Klp 19	*C. elegans*	1083	N	5-325	(67)
17538506	C06G3.2	NP_501093	Klp 18	*C. elegans*	932	N	4-327	(67)
17554190	R144.1	NP_498069	Klp 6	*C. elegans*	928	N	5-365	(67)
17556094	NM_064777	NP_497178	Klp 20	*C. elegans*	646	N	5-331	(67)
17557420	NM_074181	NP_506582	bmk-1 (F23B12.8)	*C. elegans*	958	N	11-354	(67)
25144877	F20C5.2, F20C5.2a	NP_741473	putative Klp-11	*C. elegans*	782	N	12-343	(67)
25154708	K12F2.2	NP_506063	Kif 26L	*C. elegans*	1066	N	50-291	(67)
54042065	C52E12.2	P23678	Un 104	*C. elegans*	1584	N	2-354	(68)
56405341	177141	P46873	Osm 3	*C. elegans*	699	N	3-327	(69)
193206546	T01G1.1, T01G1.1c	NP_001122796	putative Klp-12	*C. elegans*	1580	N	4-359	(67)
3859666	AL033502	CAA22004	kinesin	*C. albicans*	665	N	76-403	(70)
1169693	XM_001701458	P46869	Fla 10	*C. reinhardtii*	786	N	9-353	(71)
1170672		P46870	Klp 1	*C. reinhardtii*	776	N	5-333	(72)
95061517	DQ499010	ABF50981	kinesin like calmodulin binding protein	*C. reinhardtii*	1238	C	850-1184	(73)
119174590	CIMG_09273	XP_001239652	putative protein	*C. immitis*	620	N	93-446	(37)
119175944	CIMG_09742	XP_001240121	putative protein	*C. immitis*	1850	N	99-493	(37)
119176882	CIMG_07460	XP_001240297	putative protein	*C. immitis*	1011	M	243-600	(37)
119189013	CIMG_04554	XP_001245113	putative protein	*C. immitis*	995	N	9-379	(37)
119196023	CIMG_02386	XP_001248615	putative protein	*C. immitis*	1658	N	7-370	(37)
119196989	CIMG_02869	XP_001249098	kinesin heavy chain	*C. immitis*	932	N	10-330	(37)
8488991		P70096	Kif 2C	*C. griseus*	718	M	251-580	(74)
81890110		Q60443	Kif C1	*C. griseus*	622	C	258-615	(75)
126644785	cgd3_2590	XP_001388114	K2	*C. parvum*	543	C	202-537	(76)
126644807	cgd3_3090	XP_001388120	putative protein	*C. parvum*	634	N	3-411	(76)
126649293	cgd6_1900	XP_001388318	putative kinesin heavy chain	*C. parvum*	714	N	75-447	(76)
126649295	cgd6_1920	XP_001388319	putative kinesin heavy chain	*C. parvum*	925	N	60-413	(76)
126649651	cgd6_4210	XP_001388347	putative boursin	*C. parvum*	1184	N	15-371	(76)
150036248	EF657230	ABR67411	kinesin	*C. melo*	791	M	138-461	(77)
82193823	544651	Q58G59	Kif 7	*D. rerio*	1363	N	14-348	(78)
94732475	BX248120	CAK04215	kinesin	*D. rerio*	753	C	351-679	(79)
113678678	NM_001044954	NP_001038419	kinesin family member C1	*D. rerio*	618	C	271-611	(79)
113678899	NM_001044976	NP_001038441	kinesin family member 14	*D. rerio*	1307	N	37-389	(79)
116284166	BC124345	AAI24346	Kif19 protein	*D. rerio*	524	N	11-346	(80)
116812563	NM_001077431	NP_001070899	kinesin family member 6	*D. rerio*	667	N	5-334	(80)
117558195	BC127399	AAI27400	Wu:fc51g12 protein	*D. rerio*	1048	N	18-357	(80)
123208287	DKEY-96L17.6-001	CAM15547	similar to vertebrate kinesin family	*D. rerio*	374	N	18-344	(79)
124481663	LOC100149074	AAI33148	putative protein	*D. rerio*	547	N	8-369	(80)
145388828	EF507508	ABP65301	Kif17	*D. rerio*	805	N	1-330	(81)
148725639	DKEY-38K3.2-001	CAN88221	kinesin	*D. rerio*	433	N	13-324	(79)
153945806	NM_001100145	NP_001093615	kinesin family member 3B	*D. rerio*	775	N	15-341	(80)
169145643	CR354377.9, CT573457.8	CAQ15503	novel protein (zgc:55995)	*D. rerio*	895	N	18-353	(79)

GI	Gene ID	NCBI Prot ID	Protein Name	Taxa	No. aa in full length protein	Motor type (N/M/C)	Motor domain	Ref.
169145918	CU463105	CAQ15674	similar to kinesin heavy chain member 2A	*D. rerio*	719	M	235-564	(79)
169158591	CR457482	CAQ15489	Kif 5a	*D. rerio*	850	N	6-325	(79)
189533414	XM_691034.3	XP_696126		*D. rerio*	663	N	9-341	(79)
205809507	561788	A8WFU8	Kif 22	*D. rerio*	634	N	19-343	(80)
292609530	XM_001337800	XP_001337836	similar to Kif19	*D. rerio*	846	N	12-349	(79)
66813336	XM_635755	XP_640847	K3	*D. discoideum*	1193	N	1-325	(82)
74837488	3398238	Q6S004	Kif 6	*D. discoideum*	1030	M	452-773	(83)
74853977	DDB_G0285101	Q54NP8	Kif 4	*D. discoideum*	1922	N	22-343	(82)
74855339	DDB_G0281555	Q54TL0	Kif 7	*D. discoideum*	1255	N	26-349	(82)
74913711	3388356	Q6RZZ9	Kif 13	*D. discoideum*	1265	N	21-359	(83)
74913713	3387029	Q6S001	Kif 11	*D. discoideum*	685	N	4-405	(83)
74913714	3385875	Q6S002	Kif 10	*D. discoideum*	1238	N	16-374	(83)
74933977	3394334	Q8T135	Kif 5	*D. discoideum*	990	N	4-330	(84)
74940780	3398234	Q9BPU3	Kif 2	*D. discoideum*	792	C	435-784	(85)
75024117	3386989	Q9NGQ2	Kif 1	*D. discoideum*	2205	N	1-362	(86)
127945	43517	P20480	NCD	*D. melanogaster*	700	C	346-672	(87)
128497	32107	P18105	NOD	*D. melanogaster*	666	N	8-318	(88)
1881662	DMU89264	AAB49460	klp67a	*D. melanogaster*	814	N	8-346	(89)
4972758	AF132186	AAD34774	no ID	*D. melanogaster*	1212	N	7-336	(90)
6942201	AF220353	AAF32355	CENP-meta	*D. melanogaster*	2244	N	8-321	(91)
6942203	AF220354	AAF32356	CENP-ana	*D. melanogaster*	1931	N	8-310	(91)
7298657	CG10718		Nebbish (Klp38B)	*D. melanogaster*	1121	M	119-525	(90)
7533198	AF247500	AAF63388	kinesin-like protein 7533198	*D. melanogaster*	1048	N	12-364	(92)
17647557	NM_079210	NP_523934	Kinesin-like protein at 64D, CG10642-PA	*D. melanogaster*	677	N	19-352	(93)
17864550	NM_080144	NP_524883	Klp 54D	*D. melanogaster*	844	M	168-514	(93)
19856508	36810	P17210	KINH	*D. melanogaster*	975	N	10-333	(94)
24659128	NM_137918	NP_611762	Klp59D, CG12192-PA	*D. melanogaster*	729	M	232-565	(93)
26006996	38135	P46863	Klp61F	*D. melanogaster*	1066	N	17-365	(95)
28573817	NM_135357	NP_609201	CG8183-PA, isoform A	*D. melanogaster*	1921	N	4-359	(93)
41688591	32049	Q960Z0	Klp 10A	*D. melanogaster*	805	M	277-608	(90)
41688598	37671	Q9W1U4	Klp59C	*D. melanogaster*	626	M	186-519	(90)
74948187	CG14535	Q9VLW2	Kif 26L	*D. melanogaster*	1131	N	44-391	(90)
119364607	39332	P46867	Klp 68D	*D. melanogaster*	784	N	18-344	(96)
158514035	36876	A1ZAJ2	Kif 1A	*D. melanogaster*	1670	N	2-358	(90)
160017392	35653	O16844	Cost 2	*D. melanogaster*	1201	M	132-3[illegible]	(97)
122064274	4812180	Q29DY1	Klp 68D	*D. pseudoobscura*	797	N	18-344	(98)
122109389	4805677	Q28WQ1	Kinesin-like protein unc-104	*D. pseudoobscura*	1671	N	2-358	(98)
198474507	4817387	XP_001356717	GA13060-PA	*D. pseudoobscura*	1171	N	40-395	(98)
221222444	4803492	Q292S8	Cost 2	*D. pseudoobscura*	1223	M	142-392	(98)
12044815	AJ291452	CAC19836	Kin A	*E. nidulans*	927	N	9-330	(99)
40974913	AJ620863	CAF06507	kinesin	*E. nidulans*	989	N	12-379	(100)
238054276	AN3363	P17120	BimC	*E. nidulans*	1184	N	79-424	(101)
149689859	XM_001502579	XP_001502629	KIF11	*E. caballus*	1056	N	16-368	(102)
149693713	XM_001496793	XP_001496843	kinesin family member 2C isoform 1	*E. caballus*	723	M	255-584	(102)
149714100	XM_001500023	XP_001500073	KIF21A	*E. caballus*	1786	M	126-484	(102)
149719535	XM_001504992	XP_001505042	kinesin family member 18A	*E. caballus*	895	N	11-355	(102)
149723689	XM_001488734	XP_001488784	kinesin family member 18B	*E. caballus*	860	N	16-351	(102)
149727714	XM_001502843	XP_001502893	No ID	*E. caballus*	792	N	9-364	(102)

GI	Gene ID	NCBI Prot ID	Protein Name	Taxa	No. aa in full length protein	Motor type (N/M/C)	Motor domain	Ref.
149733059	XM_001491846	XP_001491896	kinesin family member 16B	*E. caballus*	1314	N	2-365	(102)
149733181	XM_001500189	XP_001500239	KIF3B	*E. caballus*	747	N	18-340	(102)
149737083	XM_001499631	XP_001499681	kinesin family member 24	*E. caballus*	1367	M	222-544	(102)
149758455	XM_001488810	XP_001488860	kinesin family member 27	*E. caballus*	1405	N	4-342	(102)
194206515	XM_001495525	XP_001495575	kinesin family member 23	*E. caballus*	816	N	23-398	(102)
194206739	XM_001500620	XP_001500670	StARD9	*E. caballus*	4680	N	2-391	(102)
194207931	XM_001504329	XP_001504379	kinesin family member 17	*E. caballus*	1001	N	4-335	(102)
194212300	XM_001489282	XP_001489332	kinesin family member 5A	*E. caballus*	1024	N	7-327	(102)
194217548	XM_001502888	XP_001502938	kinesin family member 1C	*E. caballus*	1102	N	4-355	(102)
194218757	XM_001492229	XP_001492279	KIF 19	*E. caballus*	961	N	7-344	(102)
194219052	XM_001496313	XP_001496363	kinesin family member 22	*E. caballus*	651	N	36-349	(102)
194219937	XM_001502905	XP_001502955	kinesin family member 3A	*E. caballus*	702	N	13-345	(102)
194221381	XM_001495592	XP_001495642	kinesin family member 9	*E. caballus*	789	N	6-338	(102)
194222214	XM_001490166	XP_001490216	kinesin heavy chain isoform 5C	*E. caballus*	923	N	7-293	(102)
194223411	XM_001493478	XP_001493528	kinesin family member C1	*E. caballus*	675	C	310-668	(102)
194223853	XM_001493976	XP_001494026	kinesin heavy chain member 2A	*E. caballus*	660	M	176-505	(102)
194227062	XM_001493254	XP_001493304	similar to kinesin-1 heavy chain	*E. caballus*	960	N	18-322	(102)
194227280	XM_001491714	XP_001491764	Klp 6	*E. caballus*	1004	N	12-354	(102)
194227511	XM_001489250	XP_001489300	kinesin family member 25	*E. caballus*	477	C	123-462	(102)
194227999	XM_001491181	XP_001491231	kinesin family member 4A	*E. caballus*	1166	N	8-283	(102)
18202611	395823	Q90640	KIF4	*G. gallus*	1225	N	11-344	(103)
82197864	419968	Q5ZLK6	Kif 18B	*G. gallus*	797	N	24-352	(104)
126165270	NM_001081698	NP_001075167	XCTK2-like motor protein	*G. gallus*	647	C	295-640	(105)
158705889	427156	Q5ZKV8	Kif 2A	*G. gallus*	706	M	222-551	(104)
51316436		Q86Z98	KINH	*G. moniliformis*	931	N	4-329	(106)
127519339	EF432568	ABO28522	kinesin-related protein	*G. hirsutum*	1015	M	400-729	(107)
417216	3799	P33176	KINH	*H. sapiens*	963	N	6-325	(108)
3913957	3797	O14782	Kif 3C	*H. sapiens*	793	N	9-365	(109)
3913958	9371	O15066	Kif 3B	*H. sapiens*	747	N	18-340	(110)
13124319	3800	O60282	Kif 5C	*H. sapiens*	957	N	6-327	(111)
19863381	3835	Q14807	Kif 22	*H. sapiens*	665	N	60-366	(112)
20138710	3833	Q9BW19	Kif C1	*H. sapiens*	673	C	308-666	(113)
20138788	3834	Q9UIL4	Kif 25	*H. sapiens*	384	N	31-366	(114)
20141607	11004	Q99661	Kif 2C	*H. sapiens*	725	M	257-586	(115)
23396625	23303	Q9NQT8	Kif 13B	*H. sapiens*	1826	N	4-360	(116)
23396633	9928	Q15058	Kif 14	*H. sapiens*	1648	M	357-708	(117)
34098674	90990	Q96AC6	Kif C2	*H. sapiens*	838	M	407-707	(118)
34978376	57576	Q9P2E2	kinesin family member 17	*H. sapiens*	1029	N	4-335	(119)
50400977	55605	Q7Z4S6	Kif 21A	*H. sapiens*	1674	N	8-372	(120)
50403793	55614	Q96L93	Kif 16B	*H. sapiens*	1317	N	2-365	(121)
52001487	64147	Q9HAQ2	Kif 9	*H. sapiens*	790	N	6-338	(118)
59799772	23046	O75037	Kif 21B	*H. sapiens*	1637	N	7-371	(122)
66774137	81930	Q8NI77	Kif 18A	*H. sapiens*	898	N	11-355	(123)
74750464	55582	Q86VH2	Kif 27	*H. sapiens*	1401	N	4-342	(124)
74752937	56992	Q9NS87	Kif 15	*H. sapiens*	1388	N	25-363	(125)

GI	Gene ID	NCBI Prot ID	Protein Name	Taxa	No. aa in full length protein	Motor type (N/M/C)	Motor domain	Ref.
85541032	113220	Q96FN5	Kif 12	*H. sapiens*	646	N	25-358	(126)
116242604	3832	P52732	Kif 11	*H. sapiens*	1056	N	16-368	(127)
116242605	23095	O60333	Kif 1B	*H. sapiens*	1816	N	4-361	(128)
116242606	10749	O43896	Kif 1C	*H. sapiens*	1103	N	4-355	(129)
118572662	63971	Q9H1H9	Kif 13A	*H. sapiens*	1805	N	4-359	(130)
119364606	547	Q12756	Kif 1A	*H. sapiens*	1690	N	4-361	(131)
121948325	55083	Q2KJY2	Kif 26B	*H. sapiens*	2108	M	450-798	(118)
124056471	3801	Q9BVG8	Kif C3	*H. sapiens*	833	C	443-771	(132)
126215730	124602	Q2TAC6	Kif 19	*H. sapiens*	998	N	11-346	(118)
126215732	347240	Q5T7B8	Kif 24	*H. sapiens*	1368	M	222-544	(126)
143811412	3798	Q12840	Kif 5A	*H. sapiens*	1032	N	7-327	(133)
146345445	221458	Q6ZMV9	Kif 6	*H. sapiens*	814	N	5-343	(118)
158564279	285643	Q2VIQ3	Kif 4B	*H. sapiens*	1234	N	8-337	(134)
158931161	3796	O00139	Kif 2A	*H. sapiens*	706	M	222-551	(135)
160014128	26153	Q9ULI4	Kif 26A	*H. sapiens*	1882	M	371-723	(136)
160112933	KIAA1300	Q9P2P6	Star 9	*H. sapiens*	4614	N	2-384	(137)
160358869	1062	Q02224	CENPE	*H. sapiens*	2701	N	6-329	(138)
172045866	374654	Q2M1P5	Kif 7	*H. sapiens*	1343	N	14-350	(139)
182662392		Q86Y91	Kif 18B	*H. sapiens*	844	N	21-351	(118)
229462962		Q9Y496	Kif 3A	*H. sapiens*	702	N	13-345	(140)
251757291	84643	Q8N4N8	Kif 2B	*H. sapiens*	673	M	211-549	(118)
134061870	AM494958	CAM38907	putative kinesin	*L. braziliensis*	2140	M	183-470	(141)
154331027	XM_001561903	XP_001561953	kinesin	*L. braziliensis*	662	M	119-485	(141)
154331257	XM_001561397	XP_001561447	putative MCAK	*L. braziliensis*	663	M	127-445	(141)
154333802	XM_001563106	XP_001563156	putative MCAK	*L. braziliensis*	656	N	2-281	(141)
154334064	XM_001563237	XP_001563287	putative kinesin	*L. braziliensis*	729	M	207-516	(141)
154334291	XM_001563347	XP_001563397	putative K39	*L. braziliensis*	2155	N	36-413	(141)
154334303	XM_001563353	XP_001563403	putative kinesin	*L. braziliensis*	1078	N	66-486	(141)
154335017	XM_001563705	XP_001563755	putative kinesin	*L. braziliensis*	1037	M	167-481	(141)
154335216	XM_001563798	XP_001563848	putative OSM3	*L. braziliensis*	928	N	12-339	(141)
154335298	XM_001563839	XP_001563889	putative kinesin	*L. braziliensis*	1433	N	10-342	(141)
154335689	XM_001564033	XP_001564083	putative kinesin	*L. braziliensis*	791	N	5-315	(141)
154335834	XM_001564103	XP_001564153	C-terminal motor putative kinesin	*L. braziliensis*	839	C	500-802	(141)
154335920	XM_001564146	XP_001564196	putative kinesin	*L. braziliensis*	1097	M	218-557	(141)
154336383	XM_001564377	XP_001564427	putative kinesin	*L. braziliensis*	513	N	66-467	(141)
154336867	XM_001564619	XP_001564669	putative Unc104	*L. braziliensis*	1534	N	89-441	(141)
154337473	XM_001564919	XP_001564969	putative kinesin	*L. braziliensis*	893	N	9-441	(141)
154337545	XM_001564955	XP_001565005	putative kinesin	*L. braziliensis*	1432	M	183-523	(141)
154337834	XM_001565093	XP_001565143	putative kinesin	*L. braziliensis*	720	N	46-451	(141)
154338062	XM_001565207	XP_001565257	kinesin	*L. braziliensis*	1203	M	124-440	(141)
154338215	XM_001565282	XP_001565332	putative MCAK	*L. braziliensis*	578	N	3-322	(141)
154338866	XM_001565605	XP_001565655	Putative kinesin	*L. braziliensis*	1045	N	5-315	(141)
154338870	XM_001565607	XP_001565657	Putative kinesin	*L. braziliensis*	1053	N	84-440	(141)
154340994	XM_001566400	XP_001566450	Putative kinesin	*L. braziliensis*	1628	N	9-347	(141)
154341250	XM_001566528	XP_001566578	Putative kinesin	*L. braziliensis*	518	N	1-267	(141)
154341252	XM_001566529	XP_001566579	Putative kinesin	*L. braziliensis*	588	N	8-337	(141)
154341441	XM_001566622	XP_001566672	Putative kinesin	*L. braziliensis*	607	N	15-352	(141)
154341667	XM_001566735	XP_001566785	Putative kinesin	*L. braziliensis*	1201	N	5-354	(141)
154341957	XM_001566880	XP_001566930	Putative kinesin	*L. braziliensis*	1191	N	86-405	(141)
154342184	XM_001566990	XP_001567040	putative MCAK	*L. braziliensis*	881	M	247-560	(141)
154342975	XM_001567383	XP_001567433	putative OSM3	*L. braziliensis*	1118	N	10-336	(141)
154344132	XM_001567960	XP_001568010	putative kinesin	*L. braziliensis*	964	N	2-300	(141)
154344212	XM_001568000	XP_001568050	putative Unc104	*L. braziliensis*	2069	N	37-476	(141)
154344735	XM_001568259	XP_001568309	putative kinesin	*L. braziliensis*	694	N	9-416	(141)
154346438	XM_001569106	XP_001569156	putative protein	*L. braziliensis*	625	N	11-349	(141)
1170665		P46865	KINL	*L. chagasi*	955	N	11-399	(142)
112293605	DQ831678	ABI14928	K39 kinesin protein	*L. donovani*	3276	N	11-399	(143)
134069673	AM502240	CAM67997	putative kinesin	*L. infantum*	889	N	9-432	(141)
146074824	XM_001462580	XP_001462617	putative MCAK	*L. infantum*	673	M	131-449	(141)
146076877	XM_001462989	XP_001463026	putative kinesin	*L. infantum*	1069	N	1-289	(141)
146080601	XM_001464003	XP_001464040	putative MCAK	*L. infantum*	726	N	35-350	(141)
146081054	XM_001464143	XP_001464180	putative MCAK	*L. infantum*	728	M	196-515	(141)
146081490	XM_001464229	XP_001464266	putative kinesin	*L. infantum*	1307	M	264-591	(141)
146081615	XM_001464259	XP_001464296	putative K39	*L. infantum*	2461	N	36-412	(141)

GI	Gene ID	NCBI Prot ID	Protein Name	Taxa	No. aa in full length protein	Motor type (N/M/C)	Motor domain	Ref.
146081628	XM_001464262	XP_001464299	putative K39	*L. infantum*	2926	N	39-436	(141)
146082939	XM_001464598	XP_001464635	putative kinesin	*L. infantum*	608	M	144-458	(141)
146082943	XM_001464599	XP_001464636	putative kinesin	*L. infantum*	793	N	38-352	(141)
146082988	XM_001464610	XP_001464647	putative kinesin	*L. infantum*	1082	N	11-408	(141)
146083473	XM_001464711	XP_001464748	putative OSM3	*L. infantum*	940	N	12-339	(141)
146083578	XM_001464742	XP_001464779	putative kinesin	*L. infantum*	1430	N	10-342	(141)
146084251	XM_001464934	XP_001464971	putative kinesin	*L. infantum*	796	N	5-315	(141)
146084383	XM_001464952	XP_001464989	putative C-terminal kinesin	*L. infantum*	841	C	501-803	(141)
146084523	XM_001464993	XP_001465030	putative kinesin	*L. infantum*	1091	M	218-558	(141)
146084573	XM_001465006	XP_001465043	putative kinesin	*L. infantum*	1135	N	80-566	(141)
146086674	XM_001465573	XP_001465610	putative kinesin	*L. infantum*	1255	N	7-329	(141)
146087173	XM_001465710	XP_001465747	putative kinesin	*L. infantum*	656	N	7-387	(141)
146087748	XM_001465856	XP_001465893	putative MCAK	*L. infantum*	577	N	3-322	(141)
146089100	XM_001466197	XP_001466234	putative kinesin	*L. infantum*	1066	N	5-315	(141)
146089107	XM_001466199	XP_001466236	putative kinesin	*L. infantum*	1062	N	84-445	(141)
146092165	XM_001470186	XP_001470223	putative kinesin	*L. infantum*	965	C	613-956	(141)
146093025	XM_001466587	XP_001466624	putative kinesin	*L. infantum*	1665	N	25-385	(141)
146093319	XM_001466734	XP_001466771	putative kinesin	*L. infantum*	590	N	10-339	(141)
146093321	XM_001466735	XP_001466772	putative kinesin	*L. infantum*	589	N	8-337	(141)
146093540	XM_001466844	XP_001466881	putative kinesin	*L. infantum*	610	N	15-352	(141)
146093774	XM_001466961	XP_001466998	putative kinesin	*L. infantum*	1211	N	5-354	(141)
146094090	XM_001467119	XP_001467156	putative kinesin	*L. infantum*	1190	N	85-404	(141)
146094262	XM_001467205	XP_001467242	putative kinesin	*L. infantum*	2129	M	179-466	(141)
146094485	XM_001467254	XP_001467291	putative MCAK	*L. infantum*	951	M	320-633	(141)
146096205	XM_001467696	XP_001467733	putative OSM3	*L. infantum*	1117	N	10-336	(141)
146098051	XM_001468267	XP_001468304	putative kinesin	*L. infantum*	960	N	2-300	(141)
146098084	XM_001468277	XP_001468314	putative Unc104	*L. infantum*	2074	N	37-476	(141)
146099027	XM_001468498	XP_001468535	putative kinesin	*L. infantum*	1068	N	65-485	(141)
146101101	XM_001468989	XP_001469026	putative kinesin	*L. infantum*	698	N	9-417	(141)
146104762	XM_001469869	XP_001469906	putative protein	*L. infantum*	625	N	11-349	(141)
6855413	AL049768	CAB71236	kinesin	*L. major*	656	N	7-387	(144)
11071678	AL449123	CAC14606	kinesin	*L. major*	956	N	2-300	(144)
157878755	XM_001687343	XP_001687395	putative MCAK	*L. major*	668	M	133-451	(145)
149239258	XM_001525455	XP_001525505	putative protein	*L. elongisporus*	823	N	1-398	(146)
149240177	XM_001525914	XP_001525964	putative protein	*L. elongisporus*	840	M	131-510	(146)
149240816	XM_001526181	XP_001526231	putative protein	*L. elongisporus*	926	N	53-435	(146)
149246994	XM_001527872	XP_001527922	putative protein	*L. elongisporus*	972	N	58-395	(146)
125415		P21613	KINH	*L. pealei*	967	N	6-326	(147)
48474595	QtsA-16015	Q95LP1	Kif 2C	*M. fascicularis*	671	M	203-532	(148)
75066639	QtsA-10833, QtsA-13688, QtsA-18420	Q95LT1	Kif 2B	*M. fascicularis*	670	M	209-538	(148)
75076234	QtsA-19288	Q4R628	Kif 3A	*M. fascicularis*	702	N	13-345	(149)
109003659	XM_001093746	XP_001093746		*M. mulatta*	706	M	238-567	(150)
109073318	XM_001085776	XP_001085776		*M. mulatta*	601	C	186-583	(150)
109092967	XM_001086226	XP_001086226		*M. mulatta*	1317	N	2-365	(150)
109116126	XM_001102269	XP_001102269		*M. mulatta*	881	N	21-351	(150)
109128063	XM_001104446	XP_001104446		*M. mulatta*	671	N	49-355	(150)
39944768	XM_361921	XP_361921	putative protein	*M. grisea*	972	M	212-569	(151)
39973337	XM_368059	XP_368059	putative protein	*M. grisea*	733	N	2-330	(151)
145605477	XM_364410	XP_364410	putative protein	*M. grisea*	1771	N	4-360	(151)
145605820	XM_370342	XP_370342	putative protein	*M. grisea*	1068	N	40-373	(151)
145612205	XM_362573	XP_362573	putative protein	*M. grisea*	614	N	49-413	(151)
145613277	XM_363831	XP_363831	putative protein	*M. grisea*	937	N	4-329	(151)
145614060	XM_363249	XP_363249	putative protein	*M. grisea*	1115	N	27-370	(151)
122231692		Q1RYB8	kinesin-related protein tck1	*M. truncatula*	1254	C	873-1204	(152)
122231751		Q1S664	kinesin	*M. truncatula*	665	N	23-386	(152)
122237914		Q1SGR1	kinesin	*M. truncatula*	801	C	432-782	(152)
122244246		Q1S9J2	kinesin	*M. truncatula*	1073	N	87-414	(152)
122248245		Q1SEG1	plant kinesin-like protein	*M. truncatula*	1265	C	887-1218	(152)
124359768	AC150799	ABN06095	kinesin	*M. truncatula*	625	N	87-425	(152)
126307535	XM_001369332	XP_001369369	no ID	*M. domestica*	682	M	224-553	(153)

GI	Gene ID	NCBI Prot ID	Protein Name	Taxa	No. aa in full length protein	Motor type (N/M/C)	Motor domain	Ref.
126309847	XM_001377630	XP_001377667	no ID	*M. domestica*	770	C	402-763	(153)
126323074	XM_001372120	XP_001372157	no ID	*M. domestica*	820	C	437-768	(153)
126335571	XM_001368067	XP_001368104	no ID	*M. domestica*	670	N	61-366	(153)
126341392	XM_001374859	XP_001374896	no ID	*M. domestica*	1054	N	6-325	(153)
126341614	XM_001379339	XP_001379376	no ID	*M. domestica*	1399	N	25-364	(153)
126342775	XM_001368376	XP_001368413	no ID	*M. domestica*	1231	N	8-339	(153)
1762638	MSU64819	AAB39558	microtubule-based motor protein	*M. saxatilis*	773	C	369-690	(154)
125403		P28741	Kif 3A	*M. musculus*	701	N	13-345	(155)
1170659		P33174	Kif 4	*M. musculus*	1231	N	8-335	(156)
1944330	D49545	BAA19677	Kif C2	*M. musculus*	792	C	407-735	(157)
2497519		Q61768	KINH	*M. musculus*	963	N	6-325	(158)
2506794	NM_008440	P33173	Kif 1A, isoform A	*M. musculus*	1695	N	4-361	(159)
3122327		Q61771	Kif 3B	*M. musculus*	747	N	18-340	(160)
3913959		O35066	Kif 3C	*M. musculus*	796	N	9-367	(161)
6979905	AF221102	AF221102_1	KIFC5A	*M. musculus*	674	C	309-667	(162)
9297100		P28738	Kif 5C	*M. musculus*	956	N	6-327	(163)
12644454		Q60575	Kif 1B	*M. musculus*	1816	N	4-361	(164)
18203576	16578	Q9WV04	Kif 9	*M. musculus*	790	N	6-338	(165)
23396622		Q9EQW7	Kif 13A	*M. musculus*	1749	N	4-359	(166)
23396634	16559	Q99PW8	Kif 17	*M. musculus*	1038	N	4-335	(167)
29840788		Q922S8	Kif 2C	*M. musculus*	721	M	253-582	(118)
32699612	16552	Q9D2Z8	Kif 12	*M. musculus*	642	N	25-358	(168)
50401186	16565	Q9QXL1	Kif 21B	*M. musculus*	1668	N	7-372	(169)
50401187	16564	Q9QXL2	Kif 21A	*M. musculus*	1672	N	8-372	(169)
68570276	228421	Q91WD7	Kif 18A	*M. musculus*	886	N	11-355	(168)
77416870	16562	O35071	Kif 1C	*M. musculus*	1100	N	4-355	(165)
81892355	209737	Q6P9L6	Kif 15	*M. musculus*	1387	N	25-363	(170)
81892361	16551	Q6P9P6	Kif 11	*M. musculus*	1052	N	15-367	(118)
81892832	229841	Q6RT24	CENPE	*M. musculus*	2474	N	6-329	(171)
81894342	75050	Q7M6Z4	Kif 27	*M. musculus*	1394	N	4-342	(118)
81898327	73470	Q8C0N1	Kif 2B	*M. musculus*	668	M	212-541	(168)
109940092	16572	P33175	Kif 5A	*M. musculus*	1027	N	7-327	(172)
118572490	110033	Q3V300	Kif 22	*M. musculus*	660	N	48-361	(118)
123284070	AL663079	CAM19254	novel protein	*M. musculus*	710	N	11-304	
124056472	16582	O35231	Kif C3	*M. musculus*	824	C	441-769	(173)
124487163	NM_001081177	NP_001074646	KIF13B	*M. musculus*	1843	N	4-360	(165)
126215733	109242	Q6NWW5	Kif 24	*M. musculus*	1356	M	217-539	(168)
156564374	NM_177052	NP_796026	KIF6	*M. musculus*	802	N	5-343	(165)
156616322	NM_ 001102615.1	NP_001096085	Kif19A	*M. musculus*	997	N	11-346	(174)
158564068		Q9QWT9	KIFC1	*M. musculus*	674	C	309-667	(157)
158931128	16563	P28740	Kif 2A	*M. musculus*	705	M	221-550	(155)
160013076	668303	Q52KG5	Kif 26A	*M. musculus*	1881	M	364-716	(174)
160013784	269152	Q7TNC6	Kif 26B	*M. musculus*	2112	M	450-798	(118)
189046786	70218	Q6PFD6	Kif 18B	*M. musculus*	834	N	18-353	(168)
254553479	NM_010626	NP_034756	KIF7	*M. musculus*	1347	N	14-350	(165)
1839174	NHU86521	AAB47851	kinesin	*N. haematococca*	929	N	4-329	(175)
119472836	XM_001258424	XP_001258425	kinesin family protein	*N. fischeri*	690	N	2-351	(55)
119473281	XM_001258550	XP_001258551	kinesin heavy chain subunit	*N. fischeri*	872	N	1-270	(55)
119478417	XM_001259364	XP_001259365	kinesin family protein	*N. fischeri*	1748	N	50-441	(55)
119478542	XM_001259385	XP_001259386	kinesin family protein	*N. fischeri*	655	N	92-449	(55)
119481813	XM_001260934	XP_001260935	kinesin family protein	*N. fischeri*	1619	N	7-356	(55)
119482528	XM_001261291	XP_001261292	kinesin family protein (KipA)	*N. fischeri*	955	M	214-570	(55)
119488650	XM_001262774	XP_001262775	kinesin family protein (BimC)	*N. fischeri*	1190	N	75-419	(55)
119498243	XM_001265878	XP_001265879	kinesin family protein	*N. fischeri*	1009	N	9-379	(55)

GI	Gene ID	NCBI Prot ID	Protein Name	Taxa	No. aa in full length protein	Motor type (N/M/C)	Motor domain	Ref.
6016437	3880594	P48467	KINH	*N. crassa*	928	N	5-330	(176)
1491931	NTU52078	AAC49393	kinesin-like protein	*N. tabacum*	1265	C	887-1213	(177)
11132775		O23826	K125	*N. tabacum*	1006	N	7-364	(178)
149409204	XM_001513504	XP_001513554	similar to Flj37300-A-prov	*O. anatinus*	1018	N	14-351	(179)
149409521	XM_001510950	XP_001511000	similar to HSPC111	*O. anatinus*	709	N	11-354	(179)
149413988	XM_001518095	XP_001518145	similar to KIF27A	*O. anatinus*	523	N	4-337	(179)
149481063	XM_001521357	XP_001521407	hypothetical protein	*O. anatinus*	763	M	247-562	(179)
149632045	XM_001513131	XP_001513181	similar to kinesin-like protein 2	*O. anatinus*	1424	N	53-391	(179)
149632525	XM_001508207	XP_001508257	similar to KIF13A	*O. anatinus*	2000	N	4-360	(179)
149632650	XM_001510925	XP_001510975	similar to KIF19	*O. anatinus*	985	N	71-404	(179)
149634753	XM_001508079	XP_001508129	similar to kinesin heavy chain	*O. anatinus*	965	N	6-325	(179)
149635339	XM_001506223	XP_001506273	similar to KIF2A	*O. anatinus*	959	M	366-695	(179)
149636611	XM_001509006	XP_001509056	hypothetical protein	*O. anatinus*	787	M	218-548	(179)
149638210	XM_001510628	XP_001510678	KIF3A	*O. anatinus*	767	N	38-370	(179)
149639091	XM_001509719	XP_001509769	similar to kinesin family member 1B	*O. anatinus*	1770	N	4-355	(179)
149640967	XM_001515302	XP_001515352	C20orf23	*O. anatinus*	1325	N	18-375	(179)
149641641	XM_001513203	XP_001513253	hypothetical protein	*O. anatinus*	1021	N	5-359	(179)
149641649	XM_001513391	XP_001513441	kinesin family member 26B	*O. anatinus*	1982	M	344-692	(179)
149642076	XM_001515263	XP_001515313	KIF24	*O. anatinus*	1317	M	184-506	(179)
75109919	4340046	Q5VQ09	Ark 2	*O. sativa*	891	N	95-398	(180)
108705832	DP000009	ABF93627	putative kinesin	*O. sativa*	622	N	1-274	(181)
108707695	DP000009	ABF95490	putative kinesin-4	*O. sativa*	1014	M	388-712	(181)
108712245	DP000009	ABG00040	putative kinesin-1	*O. sativa*	487	M	92-389	(181)
115438358	NM_001050055	NP_001043520	Os01g0605500	*O. sativa*	776	M	205-534	(182)
115450791	NM_001055531	NP_001048996	kinesin protein	*O. sativa*	993	N	115-445	(182)
115460740	NM_001060505	NP_001053970	Os04g0629700	*O. sativa*	788	C	430-770	(182)
115464949	NM_001062609	NP_001056074	Os05g0521300	*O. sativa*	926	M	389-721	(182)
115474523	NM_001067393	NP_001060858	Os08g0117000	*O. sativa*	549	N	82-329	(182)
115488960	NM_001073499	NP_001066967	Os12g0547500 protein	*O. sativa*	954	M	372-702	(182)
116310281	CR855188	CAH67300	OSIGBa0102D10.3	*O. sativa*	1154	M	119-437	(183)
116310358	CR855195	CAH67371	OSIGBa0128P10.5	*O. sativa*	979	M	346-664	(183)
125532696	CM000135	EAY79261	putative protein	*O. sativa*	622	C	226-560	(184)
125545868	CM000128	EAY92007	putative protein	*O. sativa*	783	N	14-349	(184)
125546553	CM000128	EAY92692	putative protein	*O. sativa*	440	N	92-426	(184)
125550139	CM000129	EAY95961	putative protein	*O. sativa*	1274	C	896-1222	(184)
125550610	CM000130	EAY96319	putative protein	*O. sativa*	1056	N	47-404	(184)
125553964	CM000131	EAY99569	putative protein	*O. sativa*	891	N	95-398	(184)
125560717	CM000133	EAZ06165	putative protein	*O. sativa*	549	N	74-333	(184)
125560719	CM000133	EAZ06167	putative protein	*O. sativa*	604	N	61-388	(184)
125569796	CM000138	EAZ11311	putative protein	*O. sativa*	793	N	93-420	(184)
125575383	CM000147	EAZ16667	putative protein	*O. sativa*	1071	M	114-415	(184)
206557943	4331654	Q0DV28	Ark 1	*O. sativa*	945	N	57-397	(181)
145345569	XM_001417241	XP_001417278	predicted protein	*O. 'lucimarinus'*	493	C	110-433	(185)
145345868	XM_001417384	XP_001417421	predicted protein	*O. 'lucimarinus'*	781	N	18-376	(185)
145348596	XM_001418695	XP_001418732	predicted protein	*O. 'lucimarinus'*	384	N	44-373	(185)
145351435	XM_001420047	XP_001420084	predicted protein	*O. 'lucimarinus'*	393	N	52-383	(185)

GI	Gene ID	NCBI Prot ID	Protein Name	Taxa	No. aa in full length protein	Motor type (N/M/C)	Motor domain	Ref.
145351791	XM_001420209	XP_001420246	predicted protein	*O. 'lucimarinus'*	1109	C	733-1061	(185)
116057025	CR954204	CAL51452	kinesin	*O. tauri*	771	N	8-370	(186)
116057299	CR954204	CAL51726	kinesin-like protein B	*O. tauri*	2739	C	2379-2689	(186)
116057438	CR954204	CAL51865	kinesin protein	*O. tauri*	689	N	93-426	(186)
116057919	CR954205	CAL54122	kinesin-like protein	*O. tauri*	500	N	43-368	(186)
116058861	CR954207	CAL54568	kinesin	*O. tauri*	979	N	54-378	(186)
116059118	CR954208	CAL54825	kinesin-like protein KRP180	*O. tauri*	1234	N	8-382	(186)
116059314	CR954208	CAL55021	Kinesin	*O. tauri*	1310	C	904-1231	(186)
116059965	CR954210	CAL56024	OSJNBa0089N06.17	*O. tauri*	725	C	384-714	(186)
116060309	CR954211	CAL55645	kinesin-like protein	*O. tauri*	1220	C	841-1169	(186)
116060488	CR954211	CAL55824	putative kinesin	*O. tauri*	790	N	37-424	(186)
114595517	XM_001170275	XP_001170275		*P. troglodytes*	2701	N	6-329	(187)
114605632	XM_001170727	XP_001170727	Kif 13A	*P. troglodytes*	1749	N	4-359	(187)
114626328	XM_520205	XP_520205		*P. troglodytes*	620	N	34-332	(187)
114644157	XM_509167	XP_509167		*P. troglodytes*	1028	N	14-323	(187)
114656598	XM_001144280	XP_001144280		*P. troglodytes*	848	N	2-391	(187)
114681460	XM_001155482	XP_001155482	Kif 3B	*P. troglodytes*	747	N	18-340	(187)
12055369	AJ293506	CAC20783	kinesin-like boursin	*P. lividus*	1081	N	10-360	(188)
145478285	XM_001425128	XP_001425165	Chromosome scaffold_107	*P. tetraurelia*	773	N	6-328	(189)
145478411	XM_001425191	XP_001425228	Chromosome scaffold_107	*P. tetraurelia*	1008	N	14-347	(189)
145479171	XM_001425571	XP_001425608	Chromosome scaffold_109	*P. tetraurelia*	731	N	7-346	(189)
145480021	XM_001425996	XP_001426033	Chromosome scaffold_11	*P. tetraurelia*	721	N	6-337	(189)
145480537	XM_001426254	XP_001426291	Chromosome scaffold_111	*P. tetraurelia*	773	N	3-354	(189)
145480545	XM_001426258	XP_001426295	Chromosome scaffold_111	*P. tetraurelia*	767	N	7-342	(189)
145480699	XM_001426335	XP_001426372	Chromosome scaffold_111	*P. tetraurelia*	641	N	52-383	(189)
145481219	XM_001426595	XP_001426632	Chromosome scaffold_113	*P. tetraurelia*	787	N	4-326	(189)
145481461	XM_001426716	XP_001426753	Chromosome scaffold_114	*P. tetraurelia*	782	N	11-335	(189)
145481667	XM_001426819	XP_001426856	Chromosome scaffold_114	*P. tetraurelia*	704	N	22-324	(189)
145481901	XM_001426936	XP_001426973	Chromosome scaffold_115	*P. tetraurelia*	798	N	4-334	(189)
145482705	XM_001427338	XP_001427375	Chromosome scaffold_118	*P. tetraurelia*	654	N	48-371	(189)
145482763	XM_001427367	XP_001427404	Chromosome scaffold_118	*P. tetraurelia*	761	N	4-334	(189)
145482939	XM_001427455	XP_001427492	Chromosome scaffold_118	*P. tetraurelia*	544	N	4-281	(189)
145482983	XM_001427477	XP_001427514	Chromosome scaffold_118	*P. tetraurelia*	800	N	21-361	(189)
145483965	XM_001427968	XP_001428005	Chromosome scaffold_12	*P. tetraurelia*	775	N	12-361	(189)
145484348	XM_001428147	XP_001428184	Chromosome scaffold_120	*P. tetraurelia*	800	N	4-334	(189)
145484858	XM_001428401	XP_001428438	Chromosome scaffold_122	*P. tetraurelia*	798	N	11-337	(189)
145485032	XM_001428488	XP_001428525	Chromosome scaffold_122	*P. tetraurelia*	710	N	22-332	(189)
145485173	XM_001428558	XP_001428595	Chromosome scaffold_123	*P. tetraurelia*	496	C	186-494	(189)

GI	Gene ID	NCBI Prot ID	Protein Name	Taxa	No. aa in full length protein	Motor type (N/M/C)	Motor domain	Ref.
145485614	XM_001428778	XP_001428815	Chromosome scaffold_124	*P. tetraurelia*	601	N	16-355	(189)
145485949	XM_001428945	XP_001428982	Chromosome scaffold_125	*P. tetraurelia*	643	N	4-326	(189)
145486104	XM_001429022	XP_001429059	Chromosome scaffold_126	*P. tetraurelia*	776	N	3-373	(189)
145486638	XM_001429288	XP_001429325	Chromosome scaffold_128	*P. tetraurelia*	794	N	6-336	(189)
145486720	XM_001429329	XP_001429366	Chromosome scaffold_128	*P. tetraurelia*	736	N	5-337	(189)
145486941	XM_001429439	XP_001429476	Chromosome scaffold_129	*P. tetraurelia*	747	N	2-291	(189)
145495173	XM_001433543	XP_001433580	Chromosome scaffold_150	*P. tetraurelia*	714	N	21-253	(189)
145495671	XM_001433791	XP_001433828	Chromosome scaffold_152	*P. tetraurelia*	965	N	3-348	(189)
145498610	XM_001435255	XP_001435292	Chromosome scaffold_162	*P. tetraurelia*	704	N	1-313	(189)
145499231	XM_001435564	XP_001435601	Chromosome scaffold_165	*P. tetraurelia*	558	N	20-305	(189)
145499886	XM_001435890	XP_001435927	Chromosome scaffold_168	*P. tetraurelia*	558	N	3-312	(189)
145503258	XM_001437569	XP_001437606	Chromosome scaffold_180	*P. tetraurelia*	802	N	41-387	(189)
145507993	XM_001439909	XP_001439946	Chromosome scaffold_23	*P. tetraurelia*	509	N	1-336	(189)
145512407	XM_001442083	XP_001442120	Chromosome scaffold_29	*P. tetraurelia*	616	N	18-356	(189)
145513366	XM_001442557	XP_001442594	Chromosome scaffold_3	*P. tetraurelia*	785	N	13-361	(189)
145513390	XM_001442569	XP_001442606	Chromosome scaffold_3	*P. tetraurelia*	1351	N	10-333	(189)
145514045	XM_001442896	XP_001442933	Chromosome scaffold_30	*P. tetraurelia*	772	N	29-375	(189)
145514776	XM_001443256	XP_001443293	Chromosome scaffold_31	*P. tetraurelia*	783	N	29-382	(189)
145515403	XM_001443564	XP_001443601	Chromosome scaffold_32	*P. tetraurelia*	1837	N	9-324	(189)
145516579	XM_001444141	XP_001444178	Chromosome scaffold_34	*P. tetraurelia*	707	N	3-310	(189)
145516709	XM_001444206	XP_001444243	Chromosome scaffold_34	*P. tetraurelia*	632	N	6-328	(189)
145517013	XM_001444358	XP_001444395	Chromosome scaffold_34	*P. tetraurelia*	635	N	6-314	(189)
145517416	XM_001444554	XP_001444591	Chromosome scaffold_35	*P. tetraurelia*	857	M	155-467	(189)
145520032	XM_001445840	XP_001445877	Chromosome scaffold_39	*P. tetraurelia*	508	N	6-342	(189)
145523511	XM_001447557	XP_001447594	Chromosome scaffold_43	*P. tetraurelia*	983	N	24-349	(189)
145523762	XM_001447677	XP_001447714	Chromosome scaffold_44	*P. tetraurelia*	1033	N	37-360	(189)
145524479	XM_001448030	XP_001448067	Chromosome scaffold_45	*P. tetraurelia*	1403	N	15-343	(189)
145526014	XM_001448781	XP_001448818	Chromosome scaffold_48	*P. tetraurelia*	638	N	6-310	(189)
145530273	XM_001450877	XP_001450914	Chromosome scaffold_54	*P. tetraurelia*	1892	N	11-334	(189)
145530291	XM_001450886	XP_001450923	Chromosome scaffold_54	*P. tetraurelia*	633	N	81-364	(189)
145531577	XM_001451518	XP_001451555	Chromosome scaffold_56	*P. tetraurelia*	522	C	210-490	(189)
145532563	XM_001452000	XP_001452037	Chromosome scaffold_58	*P. tetraurelia*	784	M	295-610	(189)
145534436	XM_001452925	XP_001452962	Chromosome scaffold_60	*P. tetraurelia*	995	N	4-312	(189)

GI	Gene ID	NCBI Prot ID	Protein Name	Taxa	No. aa in full length protein	Motor type (N/M/C)	Motor domain	Ref.
145535285	XM_001453344	XP_001453381	Chromosome scaffold_61	*P. tetraurelia*	707	N	3-310	(189)
145536476	XM_001453923	XP_001453960	Chromosome scaffold_64	*P. tetraurelia*	553	N	5-337	(189)
145537201	XM_001454280	XP_001454317	Chromosome scaffold_65	*P. tetraurelia*	489	N	4-319	(189)
145538163	XM_001454750	XP_001454787	Chromosome scaffold_67	*P. tetraurelia*	980	N	2-329	(189)
145539930	XM_001455618	XP_001455655	Chromosome scaffold_7	*P. tetraurelia*	1366	N	10-340	(189)
145540285	XM_001455795	XP_001455832	Chromosome scaffold_70	*P. tetraurelia*	727	N	5-342	(189)
145540391	XM_001455848	XP_001455885	Chromosome scaffold_70	*P. tetraurelia*	686	N	35-301	(189)
145540395	XM_001455850	XP_001455887	Chromosome scaffold_70	*P. tetraurelia*	1138	N	4-365	(189)
145540613	XM_001455959	XP_001455996	Chromosome scaffold_70	*P. tetraurelia*	818	N	25-363	(189)
145541287	XM_001456295	XP_001456332	Chromosome scaffold_72	*P. tetraurelia*	517	N	6-342	(189)
145541301	XM_001456302	XP_001456339	Chromosome scaffold_72	*P. tetraurelia*	1798	N	39-354	(189)
145541319	XM_001456311	XP_001456348	Chromosome scaffold_72	*P. tetraurelia*	631	N	81-364	(189)
145541646	XM_001456474	XP_001456511	Chromosome scaffold_73	*P. tetraurelia*	1127	N	1-361	(189)
145542949	XM_001457124	XP_001457161	Chromosome scaffold_76	*P. tetraurelia*	980	N	3-320	(189)
145543304	XM_001457301	XP_001457338	Chromosome undetermined scaffold_77	*P. tetraurelia*	594	N	6-340	(189)
145543765	XM_001457531	XP_001457568	Chromosome scaffold_78	*P. tetraurelia*	1117	N	4-344	(189)
145544030	XM_001457663	XP_001457700	Chromosome scaffold_79	*P. tetraurelia*	563	N	5-346	(189)
145544264	XM_001457780	XP_001457817	Chromosome scaffold_79	*P. tetraurelia*	980	N	3-320	(189)
145545237	XM_001458266	XP_001458303	Chromosome scaffold_8	*P. tetraurelia*	733	N	4-320	(189)
145545598	XM_001458446	XP_001458483	Chromosome scaffold_80	*P. tetraurelia*	711	N	3-355	(189)
145546400	XM_001458846	XP_001458883	Chromosome scaffold_82	*P. tetraurelia*	507	N	6-307	(189)
145549630	XM_001460457	XP_001460494	Chromosome scaffold_9	*P. tetraurelia*	490	N	4-319	(189)
145550413	XM_001460848	XP_001460885	Chromosome scaffold_90	*P. tetraurelia*	592	N	6-340	(189)
145551081	XM_001461181	XP_001461218	Chromosome scaffold_92	*P. tetraurelia*	587	N	18-356	(189)
145551276	XM_001461278	XP_001461315	Chromosome scaffold_93	*P. tetraurelia*	812	N	21-368	(189)
145551434	XM_001461357	XP_001461394	Chromosome scaffold_93	*P. tetraurelia*	1076	N	3-357	(189)
145552523	XM_001461900	XP_001461937	Chromosome scaffold_96	*P. tetraurelia*	660	N	6-329	(189)
145553128	XM_001462202	XP_001462239	Chromosome scaffold_97	*P. tetraurelia*	758	N	38-356	(189)
169594924	XM_001790834	XP_001790886	putative protein	*P. nodorum*	1022	N	8-403	(190)
169595346	XM_001791045	XP_001791097	putative protein	*P. nodorum*	1168	N	57-403	(190)
169600633	XM_001793687	XP_001793739	putative protein	*P. nodorum*	745	N	2-331	(190)
169601428	XM_001794084	XP_001794136	putative protein	*P. nodorum*	728	M	17-374	(190)
169606994	XM_001796865	XP_001796917	putative protein	*P. nodorum*	954	N	16-333	(190)
169608075	XM_001797405	XP_001797457	putative protein	*P. nodorum*	1660	N	52-436	(190)
169614451	XM_001800590	XP_001800642	putative protein	*P. nodorum*	558	N	9-356	(190)
169625889	XM_001806295	XP_001806347	putative protein	*P. nodorum*	450	M	181-436	(190)
190345690	CH408156	EDK37617	putative protein	*P. guilliermondii*	531	N	28-358	(146)

GI	Gene ID	NCBI Prot ID	Protein Name	Taxa	No. aa in full length protein	Motor type (N/M/C)	Motor domain	Ref.
190345935	CH408156	EDK37907	putative protein	*P. guilliermondii*	770	N	9-376	(146)
190347243	CH408158	EDK39482	putative protein	*P. guilliermondii*	666	M	117-440	(146)
190348916	CH408161	EDK41470	putative protein	*P. guilliermondii*	871	N	1-369	(146)
150863921	XM_001382530	XP_001382567	predicted protein	*P. stipitis*	749	N	52-379	(191)
150863948	XM_001382566	XP_001382603	predicted protein	*P. stipitis*	912	N	22-402	(191)
150864534	XM_001383349	XP_001383386	predicted protein	*P. stipitis*	532	N	7-387	(191)
150866075	XM_001385514	XP_001385551	predicted protein	*P. stipitis*	907	N	1-383	(191)
124505045	XM_001351228	XP_001351264	putative kinesin	*P. falciparum*	1200	M	258-587	(192)
156094934	XM_001613453	XP_001613503	putative kinesin	*P. vivax*	794	M	74-395	(193)
156094979	XM_001613475	XP_001613525	putative kinesin	*P. vivax*	1490	M	797-1132	(193)
156095883	XM_001613926	XP_001613976	putative kinesin	*P. vivax*	1103	N	42-370	(193)
156096971	XM_001614469	XP_001614519	putative kinesin	*P. vivax*	1285	M	508-838	(193)
156099151	XM_001615528	XP_001615578	kinesin protein	*P. vivax*	794	N	4-364	(193)
156101780	XM_001616533	XP_001616583	putative kinesin	*P. vivax*	1677	N	26-422	(193)
75041752	100172845	Q5R9K7	Kif 5A	*P. abelii*	1032	N	7-327	(194)
75061599	100173987	Q5R4H3	Kif 3A	*P. abelii*	702	N	13-245	(194)
75061716	100169734	Q5R706	Kif 3C	*P. abelii*	793	N	9-365	(194)
75061830	100174485	Q5R9Y9	Kif 2A	*P. abelii*	744	M	222-551	(194)
75062021	100189644	Q5REP4	Kif 22	*P. abelii*	665	N	60-366	(194)
90655955	DQ444242	ABD96574	MCAK	*P. tridactylus*	728	M	258-587	(195)
3913949	85248	O55165	Kif 3C	*R. norvegicus*	796	N	9-367	(196)
12230206	113886	O35787	Kif 1C	*R. norvegicus*	1097	N	4-354	(197)
19343354	AF035952	AAB88700	KRP3A	*R. norvegicus*	486	N	5-343	(198)
81883705	294286	Q5XI63	Kif C1	*R. norvegicus*	693	C	328-686	(118)
81889019	293502	Q5I0E8	Kif 22	*R. norvegicus*	657	N	48-358	(118)
81892770	314906	Q6QLM7	Kif 5A	*R. norvegicus*	1027	N	7-327	(133)
81894343	246209	Q7M6Z5	Kif 27	*R. norvegicus*	1394	N	4-342	(199)
81894691	353302	Q7TSP2	Kif 15	*R. norvegicus*	1385	N	25-363	(170)
84028216	117548	O88658	Kif 1B	*R. norvegicus*	1816	N	4-361	(200)
109892476	117550	Q2PQA9	KINH	*R. norvegicus*	963	N	6-325	(133)
123785745	303575	Q4KLL9	Kif 18B	*R. norvegicus*	826	N	18-353	(118)
157153636	NM_001103352	NP_001096822	putative protein	*R. norvegicus*	753	C	370-698	(198)
157819777		NP_001101200	Kif 5C	*R. norvegicus*	955	N	6-327	(201)
158705884		Q5XI51	Kif 2B	*R. norvegicus*	664	M	212-541	(199)
158705885		Q9WV63	Kif 2A	*R. norvegicus*	705	M	221-550	(199)
294862506	171529	KIF2C_RAT (Q62909)	Kif2C	*R. norvegicus*	671	M	203-532	(198)
125417	852216	P28742	Kip 1	*S. cerevisiae*	1111	N	50-418	(202)
125418	855948	P28743	Kip 2	*S. cerevisiae*	706	M	102-493	(202)
417786	853783	P32364	SMY 1	*S. cerevisiae*	656	N	27-362	(203)
1723958	852655	P53086	Kip 3	*S. cerevisiae*	805	N	84-438	(204)
126417154	EF441211	ABO13867	Kif C1	*S. salar*	625	C	280-618	(205)
1706200	2542732	P24339	Cut 7	*S. pombe*	1085	N	70-429	(206)
3219911	2540361	O14343	Klp 5	*S. pombe*	883	N	6-390	(207)
15214062	2542195	Q9US60	Klp 3	*S. pombe*	554	N	1-325	(208)
26395879	2539700	O59751	Klp 6	*S. pombe*	784	N	6-389	(207)
51316544	2542849	Q9UTL2	Klp 8	*S. pombe*	511	N	5-354	(209)
118573057	2539857	Q1MTQ1	Tea 2	*S. pombe*	628	M	132-460	(210)
212288569		Q1MTQ7	Yog 1	*S. pombe*	633	N	2-390	(209)
156035543	XM_001585833	XP_001585883	putative protein	*S. sclerotiorum*	1174	N	82-432	(65)
156039912	XM_001587013	XP_001587063	putative protein	*S. sclerotiorum*	712	N	7-339	(65)
156042468	XM_001587741	XP_001587791	putative protein	*S. sclerotiorum*	735	N	2-331	(65)
156055018	XM_001593383	XP_001593433	putative protein	*S. sclerotiorum*	747	N	7-372	(65)
156058135	XM_001594941	XP_001594991	putative protein	*S. sclerotiorum*	987	M	217-574	(65)
156060675	XM_001596210	XP_001596260	putative protein	*S. sclerotiorum*	1712	N	29-416	(65)
156061551	XM_001596648	XP_001596698	kinesin heavy chain	*S. sclerotiorum*	929	N	2-323	(65)
156064583	XM_001598163	XP_001598213	putative protein	*S. sclerotiorum*	596	N	54-402	(65)
1369852	POTKHCL	AAB37756	kinesin heavy chain-like protein	*S. tuberosum*	1265	C	887-1213	(211)
547774	373178	P35978	KINH	*S. purpuratus*	1031	N	6-325	(212)
1170652	373530	P46871	Krp 95	*S. purpuratus*	742	N	7-339	(213)
1170653	373466	P46872	Krp 85	*S. purpuratus*	699	N	9-335	(213)
74942707	373236	Q9GYZ0	Kif 15	*S. purpuratus*	1463	N	17-354	(214)
6016438		O43093	KINH	*S. racemosum*	935	N	4-329	(215)

GI	Gene ID	NCBI Prot ID	Protein Name	Taxa	No. aa in full length protein	Motor type (N/M/C)	Motor domain	Ref.
5640166	AJ244020	CAB51556	kinesin-II homologue Q9U0D5	*T. thermophila*	735	N	12-341	(216)
5679735	AJ244021	CAB51811	kinesin-II homologue Q9U921	*T. thermophila*	697	N	9-338	(216)
118394633	XM_001029681	XP_001029681	kinesin protein	*T. thermophila*	769	N	78-427	(217)
118395509	XM_001030103	XP_001030103	kinesin protein	*T. thermophila*	2114	N	93-496	(217)
146162423	XM_001471116	XP_001471166	putative protein	*T. thermophila*	431	N	40-357	(217)
146179605	XM_001470861	XP_001470911	kinesin heavy chain	*T. thermophila*	721	N	61-400	(217)
123388204	XM_001299535	XP_001299536	kinesin protein	*T. vaginalis*	511	N	37-356	(218)
123396914	XM_001300991	XP_001300992	kinesin protein	*T. vaginalis*	659	N	7-330	(218)
123411586	XM_001303909	XP_001303910	kinesin protein	*T. vaginalis*	584	N	44-377	(218)
123421229	XM_001305934	XP_001305935	kinesin protein	*T. vaginalis*	670	N	4-303	(218)
123448844	XM_001313146	XP_001313147	kinesin protein	*T. vaginalis*	657	N	4-319	(218)
123455652	XM_001315533	XP_001315568	kinesin protein	*T. vaginalis*	651	N	5-328	(218)
123473438	XM_001319872	XP_001319907	kinesin protein	*T. vaginalis*	679	N	10-337	(218)
123474972	XM_001320631	XP_001320666	kinesin protein	*T. vaginalis*	657	N	5-318	(218)
123479831	XM_001323037	XP_001323072	kinesin protein	*T. vaginalis*	647	M	103-436	(218)
123490274	XM_001325546	XP_001325581	kinesin protein	*T. vaginalis*	660	N	6-336	(218)
123492570	XM_001326059	XP_001326094	kinesin protein	*T. vaginalis*	487	N	57-380	(218)
123497284	XM_001327116	XP_001327151	kinesin protein	*T. vaginalis*	566	N	57-379	(218)
123505760	XM_001329017	XP_001329052	kinesin protein	*T. vaginalis*	659	N	28-352	(218)
123507578	XM_001329412	XP_001329447	kinesin protein	*T. vaginalis*	696	N	9-335	(218)
123974990	XM_001314085	XP_001314093	kinesin protein	*T. vaginalis*	754	N	99-431	(218)
123976204	XM_001330775	XP_001330811	kinesin protein	*T. vaginalis*	726	N	4-305	(218)
123976709	XM_001314546	XP_001314575	kinesin protein	*T. vaginalis*	469	N	44-377	(218)
124001101	XM_001276970	XP_001276971	kinesin protein	*T. vaginalis*	678	N	8-338	(218)
154413434	XM_001579697	XP_001579747	kinesin protein	*T. vaginalis*	674	N	9-335	(218)
11245496	AF319546	AAG33641	Kif C1	*T. brucei*	820	C	487-814	(219)
2062750	UMU92844	AAB63336	kinesin protein	*U. maydis*	1459	M	2-333	(220)
2062752	UMU92845	AAB63337	kinesin protein	*U. maydis*	968	N	2-333	(220)
147774373	AM461173	CAN72398	putative protein	*V. vinifera*	1824	M	994-1292	(221)
147783402	AM479280	CAN75214	putative protein	*V. vinifera*	1219	N	92-412	(221)
147791474	AM458293	CAN74504	putative protein	*V. vinifera*	1058	M	422-761	(221)
147798215	AM434210	CAN60539	putative protein	*V. vinifera*	726	M	101-416	(221)
147815114	AM459464	CAN65659	putative protein	*V. vinifera*	1742	M	472-787	(221)
147818972	AM434964	CAN67116	putative protein	*V. vinifera*	1817	N	53-384	(221)
147838326	AM455602	CAN61255	putative protein	*V. vinifera*	554	N	80-416	(221)
147856290	AM480980	CAN79643	putative protein	*V. vinifera*	881	N	7-366	(221)
147864402	AM437988	CAN80502	putative protein	*V. vinifera*	1082	M	105-403	(221)
147866344	AM488021	CAN81981	putative protein	*V. vinifera*	1239	M	283-566	(221)
147867330	AM460760	CAN81188	putative protein	*V. vinifera*	960	M	108-403	(221)
119217	397908	P28025	Eg5 1	*X. laevis*	1060	N	9-361	(222)
3023586	394322	P79955	Ctk 2	*X. laevis*	643	C	292-636	(223)
3550684	AJ009839	CAA08879	kinesin like protein 3	*X. laevis*	744	N	16-340	(224)
13235654	AJ311602	CAC33801	kinesin-like protein	*X. laevis*	699	N	13-345	(225)
13959688	380006	Q91636	Kif 2C	*X. laevis*	730	M	261-590	(226)
13959700	379622	Q91637	Kif 2A	*X. laevis*	682	M	197-526	(226)
15209365	AJ297516	CAC14732	kinesin-like protein	*X. laevis*	1499	N	2-385	(227)
18202613	394332	Q91784	Kif 4	*X. laevis*	1226	N	7-338	(228)
82209779	373792	Q7ZXX2	Kif 19	*X. laevis*	997	N	11-346	(229)
82217541	397909	Q91785	Kif 15A	*X. laevis*	1388	N	25-364	(230)
82221703		Q9I869	Kif 22A	*X. laevis*	651	N	31-357	(231)
110468094	DQ680042	ABG74914	kinesin heavy chain	*X. laevis*	962	N	6-325	(232)
123910885	443568	Q498L9	Kif 15B	*X. laevis*	1387	N	25-364	(230)
147900710	NM_001087485	NP_001080954	kinesin-related protein	*X. laevis*	2954	N	6-330	(233)
205815111		Q7ZYL5	Kif 22B	*X. laevis*	650	N	31-357	(234)

GI	Gene ID	NCBI Prot ID	Protein Name	Taxa	No. aa in full length protein	Motor type (N/M/C)	Motor domain	Ref.
82202386	394855	Q6P3R1	Kif 22	*X. tropicalis*	639	N	25-343	(229)
156717212	NM_001016245	NP_001016245	putative protein	*X. tropicalis*	947	N	20-355	(229)
156717352	NM_001102745	NP_001096215	putative protein	*X. tropicalis*	1033	N	9-329	(229)

A SATé

```
        β3   P-loop
K1   GTIFAYGQTSSGKTHT
K2   GTIFAYGQTGTGKTFT
K3   ACIFAYGQTGSGKSYT
K4   ATVLAYGQTGSGKTYT
K5   CTIFAYGQTGTGKTFT
K6   SLLFTYGVTGSGKTYT
K7   GTIFAYGQTSSGKTYT
K8   CTVFAYGATGSGKTYT
K10  ANVIAHGARNSGKTHL
K13  ATCFAYGQTGSGKTHT
K14  VGLIAYGQTGSGKTHT
K15  SSVFAYGQTGSGKTYT
```

```
          β1a         β1b                              β2
K1   DKY--IAK--FQG---EDT--VV--------------------IA-SK-P
K2   HKI--AVT--CNDEE-KAV--NIK-------------SLSQE-DP-PR-T
K3   TKC--VVD--VDA---NKV--ILNPVNTNL--S--K--GDAR-GQ-PK-C
K4   CTD--CIT--VAP---KEP--QVH-----------------I-G--SH-T
K5   AHS--IVE--CDPVR-KEV--SVR-----------TGGLADK-SS-RK-T
K6   D---------------EGS--IQTVLPP----AQFR-RENAP-QV-EK-V
K7   -----LWQ--VKE---GRS--IQ----------------LAD-SH-AE-P
K8   -EN--IVS--IFN---KNY--VLIEKENEK--ECYL--LSQK-KK-QS-T
K10  ISV--QKP--MGD---DSE--TVT--------ISFG--AQFA-GS-KD-S
K13  NLD--IIT--VPSA--DSL--IVHELRLKV-----D--LTKF-LE-HH-K
K14  SSA--IEY--PAI---DTI--RIN-----------E----GS-KP-GI-V
K15  GDM--IVE--KMS---KDS--LT--------------------VS-GQ-T
```

```
       switch I        β6
K1   NEHSSR-SHSIFLIN-----VKQE---N
K2   NLESSR-SHAMFTVT-----IESC---R
K3   NEESSR-SHAVLKIT-----LTHT---L
K4   NSQSSR-SHAIFTIT-----LEQK---K
K5   NAYSSR-SHSVFSVT-----IHMK---E
K6   NKDSSR-SHSVFTIK-----LVMA---P
K7   NERSSR-SHAIFRII-----IESR---K
K8   NKVSSR-SHAILQIY-----VYNE---I
K10  -DLPTR-SHKGVMIH-----VTTG---N
K13  NAKSSR-SHAVFQIA-----LHFP---D
K14  NESSSR-SHAVYMWK-----ITAH---Q
K15  NTESSR-SHCVFTCV-----VESR---C
```

```
          α4                L12
K1   GNVIS--ALA-E---G---S---------------
K2   GNVIS--SLV-DG--K---S-T-------------
K3   GLVIS--ALA-D---Q---S-A----GK----NKN
K4   GNVIS--ALG-D---E---K-K---------RKEG
K5   GRVIT--ALV-E---R---T-P-------------
K6   RQCIE--VLR-R---N---Q-K---------SSSQ
K7   RNLVK--SLS-E---SVDSK---------------
K8   ANCIN--SLA-S---N---R-N----------ISK
K10  QNVMY--ALN-A---N---E-S-------------
K13  KECIR--ALS-R---Q---S-S-------------
K14  QMCIS--QQR-S---Q---K-G-------------
K15  GNLIN--ILA-E---I---S-Q---------TGKP
```

B Clustal X

```
        β3   P-loop
K1   GTIFAYGQTSSGKTHT
K2   GTIFAYGQTGTGKTFT
K3   ACIFAYGQTGSGKSYT
K4   ATVLAYGQTGSGKTYT
K5   CTIFAYGQTGTGKTFT
K6   SLLFTYGVTGSGKTYT
K7   GTIFAYGQTSSGKTYT
K8   CTVFAYGATGSGKTYT
K10  ANVIAHGARNSGKTHL
K13  ATCFAYGQTGSGKTHT
K14  VGLIAYGQTGSGKTHT
K15  SSVFAYGQTGSGKTYT
```

```
         β1a      β1a    β2
K1   DKYIAKFQG--EDTVVIA-SKP
K2   GHKIAVTCNDEEKAVNIKSLSQ
K3   HTKCVVDVDANKVILNPVNTNL
K4   CTDCIT-VAPKEPQVHIGS---
K5   SAHSIVECDPVRKEV-----SV
K6   GSTPSLIAIDEGSIQTVLPPA-
K7   LWQVKEGRSIQLADSHAEP---
K8   --EENIVSIFNKNYVLIEK---
K10  SISVQKPMGDDSETVTISFGAQ
K13  KNLDIITVPSADSLIVHELR--
K14  ASSSAIEYPAIDTIRINE----
K15  IVE---------KMS-------
```

```
       switch I   β6
K1   NEHSSRSHSIFLINVKQE
K2   NLESSRSHAMFTVTIESC
K3   NEESSRSHAVLKITLTHT
K4   NSQSSRSHAIFTITLEQK
K5   NAYSSRSHSVFSVTIHMK
K6   NKDSSRSHSVFTIKLVMA
K7   NERSSRSHAIFR----II
K8   NKVSSRSHAILQ----IY
K10  SDLPTRSHKGVMIHVTTG
K13  NAKSSRSHAVFQ----IA
K14  NESSSRSHAVYMWKITAH
K15  NTESSRSHCVFTCVVESR
```

```
        α4      L12
K1   GNVISALAEGS-----
K2   GNVISSLVDGKS----
K3   GLVISALADQSA----
K4   GNVISALGDEKKRKE-
K5   GRVITALVERTP----
K6   RQCIEVLRRNQKSSSQ
K7   RNLVKSLSESV-----
K8   ANCINSLASNRN----
K10  QNVMYALNANES----
K13  KECIRALSRQ------
K14  QMCISQQRS-------
K15  GNLINILAEISQT---
```

Figure S1. SATé produces better resolution in sequence alignments over Clustal. Portions of the full kinesin motor domain sequence alignment calculated with (A) SATé and (B) Clustal are shown as examples. A single sequence is a representative from each of the indicated kinesin families. Secondary structures are indicated at the top of each portion of the alignment. Gaps correspond to indels in the full sequence alignment.

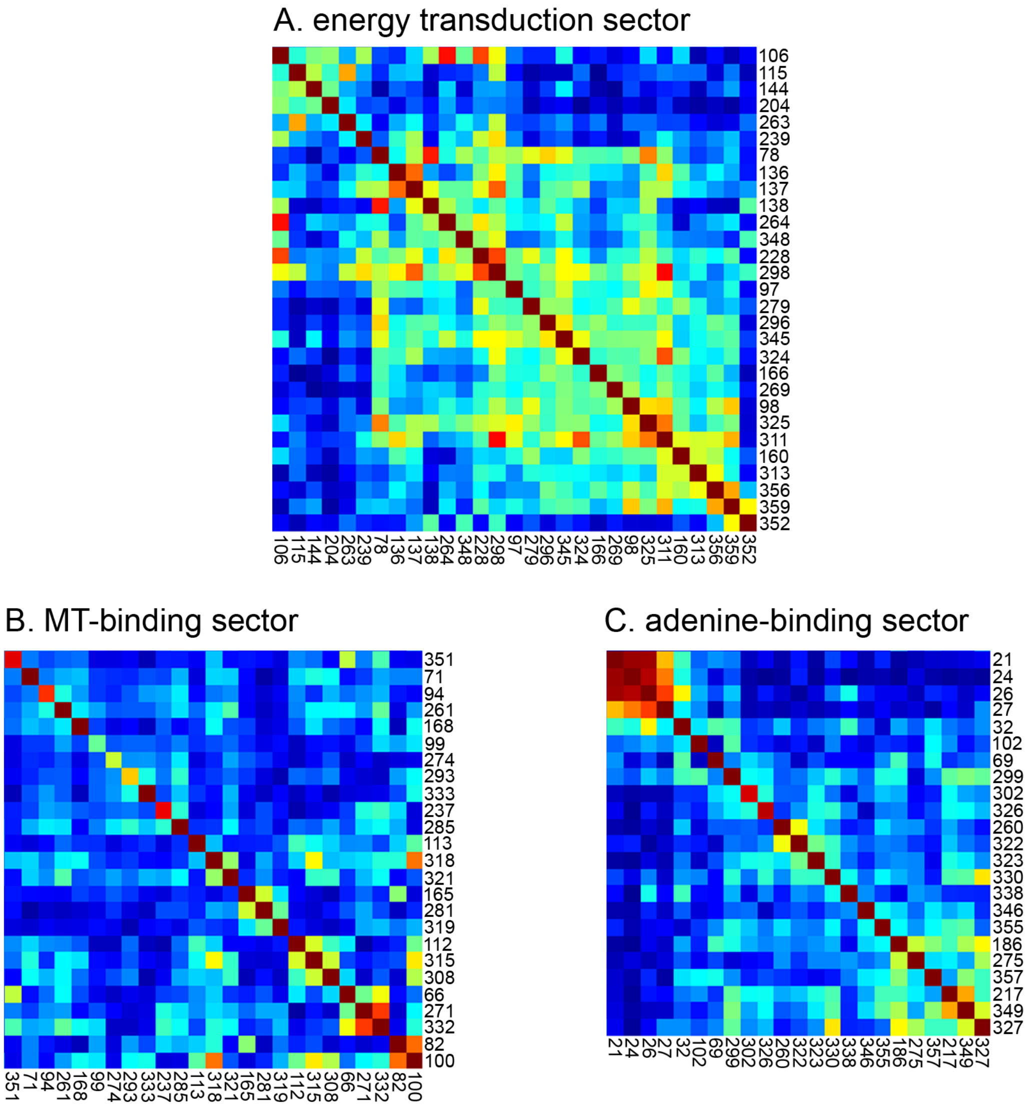

Figure S2. Matrices for the energy transduction, MT-binding, and adenine-binding sectors. An enlarged view of the matrix in Fig 2 that corresponds to the (A) energy transduction, (B), MT-binding, and (C) adenine-binding sectors is provided so that the individual residues comprising each sector can be identified. The residues listed are from human Kinesin-5 as in column 1 of Table 1.

REFERENCES FOR SUPPLEMENTARY INFORMATION

1. Lockless, S. W., and Ranganathan, R. (1999) Evolutionarily conserved pathways of energetic connectivity in protein families. *Science* **286**, 295-299
2. Suel, G. M., Lockless, S. W., Wall, M. A., and Ranganathan, R. (2003) Evolutionarily conserved networks of residues mediate allosteric communication in proteins. *Nat Struct Biol* **10**, 59-69
3. Hatley, M. E., Lockless, S. W., Gibson, S. K., Gilman, A. G., and Ranganathan, R. (2003) Allosteric determinants in guanine nucleotide-binding proteins. *Proc Natl Acad Sci U.S.A.* **100**, 14445-14450
4. Shulman, A. I., Larson, C., Mangelsdorf, D. J., and Ranganathan, R. (2004) Structural determinants of allosteric ligand activation in RXR heterodimers. *Cell* **116**, 417-429
5. Halabi, N., Rivoire, O., Leibler, S., and Ranganathan, R. (2009) Protein sectors: evolutionary units of three-dimensional structure. *Cell* **138**, 774-786
6. Smock, R. G., Rivoire, O., Russ, W. P., Swain, J. F., Leibler, S., Ranganathan, R., and Gierasch, L. M. (2010) An interdomain sector mediating allostery in Hsp70 molecular chaperones. *Mol Syst Biol* **6**, 414
7. Reynolds, K. A., McLaughlin, R. N., and Ranganathan, R. (2011) Hot spots for allosteric regulation on protein surfaces. *Cell* **147**, 1564-1575
8. Wang, J., Zhao, Y., Wang, Y., and Huang, J. (2013) Molecular dynamics simulations and statistical coupling analysis reveal functional coevolution network of oncogenic mutations in the CDKN2A-CDK6 complex. *FEBS Lett* **587**, 136-141
9. Russ, W. P., Lowery, D. M., Mishra, P., Yaffe, M. B., and Ranganathan, R. (2005) Natural-like function in artificial WW domains. *Nature* **437**, 579-583
10. Socolich, M., Lockless, S. W., Russ, W. P., Lee, H., Gardner, K. H., and Ranganathan, R. (2005) Evolutionary information for specifying a protein fold. *Nature* **437**, 512-518
11. Singh, S., Mandlik, V., and Shinde, S. (2015) Molecular dynamics simulations and statistical coupling analysis of GPI12 in *L. major*: functional co-evolution and conservedness reveals potential drug-target sites. *Mol Biosyst* **11**, 958-968
12. Zhao, Y., Wang, Y., Gao, Y., Li, G., and Huang, J. (2015) Integrated analysis of residue coevolution and protein structures capture key protein sectors in HIV-1 proteins. *PLoS One* **10**, e0117506
13. Tamaki, F. K., Textor, L. C., Polikarpov, I., and Marana, S. R. (2014) Sets of covariant residues modulate the activity and thermal stability of GH1 beta-glucosidases. *PLoS One* **9**, e96627
14. Lee, S. Y., Banerjee, A., and MacKinnon, R. (2009) Two separate interfaces between the voltage sensor and pore are required for the function of voltage-dependent K(+) channels. *PLoS Biol* **7**, e47
15. Grant, B. J., McCammon, J. A., Caves, L. S., and Cross, R. A. (2007) Multivariate analysis of conserved sequence-structure relationships in kinesins: coupling of the active site and a tubulin-binding sub-domain. *J Mol Biol* **368**, 1231-1248
16. Yu, H., Ma, L., Yang, Y., and Cui, Q. (2007) Mechanochemical coupling in the myosin motor domain. II. Analysis of critical residues. *PLoS Computat Biol* **3**, e23
17. Johnson, J. M., Sanford, B. L., Strom, A. M., Tadayon, S. N., Lehman, B. P., Zirbes, A. M., Bhattacharyya, S., Musier-Forsyth, K., and Hati, S. (2013) Multiple pathways promote dynamical coupling between catalytic domains in *Escherichia coli* prolyl-tRNA synthetase. *Biochemistry* **52**, 4399-4412
18. Pless, S. A., Niciforovic, A. P., Galpin, J. D., Nunez, J. J., Kurata, H. T., and Ahern, C. A. (2013) A novel mechanism for fine-tuning open-state stability in a voltage-gated potassium channel. *Nat Commun* **4**, 1784
19. Baths, V., and Roy, U. (2011) Identification of distant co-evolving residues in antigen 85C from Mycobacterium tuberculosis using statistical coupling analysis of the esterase family proteins. *J Biomed Res* **25**, 165-169

20. Campanello, G. C., Ma, Z., Grossoehme, N. E., Guerra, A. J., Ward, B. P., Dimarchi, R. D., Ye, Y., Dann, C. E., 3rd, and Giedroc, D. P. (2013) Allosteric inhibition of a zinc-sensing transcriptional repressor: insights into the arsenic repressor (ArsR) family. *J Mol Biol* **425**, 1143-1157
21. Mendoza, J. L., Schmidt, A., Li, Q., Nuvaga, E., Barrett, T., Bridges, R. J., Feranchak, A. P., Brautigam, C. A., and Thomas, P. J. (2012) Requirements for efficient correction of DeltaF508 CFTR revealed by analyses of evolved sequences. *Cell* **148**, 164-174
22. Strafford, J., Payongsri, P., Hibbert, E. G., Morris, P., Batth, S. S., Steadman, D., Smith, M. E., Ward, J. M., Hailes, H. C., and Dalby, P. A. (2012) Directed evolution to re-adapt a co-evolved network within an enzyme. *J Biotechnol* **157**, 237-245
23. Chen, Z., Rappert, S., Sun, J., and Zeng, A. P. (2011) Integrating molecular dynamics and co-evolutionary analysis for reliable target prediction and deregulation of the allosteric inhibition of aspartokinase for amino acid production. *J Biotechnol* **154**, 248-254
24. Martin-Galiano, A. J., Buey, R. M., Cabezas, M., and Andreu, J. M. (2010) Mapping flexibility and the assembly switch of cell division protein FtsZ by computational and mutational approaches. *J Biol Chem* **285**, 22554-22565
25. Hsu, Y. H., and Traugh, J. A. (2010) Reciprocally coupled residues crucial for protein kinase Pak2 activity calculated by statistical coupling analysis. *PLoS One* **5**, e9455
26. Xu, F., Du, P., Shen, H., Hu, H., Wu, Q., Xie, J., and Yu, L. (2009) Correlated mutation analysis on the catalytic domains of serine/threonine protein kinases. *PLoS One* **4**, e5913
27. Weimer, K. M., Shane, B. L., Brunetto, M., Bhattacharyya, S., and Hati, S. (2009) Evolutionary basis for the coupled-domain motions in *Thermus thermophilus* leucyl-tRNA synthetase. *J Biol Chem* **284**, 10088-10099
28. Nascimento, A. S., Krauchenco, S., Golubev, A. M., Gustchina, A., Wlodawer, A., and Polikarpov, I. (2008) Statistical coupling analysis of aspartic proteinases based on crystal structures of the *Trichoderma reesei* enzyme and its complex with pepstatin A. *J Mol Biol* **382**, 763-778
29. Ozkan, E., Yu, H., and Deisenhofer, J. (2005) Mechanistic insight into the allosteric activation of a ubiquitin-conjugating enzyme by RING-type ubiquitin ligases. *Proc Natl Acad Sci U.S.A.* **102**, 18890-18895
30. Estabrook, R. A., Luo, J., Purdy, M. M., Sharma, V., Weakliem, P., Bruice, T. C., and Reich, N. O. (2005) Statistical coevolution analysis and molecular dynamics: Identification of amino acid pairs essential for catalysis. *Proc Natl Acad Sci U.S.A.* **102**, 994-999
31. Lee, J., Natarajan, M., Nashine, V. C., Socolich, M., Vo, T., Russ, W. P., Benkovic, S. J., and Ranganathan, R. (2008) Surface sites for engineering allosteric control in proteins. *Science* **322**, 438-442
32. Nichols, S. E., Hernandez, C. X., Wang, Y., and McCammon, J. A. (2013) Structure-based network analysis of an evolved G protein-coupled receptor homodimer interface. *Protein Sci* **22**, 745-754
33. Watanabe, L., de Moura, P. R., Bleicher, L., Nascimento, A. S., Zamorano, L. S., Calvete, J. J., Sanz, L., Perez, A., Bursakov, S., Roig, M. G., Shnyrov, V. L., and Polikarpov, I. (2010) Crystal structure and statistical coupling analysis of highly glycosylated peroxidase from royal palm tree (*Roystonea regia*). *J Struct Biol* **169**, 226-242
34. Tehver, R., Chen, J., and Thirumalai, D. (2009) Allostery wiring diagrams in the transitions that drive the GroEL reaction cycle. *J Mol Biol* **387**, 390-406
35. Fuchs, A., Martin-Galiano, A. J., Kalman, M., Fleishman, S., Ben-Tal, N., and Frishman, D. (2007) Co-evolving residues in membrane proteins. *Bioinformatics* **23**, 3312-3319
36. Nene, V., Wortman, J. R., Lawson, D., Haas, B., Kodira, C., Tu, Z. J., Loftus, B., Xi, Z., Megy, K., Grabherr, M., Ren, Q., Zdobnov, E. M., Lobo, N. F., Campbell, K. S., Brown, S. E., Bonaldo, M. F., Zhu, J., Sinkins, S. P., Hogenkamp, D. G., Amedeo, P., Arensburger, P., Atkinson, P. W., Bidwell, S., Biedler, J., Birney, E., Bruggner, R. V., Costas, J., Coy, M. R., Crabtree, J.,

Crawford, M., Debruyn, B., Decaprio, D., Eiglmeier, K., Eisenstadt, E., El-Dorry, H., Gelbart, W. M., Gomes, S. L., Hammond, M., Hannick, L. I., Hogan, J. R., Holmes, M. H., Jaffe, D., Johnston, J. S., Kennedy, R. C., Koo, H., Kravitz, S., Kriventseva, E. V., Kulp, D., Labutti, K., Lee, E., Li, S., Lovin, D. D., Mao, C., Mauceli, E., Menck, C. F., Miller, J. R., Montgomery, P., Mori, A., Nascimento, A. L., Naveira, H. F., Nusbaum, C., O'Leary, S., Orvis, J., Pertea, M., Quesneville, H., Reidenbach, K. R., Rogers, Y. H., Roth, C. W., Schneider, J. R., Schatz, M., Shumway, M., Stanke, M., Stinson, E. O., Tubio, J. M., Vanzee, J. P., Verjovski-Almeida, S., Werner, D., White, O., Wyder, S., Zeng, Q., Zhao, Q., Zhao, Y., Hill, C. A., Raikhel, A. S., Soares, M. B., Knudson, D. L., Lee, N. H., Galagan, J., Salzberg, S. L., Paulsen, I. T., Dimopoulos, G., Collins, F. H., Birren, B., Fraser-Liggett, C. M., and Severson, D. W. (2007) Genome sequence of *Aedes aegypti*, a major arbovirus vector. *Science* **316**, 1718-1723

37. Sharpton, T. J., Stajich, J. E., Rounsley, S. D., Gardner, M. J., Wortman, J. R., Jordar, V. S., Maiti, R., Kodira, C. D., Neafsey, D. E., Zeng, Q., Hung, C. Y., McMahan, C., Muszewska, A., Grynberg, M., Mandel, M. A., Kellner, E. M., Barker, B. M., Galgiani, J. N., Orbach, M. J., Kirkland, T. N., Cole, G. T., Henn, M. R., Birren, B. W., and Taylor, J. W. (2009) Comparative genomic analyses of the human fungal pathogens *Coccidioides* and their relatives. *Genome Res* **19**, 1722-1731

38. Sharakhova, M. V., Hammond, M. P., Lobo, N. F., Krzywinski, J., Unger, M. F., Hillenmeyer, M. E., Bruggner, R. V., Birney, E., and Collins, F. H. (2007) Update of the *Anopheles gambiae* PEST genome assembly. *Genome Biol* **8**, R5

39. Holt, R. A., Subramanian, G. M., Halpern, A., Sutton, G. G., Charlab, R., Nusskern, D. R., Wincker, P., Clark, A. G., Ribeiro, J. M., Wides, R., Salzberg, S. L., Loftus, B., Yandell, M., Majoros, W. H., Rusch, D. B., Lai, Z., Kraft, C. L., Abril, J. F., Anthouard, V., Arensburger, P., Atkinson, P. W., Baden, H., de Berardinis, V., Baldwin, D., Benes, V., Biedler, J., Blass, C., Bolanos, R., Boscus, D., Barnstead, M., Cai, S., Center, A., Chaturverdi, K., Christophides, G. K., Chrystal, M. A., Clamp, M., Cravchik, A., Curwen, V., Dana, A., Delcher, A., Dew, I., Evans, C. A., Flanigan, M., Grundschober-Freimoser, A., Friedli, L., Gu, Z., Guan, P., Guigo, R., Hillenmeyer, M. E., Hladun, S. L., Hogan, J. R., Hong, Y. S., Hoover, J., Jaillon, O., Ke, Z., Kodira, C., Kokoza, E., Koutsos, A., Letunic, I., Levitsky, A., Liang, Y., Lin, J. J., Lobo, N. F., Lopez, J. R., Malek, J. A., McIntosh, T. C., Meister, S., Miller, J., Mobarry, C., Mongin, E., Murphy, S. D., O'Brochta, D. A., Pfannkoch, C., Qi, R., Regier, M. A., Remington, K., Shao, H., Sharakhova, M. V., Sitter, C. D., Shetty, J., Smith, T. J., Strong, R., Sun, J., Thomasova, D., Ton, L. Q., Topalis, P., Tu, Z., Unger, M. F., Walenz, B., Wang, A., Wang, J., Wang, M., Wang, X., Woodford, K. J., Wortman, J. R., Wu, M., Yao, A., Zdobnov, E. M., Zhang, H., Zhao, Q., Zhao, S., Zhu, S. C., Zhimulev, I., Coluzzi, M., della Torre, A., Roth, C. W., Louis, C., Kalush, F., Mural, R. J., Myers, E. W., Adams, M. D., Smith, H. O., Broder, S., Gardner, M. J., Fraser, C. M., Birney, E., Bork, P., Brey, P. T., Venter, J. C., Weissenbach, J., Kafatos, F. C., Collins, F. H., and Hoffman, S. L. (2002) The genome sequence of the malaria mosquito *Anopheles gambiae*. *Science* **298**, 129-149

40. Puthanveettil, S. V., Monje, F. J., Miniaci, M. C., Choi, Y. B., Karl, K. A., Khandros, E., Gawinowicz, M. A., Sheetz, M. P., and Kandel, E. R. (2008) A new component in synaptic plasticity: upregulation of kinesin in the neurons of the gill-withdrawal reflex. *Cell* **135**, 960-973

41. Mitsui, H., Yamaguchi-Shinozaki, K., Shinozaki, K., Nishikawa, K., and Takahashi, H. (1993) Identification of a gene family (kat) encoding kinesin-like proteins in *Arabidopsis thaliana* and the characterization of secondary structure of KatA. *Mol Gen Genet* **238**, 362-368

42. Mitsui, H., Nakatani, K., Yamaguchi-Shinozaki, K., Shinozaki, K., Nishikawa, K., and Takahashi, H. (1994) Sequencing and characterization of the kinesin-related genes katB and katC of *Arabidopsis thaliana*. *Plant Mol Biol* **25**, 865-876

43. Yamada, K., Lim, J., Dale, J. M., Chen, H., Shinn, P., Palm, C. J., Southwick, A. M., Wu, H. C., Kim, C., Nguyen, M., Pham, P., Cheuk, R., Karlin-Newmann, G., Liu, S. X., Lam, B., Sakano, H., Wu, T., Yu, G., Miranda, M., Quach, H. L., Tripp, M., Chang, C. H., Lee, J. M., Toriumi, M.,

Chan, M. M., Tang, C. C., Onodera, C. S., Deng, J. M., Akiyama, K., Ansari, Y., Arakawa, T., Banh, J., Banno, F., Bowser, L., Brooks, S., Carninci, P., Chao, Q., Choy, N., Enju, A., Goldsmith, A. D., Gurjal, M., Hansen, N. F., Hayashizaki, Y., Johnson-Hopson, C., Hsuan, V. W., Iida, K., Karnes, M., Khan, S., Koesema, E., Ishida, J., Jiang, P. X., Jones, T., Kawai, J., Kamiya, A., Meyers, C., Nakajima, M., Narusaka, M., Seki, M., Sakurai, T., Satou, M., Tamse, R., Vaysberg, M., Wallender, E. K., Wong, C., Yamamura, Y., Yuan, S., Shinozaki, K., Davis, R. W., Theologis, A., and Ecker, J. R. (2003) Empirical analysis of transcriptional activity in the *Arabidopsis* genome. *Science* **302**, 842-846

44. Lin, X., Kaul, S., Rounsley, S., Shea, T. P., Benito, M. I., Town, C. D., Fujii, C. Y., Mason, T., Bowman, C. L., Barnstead, M., Feldblyum, T. V., Buell, C. R., Ketchum, K. A., Lee, J., Ronning, C. M., Koo, H. L., Moffat, K. S., Cronin, L. A., Shen, M., Pai, G., Van Aken, S., Umayam, L., Tallon, L. J., Gill, J. E., Adams, M. D., Carrera, A. J., Creasy, T. H., Goodman, H. M., Somerville, C. R., Copenhaver, G. P., Preuss, D., Nierman, W. C., White, O., Eisen, J. A., Salzberg, S. L., Fraser, C. M., and Venter, J. C. (1999) Sequence and analysis of chromosome 2 of the plant *Arabidopsis thaliana*. *Nature* **402**, 761-768

45. Mayer, K., Schuller, C., Wambutt, R., Murphy, G., Volckaert, G., Pohl, T., Dusterhoft, A., Stiekema, W., Entian, K. D., Terryn, N., Harris, B., Ansorge, W., Brandt, P., Grivell, L., Rieger, M., Weichselgartner, M., de Simone, V., Obermaier, B., Mache, R., Muller, M., Kreis, M., Delseny, M., Puigdomenech, P., Watson, M., Schmidtheini, T., Reichert, B., Portatelle, D., Perez-Alonso, M., Boutry, M., Bancroft, I., Vos, P., Hoheisel, J., Zimmermann, W., Wedler, H., Ridley, P., Langham, S. A., McCullagh, B., Bilham, L., Robben, J., Van der Schueren, J., Grymonprez, B., Chuang, Y. J., Vandenbussche, F., Braeken, M., Weltjens, I., Voet, M., Bastiaens, I., Aert, R., Defoor, E., Weitzenegger, T., Bothe, G., Ramsperger, U., Hilbert, H., Braun, M., Holzer, E., Brandt, A., Peters, S., van Staveren, M., Dirske, W., Mooijman, P., Klein Lankhorst, R., Rose, M., Hauf, J., Kotter, P., Berneiser, S., Hempel, S., Feldpausch, M., Lamberth, S., Van den Daele, H., De Keyser, A., Buysshaert, C., Gielen, J., Villarroel, R., De Clercq, R., Van Montagu, M., Rogers, J., Cronin, A., Quail, M., Bray-Allen, S., Clark, L., Doggett, J., Hall, S., Kay, M., Lennard, N., McLay, K., Mayes, R., Pettett, A., Rajandream, M. A., Lyne, M., Benes, V., Rechmann, S., Borkova, D., Blocker, H., Scharfe, M., Grimm, M., Lohnert, T. H., Dose, S., de Haan, M., Maarse, A., Schafer, M., Muller-Auer, S., Gabel, C., Fuchs, M., Fartmann, B., Granderath, K., Dauner, D., Herzl, A., Neumann, S., Argiriou, A., Vitale, D., Liguori, R., Piravandi, E., Massenet, O., Quigley, F., Clabauld, G., Mundlein, A., Felber, R., Schnabl, S., Hiller, R., Schmidt, W., Lecharny, A., Aubourg, S., Chefdor, F., Cooke, R., Berger, C., Montfort, A., Casacuberta, E., Gibbons, T., Weber, N., Vandenbol, M., Bargues, M., Terol, J., Torres, A., Perez-Perez, A., Purnelle, B., Bent, E., Johnson, S., Tacon, D., Jesse, T., Heijnen, L., Schwarz, S., Scholler, P., Heber, S., Francs, P., Bielke, C., Frishman, D., Haase, D., Lemcke, K., Mewes, H. W., Stocker, S., Zaccaria, P., Bevan, M., Wilson, R. K., de la Bastide, M., Habermann, K., Parnell, L., Dedhia, N., Gnoj, L., Schutz, K., Huang, E., Spiegel, L., Sehkon, M., Murray, J., Sheet, P., Cordes, M., Abu-Threideh, J., Stoneking, T., Kalicki, J., Graves, T., Harmon, G., Edwards, J., Latreille, P., Courtney, L., Cloud, J., Abbott, A., Scott, K., Johnson, D., Minx, P., Bentley, D., Fulton, B., Miller, N., Greco, T., Kemp, K., Kramer, J., Fulton, L., Mardis, E., Dante, M., Pepin, K., Hillier, L., Nelson, J., Spieth, J., Ryan, E., Andrews, S., Geisel, C., Layman, D., Du, H., Ali, J., Berghoff, A., Jones, K., Drone, K., Cotton, M., Joshu, C., Antonoiu, B., Zidanic, M., Strong, C., Sun, H., Lamar, B., Yordan, C., Ma, P., Zhong, J., Preston, R., Vil, D., Shekher, M., Matero, A., Shah, R., Swaby, I. K., O'Shaughnessy, A., Rodriguez, M., Hoffmann, J., Till, S., Granat, S., Shohdy, N., Hasegawa, A., Hameed, A., Lodhi, M., Johnson, A., Chen, E., Marra, M., Martienssen, R., and McCombie, W. R. (1999) Sequence and analysis of chromosome 4 of the plant *Arabidopsis* thaliana. *Nature* **402**, 769-777

46. Salanoubat, M., Lemcke, K., Rieger, M., Ansorge, W., Unseld, M., Fartmann, B., Valle, G., Blocker, H., Perez-Alonso, M., Obermaier, B., Delseny, M., Boutry, M., Grivell, L. A., Mache, R., Puigdomenech, P., De Simone, V., Choisne, N., Artiguenave, F., Robert, C., Brottier, P.,

Wincker, P., Cattolico, L., Weissenbach, J., Saurin, W., Quetier, F., Schafer, M., Muller-Auer, S., Gabel, C., Fuchs, M., Benes, V., Wurmbach, E., Drzonek, H., Erfle, H., Jordan, N., Bangert, S., Wiedelmann, R., Kranz, H., Voss, H., Holland, R., Brandt, P., Nyakatura, G., Vezzi, A., D'Angelo, M., Pallavicini, A., Toppo, S., Simionati, B., Conrad, A., Hornischer, K., Kauer, G., Lohnert, T. H., Nordsiek, G., Reichelt, J., Scharfe, M., Schon, O., Bargues, M., Terol, J., Climent, J., Navarro, P., Collado, C., Perez-Perez, A., Ottenwalder, B., Duchemin, D., Cooke, R., Laudie, M., Berger-Llauro, C., Purnelle, B., Masuy, D., de Haan, M., Maarse, A. C., Alcaraz, J. P., Cottet, A., Casacuberta, E., Monfort, A., Argiriou, A., flores, M., Liguori, R., Vitale, D., Mannhaupt, G., Haase, D., Schoof, H., Rudd, S., Zaccaria, P., Mewes, H. W., Mayer, K. F., Kaul, S., Town, C. D., Koo, H. L., Tallon, L. J., Jenkins, J., Rooney, T., Rizzo, M., Walts, A., Utterback, T., Fujii, C. Y., Shea, T. P., Creasy, T. H., Haas, B., Maiti, R., Wu, D., Peterson, J., Van Aken, S., Pai, G., Militscher, J., Sellers, P., Gill, J. E., Feldblyum, T. V., Preuss, D., Lin, X., Nierman, W. C., Salzberg, S. L., White, O., Venter, J. C., Fraser, C. M., Kaneko, T., Nakamura, Y., Sato, S., Kato, T., Asamizu, E., Sasamoto, S., Kimura, T., Idesawa, K., Kawashima, K., Kishida, Y., Kiyokawa, C., Kohara, M., Matsumoto, M., Matsuno, A., Muraki, A., Nakayama, S., Nakazaki, N., Shinpo, S., Takeuchi, C., Wada, T., Watanabe, A., Yamada, M., Yasuda, M., Tabata, S., European Union Chromosome 3 Arabidopsis Sequencing Consortium, Institute for Genomic Research, and Kazusa DNA Research Institute (2000) Sequence and analysis of chromosome 3 of the plant *Arabidopsis* thaliana. *Nature* **408**, 820-822

47. Kato, A., Suzuki, M., Kuwahara, A., Ooe, H., Higano-Inaba, K., and Komeda, Y. (1999) Isolation and analysis of cDNA within a 300 kb *Arabidopsis thaliana* genomic region located around the 100 map unit of chromosome 1. *Gene* **239**, 309-316

48. Theologis, A., Ecker, J. R., Palm, C. J., Federspiel, N. A., Kaul, S., White, O., Alonso, J., Altafi, H., Araujo, R., Bowman, C. L., Brooks, S. Y., Buehler, E., Chan, A., Chao, Q., Chen, H., Cheuk, R. F., Chin, C. W., Chung, M. K., Conn, L., Conway, A. B., Conway, A. R., Creasy, T. H., Dewar, K., Dunn, P., Etgu, P., Feldblyum, T. V., Feng, J., Fong, B., Fujii, C. Y., Gill, J. E., Goldsmith, A. D., Haas, B., Hansen, N. F., Hughes, B., Huizar, L., Hunter, J. L., Jenkins, J., Johnson-Hopson, C., Khan, S., Khaykin, E., Kim, C. J., Koo, H. L., Kremenetskaia, I., Kurtz, D. B., Kwan, A., Lam, B., Langin-Hooper, S., Lee, A., Lee, J. M., Lenz, C. A., Li, J. H., Li, Y., Lin, X., Liu, S. X., Liu, Z. A., Luros, J. S., Maiti, R., Marziali, A., Militscher, J., Miranda, M., Nguyen, M., Nierman, W. C., Osborne, B. I., Pai, G., Peterson, J., Pham, P. K., Rizzo, M., Rooney, T., Rowley, D., Sakano, H., Salzberg, S. L., Schwartz, J. R., Shinn, P., Southwick, A. M., Sun, H., Tallon, L. J., Tambunga, G., Toriumi, M. J., Town, C. D., Utterback, T., Van Aken, S., Vaysberg, M., Vysotskaia, V. S., Walker, M., Wu, D., Yu, G., Fraser, C. M., Venter, J. C., and Davis, R. W. (2000) Sequence and analysis of chromosome 1 of the plant *Arabidopsis thaliana*. *Nature* **408**, 816-820

49. Tabata, S., Kaneko, T., Nakamura, Y., Kotani, H., Kato, T., Asamizu, E., Miyajima, N., Sasamoto, S., Kimura, T., Hosouchi, T., Kawashima, K., Kohara, M., Matsumoto, M., Matsuno, A., Muraki, A., Nakayama, S., Nakazaki, N., Naruo, K., Okumura, S., Shinpo, S., Takeuchi, C., Wada, T., Watanabe, A., Yamada, M., Yasuda, M., Sato, S., de la Bastide, M., Huang, E., Spiegel, L., Gnoj, L., O'Shaughnessy, A., Preston, R., Habermann, K., Murray, J., Johnson, D., Rohlfing, T., Nelson, J., Stoneking, T., Pepin, K., Spieth, J., Sekhon, M., Armstrong, J., Becker, M., Belter, E., Cordum, H., Cordes, M., Courtney, L., Courtney, W., Dante, M., Du, H., Edwards, J., Fryman, J., Haakensen, B., Lamar, E., Latreille, P., Leonard, S., Meyer, R., Mulvaney, E., Ozersky, P., Riley, A., Strowmatt, C., Wagner-McPherson, C., Wollam, A., Yoakum, M., Bell, M., Dedhia, N., Parnell, L., Shah, R., Rodriguez, M., See, L. H., Vil, D., Baker, J., Kirchoff, K., Toth, K., King, L., Bahret, A., Miller, B., Marra, M., Martienssen, R., McCombie, W. R., Wilson, R. K., Murphy, G., Bancroft, I., Volckaert, G., Wambutt, R., Dusterhoft, A., Stiekema, W., Pohl, T., Entian, K. D., Terryn, N., Hartley, N., Bent, E., Johnson, S., Langham, S. A., McCullagh, B., Robben, J., Grymonprez, B., Zimmermann, W., Ramsperger, U., Wedler, H., Balke, K., Wedler, E., Peters, S., van Staveren, M., Dirkse, W., Mooijman, P., Lankhorst, R. K., Weitzenegger, T.,

Bothe, G., Rose, M., Hauf, J., Berneiser, S., Hempel, S., Feldpausch, M., Lamberth, S., Villarroel, R., Gielen, J., Ardiles, W., Bents, O., Lemcke, K., Kolesov, G., Mayer, K., Rudd, S., Schoof, H., Schueller, C., Zaccaria, P., Mewes, H. W., Bevan, M., Fransz, P., Kazusa DNA Research Institute, The Cold Spring Harbor and Washington University in St. Louis Sequencing Consortium, and The European Union Arabidopsis Genome Sequencing Consortium. (2000) Sequence and analysis of chromosome 5 of the plant *Arabidopsis thaliana*. *Nature* **408**, 823-826

50. Lee, Y. R., and Liu, B. (2000) Identification of a phragmoplast-associated kinesin-related protein in higher plants. *Curr Biol* **10**, 797-800
51. Sakai, T., Honing, H., Nishioka, M., Uehara, Y., Takahashi, M., Fujisawa, N., Saji, K., Seki, M., Shinozaki, K., Jones, M. A., Smirnoff, N., Okada, K., and Wasteneys, G. O. (2008) Armadillo repeat-containing kinesins and a NIMA-related kinase are required for epidermal-cell morphogenesis in *Arabidopsis*. *Plant J* **53**, 157-171
52. Kong, L. J., and Hanley-Bowdoin, L. (2002) A geminivirus replication protein interacts with a protein kinase and a motor protein that display different expression patterns during plant development and infection. *Plant Cell* **14**, 1817-1832
53. Iida, K., Fukami-Kobayashi, K., Toyoda, A., Sakaki, Y., Kobayashi, M., Seki, M., and Shinozaki, K. (2009) Analysis of multiple occurrences of alternative splicing events in *Arabidopsis thaliana* using novel sequenced full-length cDNAs. *DNA Res* **16**, 155-164
54. Dietrich, F. S., Voegeli, S., Brachat, S., Lerch, A., Gates, K., Steiner, S., Mohr, C., Pohlmann, R., Luedi, P., Choi, S., Wing, R. A., Flavier, A., Gaffney, T. D., and Philippsen, P. (2004) The *Ashbya gossypii* genome as a tool for mapping the ancient *Saccharomyces cerevisiae* genome. *Science* **304**, 304-307
55. Fedorova, N. D., Khaldi, N., Joardar, V. S., Maiti, R., Amedeo, P., Anderson, M. J., Crabtree, J., Silva, J. C., Badger, J. H., Albarraq, A., Angiuoli, S., Bussey, H., Bowyer, P., Cotty, P. J., Dyer, P. S., Egan, A., Galens, K., Fraser-Liggett, C. M., Haas, B. J., Inman, J. M., Kent, R., Lemieux, S., Malavazi, I., Orvis, J., Roemer, T., Ronning, C. M., Sundaram, J. P., Sutton, G., Turner, G., Venter, J. C., White, O. R., Whitty, B. R., Youngman, P., Wolfe, K. H., Goldman, G. H., Wortman, J. R., Jiang, B., Denning, D. W., and Nierman, W. C. (2008) Genomic islands in the pathogenic filamentous fungus *Aspergillus fumigatus*. *PLoS Genet* **4**, e1000046
56. Nierman, W. C., Pain, A., Anderson, M. J., Wortman, J. R., Kim, H. S., Arroyo, J., Berriman, M., Abe, K., Archer, D. B., Bermejo, C., Bennett, J., Bowyer, P., Chen, D., Collins, M., Coulsen, R., Davies, R., Dyer, P. S., Farman, M., Fedorova, N., Feldblyum, T. V., Fischer, R., Fosker, N., Fraser, A., Garcia, J. L., Garcia, M. J., Goble, A., Goldman, G. H., Gomi, K., Griffith-Jones, S., Gwilliam, R., Haas, B., Haas, H., Harris, D., Horiuchi, H., Huang, J., Humphray, S., Jimenez, J., Keller, N., Khouri, H., Kitamoto, K., Kobayashi, T., Konzack, S., Kulkarni, R., Kumagai, T., Lafon, A., Latge, J. P., Li, W., Lord, A., Lu, C., Majoros, W. H., May, G. S., Miller, B. L., Mohamoud, Y., Molina, M., Monod, M., Mouyna, I., Mulligan, S., Murphy, L., O'Neil, S., Paulsen, I., Penalva, M. A., Pertea, M., Price, C., Pritchard, B. L., Quail, M. A., Rabbinowitsch, E., Rawlins, N., Rajandream, M. A., Reichard, U., Renauld, H., Robson, G. D., Rodriguez de Cordoba, S., Rodriguez-Pena, J. M., Ronning, C. M., Rutter, S., Salzberg, S. L., Sanchez, M., Sanchez-Ferrero, J. C., Saunders, D., Seeger, K., Squares, R., Squares, S., Takeuchi, M., Tekaia, F., Turner, G., Vazquez de Aldana, C. R., Weidman, J., White, O., Woodward, J., Yu, J. H., Fraser, C., Galagan, J. E., Asai, K., Machida, M., Hall, N., Barrell, B., and Denning, D. W. (2005) Genomic sequence of the pathogenic and allergenic filamentous fungus *Aspergillus fumigatus*. *Nature* **438**, 1151-1156
57. Pel, H. J., de Winde, J. H., Archer, D. B., Dyer, P. S., Hofmann, G., Schaap, P. J., Turner, G., de Vries, R. P., Albang, R., Albermann, K., Andersen, M. R., Bendtsen, J. D., Benen, J. A., van den Berg, M., Breestraat, S., Caddick, M. X., Contreras, R., Cornell, M., Coutinho, P. M., Danchin, E. G., Debets, A. J., Dekker, P., van Dijck, P. W., van Dijk, A., Dijkhuizen, L., Driessen, A. J., d'Enfert, C., Geysens, S., Goosen, C., Groot, G. S., de Groot, P. W., Guillemette, T., Henrissat, B., Herweijer, M., van den Hombergh, J. P., van den Hondel, C. A., van der Heijden, R. T., van

der Kaaij, R. M., Klis, F. M., Kools, H. J., Kubicek, C. P., van Kuyk, P. A., Lauber, J., Lu, X., van der Maarel, M. J., Meulenberg, R., Menke, H., Mortimer, M. A., Nielsen, J., Oliver, S. G., Olsthoorn, M., Pal, K., van Peij, N. N., Ram, A. F., Rinas, U., Roubos, J. A., Sagt, C. M., Schmoll, M., Sun, J., Ussery, D., Varga, J., Vervecken, W., van de Vondervoort, P. J., Wedler, H., Wosten, H. A., Zeng, A. P., van Ooyen, A. J., Visser, J., and Stam, H. (2007) Genome sequencing and analysis of the versatile cell factory *Aspergillus niger* CBS 513.88. *Nat Biotechnol* **25**, 221-231

58. Birren, B., Lander, E., Galagan, J., Nusbaum, C., Devon, K., Henn, M., Ma, L. J., Jaffe, D., Butler, J., Alvarez, P., Gnerre, S., Grabherr, M., Kleber, M., Mauceli, E., Brockman, W., Rounsley, S., Young, S., LaButti, K., Pushparaj, V., DeCaprio, D., Crawford, M., Koehrsen, M., Engels, R., Montgomery, P., Pearson, M., Howarth, C., Larson, L., Luoma, S., White, J., Alvarado, L., Kodira, C., Zeng, Q., Oleary, S., Yandava, C., Denning, D., Nierman, B., Milne, T., and Madden, K. (2005) Annotation of the *Aspergillus terreus* NIH2624 genome. Broad Institute of MIT and Harvard, Cambridge. Direct submission to EMBL/GenBank/DDBJ databases

59. Brayton, K. A., Lau, A. O., Herndon, D. R., Hannick, L., Kappmeyer, L. S., Berens, S. J., Bidwell, S. L., Brown, W. C., Crabtree, J., Fadrosh, D., Feldblum, T., Forberger, H. A., Haas, B. J., Howell, J. M., Khouri, H., Koo, H., Mann, D. J., Norimine, J., Paulsen, I. T., Radune, D., Ren, Q., Smith, R. K., Jr., Suarez, C. E., White, O., Wortman, J. R., Knowles, D. P., Jr., McElwain, T. F., and Nene, V. M. (2007) Genome sequence of *Babesia bovis* and comparative analysis of apicomplexan hemoprotozoa. *PLoS Pathog* **3**, 1401-1413

60. Okano, K., Takada, N., Kobayashi, M., and Maekawa, H. (1994) cDNA structure and characterization of a kinesin-like protein from the silkworm *Bombyx mori*. *Insect Mol Biol* **3**, 195-200

61. Wang, Q., Teng, J., Shen, B., Zhang, W., Guo, Y., Su, X., Zhang, C., Yu, A. C., and Chen, J. (2010) Characterization of kinesin-like proteins in silkworm posterior silk gland cells. *Cell Res* **20**, 713-727

62. Bovine Genome Sequencing and Analysis Consortium, Elsik, C. G., Tellam, R. L., Worley, K. C., Gibbs, R. A., Muzny, D. M., Weinstock, G. M., Adelson, D. L., Eichler, E. E., Elnitski, L., Guigo, R., Hamernik, D. L., Kappes, S. M., Lewin, H. A., Lynn, D. J., Nicholas, F. W., Reymond, A., Rijnkels, M., Skow, L. C., Zdobnov, E. M., Schook, L., Womack, J., Alioto, T., Antonarakis, S. E., Astashyn, A., Chapple, C. E., Chen, H. C., Chrast, J., Camara, F., Ermolaeva, O., Henrichsen, C. N., Hlavina, W., Kapustin, Y., Kiryutin, B., Kitts, P., Kokocinski, F., Landrum, M., Maglott, D., Pruitt, K., Sapojnikov, V., Searle, S. M., Solovyev, V., Souvorov, A., Ucla, C., Wyss, C., Anzola, J. M., Gerlach, D., Elhaik, E., Graur, D., Reese, J. T., Edgar, R. C., McEwan, J. C., Payne, G. M., Raison, J. M., Junier, T., Kriventseva, E. V., Eyras, E., Plass, M., Donthu, R., Larkin, D. M., Reecy, J., Yang, M. Q., Chen, L., Cheng, Z., Chitko-McKown, C. G., Liu, G. E., Matukumalli, L. K., Song, J., Zhu, B., Bradley, D. G., Brinkman, F. S., Lau, L. P., Whiteside, M. D., Walker, A., Wheeler, T. T., Casey, T., German, J. B., Lemay, D. G., Maqbool, N. J., Molenaar, A. J., Seo, S., Stothard, P., Baldwin, C. L., Baxter, R., Brinkmeyer-Langford, C. L., Brown, W. C., Childers, C. P., Connelley, T., Ellis, S. A., Fritz, K., Glass, E. J., Herzig, C. T., Iivanainen, A., Lahmers, K. K., Bennett, A. K., Dickens, C. M., Gilbert, J. G., Hagen, D. E., Salih, H., Aerts, J., Caetano, A. R., Dalrymple, B., Garcia, J. F., Gill, C. A., Hiendleder, S. G., Memili, E., Spurlock, D., Williams, J. L., Alexander, L., Brownstein, M. J., Guan, L., Holt, R. A., Jones, S. J., Marra, M. A., Moore, R., Moore, S. S., Roberts, A., Taniguchi, M., Waterman, R. C., Chacko, J., Chandrabose, M. M., Cree, A., Dao, M. D., Dinh, H. H., Gabisi, R. A., Hines, S., Hume, J., Jhangiani, S. N., Joshi, V., Kovar, C. L., Lewis, L. R., Liu, Y. S., Lopez, J., Morgan, M. B., Nguyen, N. B., Okwuonu, G. O., Ruiz, S. J., Santibanez, J., Wright, R. A., Buhay, C., Ding, Y., Dugan-Rocha, S., Herdandez, J., Holder, M., Sabo, A., Egan, A., Goodell, J., Wilczek-Boney, K., Fowler, G. R., Hitchens, M. E., Lozado, R. J., Moen, C., Steffen, D., Warren, J. T., Zhang, J., Chiu, R., Schein, J. E., Durbin, K. J., Havlak, P., Jiang, H., Liu, Y., Qin, X., Ren, Y., Shen, Y., Song, H., Bell, S. N., Davis, C., Johnson, A. J., Lee, S., Nazareth, L. V., Patel, B. M.,

Pu, L. L., Vattathil, S., Williams, R. L., Jr., Curry, S., Hamilton, C., Sodergren, E., Wheeler, D. A., Barris, W., Bennett, G. L., Eggen, A., Green, R. D., Harhay, G. P., Hobbs, M., Jann, O., Keele, J. W., Kent, M. P., Lien, S., McKay, S. D., McWilliam, S., Ratnakumar, A., Schnabel, R. D., Smith, T., Snelling, W. M., Sonstegard, T. S., Stone, R. T., Sugimoto, Y., Takasuga, A., Taylor, J. F., Van Tassell, C. P., Macneil, M. D., Abatepaulo, A. R., Abbey, C. A., Ahola, V., Almeida, I. G., Amadio, A. F., Anatriello, E., Bahadue, S. M., Biase, F. H., Boldt, C. R., Carroll, J. A., Carvalho, W. A., Cervelatti, E. P., Chacko, E., Chapin, J. E., Cheng, Y., Choi, J., Colley, A. J., de Campos, T. A., De Donato, M., Santos, I. K., de Oliveira, C. J., Deobald, H., Devinoy, E., Donohue, K. E., Dovc, P., Eberlein, A., Fitzsimmons, C. J., Franzin, A. M., Garcia, G. R., Genini, S., Gladney, C. J., Grant, J. R., Greaser, M. L., Green, J. A., Hadsell, D. L., Hakimov, H. A., Halgren, R., Harrow, J. L., Hart, E. A., Hastings, N., Hernandez, M., Hu, Z. L., Ingham, A., Iso-Touru, T., Jamis, C., Jensen, K., Kapetis, D., Kerr, T., Khalil, S. S., Khatib, H., Kolbehdari, D., Kumar, C. G., Kumar, D., Leach, R., Lee, J. C., Li, C., Logan, K. M., Malinverni, R., Marques, E., Martin, W. F., Martins, N. F., Maruyama, S. R., Mazza, R., McLean, K. L., Medrano, J. F., Moreno, B. T., More, D. D., Muntean, C. T., Nandakumar, H. P., Nogueira, M. F., Olsaker, I., Pant, S. D., Panzitta, F., Pastor, R. C., Poli, M. A., Poslusny, N., Rachagani, S., Ranganathan, S., Razpet, A., Riggs, P. K., Rincon, G., Rodriguez-Osorio, N., Rodriguez-Zas, S. L., Romero, N. E., Rosenwald, A., Sando, L., Schmutz, S. M., Shen, L., Sherman, L., Southey, B. R., Lutzow, Y. S., Sweedler, J. V., Tammen, I., Telugu, B. P., Urbanski, J. M., Utsunomiya, Y. T., Verschoor, C. P., Waardenberg, A. J., Wang, Z., Ward, R., Weikard, R., Welsh, T. H., Jr., White, S. N., Wilming, L. G., Wunderlich, K. R., Yang, J., and Zhao, F. Q. (2009) The genome sequence of taurine cattle: a window to ruminant biology and evolution. *Science* **324**, 522-528

63. Zimin, A. V., Delcher, A. L., Florea, L., Kelley, D. R., Schatz, M. C., Puiu, D., Hanrahan, F., Pertea, G., Van Tassell, C. P., Sonstegard, T. S., Marcais, G., Roberts, M., Subramanian, P., Yorke, J. A., and Salzberg, S. L. (2009) A whole-genome assembly of the domestic cow, *Bos taurus*. *Genome biology* **10**, R42
64. Baker, S. A., Freeman, K., Luby-Phelps, K., Pazour, G. J., and Besharse, J. C. (2003) IFT20 links kinesin II with a mammalian intraflagellar transport complex that is conserved in motile flagella and sensory cilia. *J Biol Chem* **278**, 34211-34218
65. Amselem, J., Cuomo, C. A., van Kan, J. A., Viaud, M., Benito, E. P., Couloux, A., Coutinho, P. M., de Vries, R. P., Dyer, P. S., Fillinger, S., Fournier, E., Gout, L., Hahn, M., Kohn, L., Lapalu, N., Plummer, K. M., Pradier, J. M., Quevillon, E., Sharon, A., Simon, A., ten Have, A., Tudzynski, B., Tudzynski, P., Wincker, P., Andrew, M., Anthouard, V., Beever, R. E., Beffa, R., Benoit, I., Bouzid, O., Brault, B., Chen, Z., Choquer, M., Collemare, J., Cotton, P., Danchin, E. G., Da Silva, C., Gautier, A., Giraud, C., Giraud, T., Gonzalez, C., Grossetete, S., Guldener, U., Henrissat, B., Howlett, B. J., Kodira, C., Kretschmer, M., Lappartient, A., Leroch, M., Levis, C., Mauceli, E., Neuveglise, C., Oeser, B., Pearson, M., Poulain, J., Poussereau, N., Quesneville, H., Rascle, C., Schumacher, J., Segurens, B., Sexton, A., Silva, E., Sirven, C., Soanes, D. M., Talbot, N. J., Templeton, M., Yandava, C., Yarden, O., Zeng, Q., Rollins, J. A., Lebrun, M. H., and Dickman, M. (2011) Genomic analysis of the necrotrophic fungal pathogens *Sclerotinia sclerotiorum* and *Botrytis cinerea*. *PLoS Genet* **7**, e1002230
66. Patel, N., Thierry-Mieg, D., and Mancillas, J. R. (1993) Cloning by insertional mutagenesis of a cDNA encoding *Caenorhabditis elegans* kinesin heavy chain. *Proc Natl Acad Sci U.S.A.* **90**, 9181-9185
67. (1998) Genome sequence of the nematode *C. elegans*: a platform for investigating biology. *Science* **282**, 2012-2018
68. Otsuka, A. J., Jeyaprakash, A., Garcia-Anoveros, J., Tang, L. Z., Fisk, G., Hartshorne, T., Franco, R., and Born, T. (1991) The C. elegans unc-104 gene encodes a putative kinesin heavy chain-like protein. *Neuron* **6**, 113-122

69. Tabish, M., Siddiqui, Z. K., Nishikawa, K., and Siddiqui, S. S. (1995) Exclusive expression of *C. elegans* osm-3 kinesin gene in chemosensory neurons open to the external environment. *J Mol Biol* **247**, 377-389
70. Tait, E., Simon, M. C., King, S., Brown, A. J., Gow, N. A., and Shaw, D. J. (1997) A *Candida albicans* genome project: cosmid contigs, physical mapping, and gene isolation. *Fungal Genet Biol* **21**, 308-314
71. Walther, Z., Vashishtha, M., and Hall, J. L. (1994) The *Chlamydomonas* FLA10 gene encodes a novel kinesin-homologous protein. *J Cell Biol* **126**, 175-188
72. Bernstein, M., Beech, P. L., Katz, S. G., and Rosenbaum, J. L. (1994) A new kinesin-like protein (Klp1) localized to a single microtubule of the *Chlamydomonas* flagellum. *J Cell Biol* **125**, 1313-1326
73. Dymek, E. E., Goduti, D., Kramer, T., and Smith, E. F. (2006) A kinesin-like calmodulin-binding protein in *Chlamydomonas*: evidence for a role in cell division and flagellar functions. *J Cell Sci* **119**, 3107-3116
74. Wordeman, L., and Mitchison, T. J. (1995) Identification and partial characterization of mitotic centromere-associated kinesin, a kinesin-related protein that associates with centromeres during mitosis. *J Cell Biol* **128**, 95-104
75. Kuriyama, R., Kofron, M., Essner, R., Kato, T., Dragas-Granoic, S., Omoto, C. K., and Khodjakov, A. (1995) Characterization of a minus end-directed kinesin-like motor protein from cultured mammalian cells. *J Cell Biol* **129**, 1049-1059
76. Abrahamsen, M. S., Templeton, T. J., Enomoto, S., Abrahante, J. E., Zhu, G., Lancto, C. A., Deng, M., Liu, C., Widmer, G., Tzipori, S., Buck, G. A., Xu, P., Bankier, A. T., Dear, P. H., Konfortov, B. A., Spriggs, H. F., Iyer, L., Anantharaman, V., Aravind, L., and Kapur, V. (2004) Complete genome sequence of the apicomplexan, *Cryptosporidium parvum*. *Science* **304**, 441-445
77. Deleu, W., Gonzalez, V., Monfort, A., Bendahmane, A., Puigdomenech, P., Arus, P., and Garcia-Mas, J. (2007) Structure of two melon regions reveals high microsynteny with sequenced plant species. *Mol Genet Genomics* **278**, 611-622
78. Tay, S. Y., Ingham, P. W., and Roy, S. (2005) A homologue of the *Drosophila* kinesin-like protein Costal2 regulates Hedgehog signal transduction in the vertebrate embryo. *Development* **132**, 625-634
79. Humphries, M., Johnson, C., Howden, P., Wright, D., and Raisen, C. (2009) The *Danio rerio* Sequencing Project Wellcome Trust Sanger Institute, Hinxton. Direct submission to EMBL/GenBank/DDBJ databases
80. Strausberg, R. L., Feingold, E. A., Grouse, L. H., Derge, J. G., Klausner, R. D., Collins, F. S., Wagner, L., Shenmen, C. M., Schuler, G. D., Altschul, S. F., Zeeberg, B., Buetow, K. H., Schaefer, C. F., Bhat, N. K., Hopkins, R. F., Jordan, H., Moore, T., Max, S. I., Wang, J., Hsieh, F., Diatchenko, L., Marusina, K., Farmer, A. A., Rubin, G. M., Hong, L., Stapleton, M., Soares, M. B., Bonaldo, M. F., Casavant, T. L., Scheetz, T. E., Brownstein, M. J., Usdin, T. B., Toshiyuki, S., Carninci, P., Prange, C., Raha, S. S., Loquellano, N. A., Peters, G. J., Abramson, R. D., Mullahy, S. J., Bosak, S. A., McEwan, P. J., McKernan, K. J., Malek, J. A., Gunaratne, P. H., Richards, S., Worley, K. C., Hale, S., Garcia, A. M., Gay, L. J., Hulyk, S. W., Villalon, D. K., Muzny, D. M., Sodergren, E. J., Lu, X., Gibbs, R. A., Fahey, J., Helton, E., Ketteman, M., Madan, A., Rodrigues, S., Sanchez, A., Whiting, M., Young, A. C., Shevchenko, Y., Bouffard, G. G., Blakesley, R. W., Touchman, J. W., Green, E. D., Dickson, M. C., Rodriguez, A. C., Grimwood, J., Schmutz, J., Myers, R. M., Butterfield, Y. S., Krzywinski, M. I., Skalska, U., Smailus, D. E., Schnerch, A., Schein, J. E., Jones, S. J., and Marra, M. A. (2002) Generation and initial analysis of more than 15,000 full-length human and mouse cDNA sequences. *Proc Natl Acad Sci U.S.A.* **99**, 16899-16903

81. Insinna, C., Pathak, N., Perkins, B., Drummond, I., and Besharse, J. C. (2008) The homodimeric kinesin, Kif17, is essential for vertebrate photoreceptor sensory outer segment development. *Dev Biol* **316**, 160-170
82. Eichinger, L., Pachebat, J. A., Glockner, G., Rajandream, M. A., Sucgang, R., Berriman, M., Song, J., Olsen, R., Szafranski, K., Xu, Q., Tunggal, B., Kummerfeld, S., Madera, M., Konfortov, B. A., Rivero, F., Bankier, A. T., Lehmann, R., Hamlin, N., Davies, R., Gaudet, P., Fey, P., Pilcher, K., Chen, G., Saunders, D., Sodergren, E., Davis, P., Kerhornou, A., Nie, X., Hall, N., Anjard, C., Hemphill, L., Bason, N., Farbrother, P., Desany, B., Just, E., Morio, T., Rost, R., Churcher, C., Cooper, J., Haydock, S., van Driessche, N., Cronin, A., Goodhead, I., Muzny, D., Mourier, T., Pain, A., Lu, M., Harper, D., Lindsay, R., Hauser, H., James, K., Quiles, M., Madan Babu, M., Saito, T., Buchrieser, C., Wardroper, A., Felder, M., Thangavelu, M., Johnson, D., Knights, A., Loulseged, H., Mungall, K., Oliver, K., Price, C., Quail, M. A., Urushihara, H., Hernandez, J., Rabbinowitsch, E., Steffen, D., Sanders, M., Ma, J., Kohara, Y., Sharp, S., Simmonds, M., Spiegler, S., Tivey, A., Sugano, S., White, B., Walker, D., Woodward, J., Winckler, T., Tanaka, Y., Shaulsky, G., Schleicher, M., Weinstock, G., Rosenthal, A., Cox, E. C., Chisholm, R. L., Gibbs, R., Loomis, W. F., Platzer, M., Kay, R. R., Williams, J., Dear, P. H., Noegel, A. A., Barrell, B., and Kuspa, A. (2005) The genome of the social amoeba *Dictyostelium discoideum*. *Nature* **435**, 43-57
83. Kollmar, M., and Glockner, G. (2003) Identification and phylogenetic analysis of *Dictyostelium discoideum* kinesin proteins. *BMC genomics* **4**, 47
84. Iwai, S., Ishiji, A., Mabuchi, I., and Sutoh, K. (2004) A novel actin-bundling kinesin-related protein from *Dictyostelium discoideum*. *J Biol Chem* **279**, 4696-4704
85. de Hostos, E. L., McCaffrey, G., Sucgang, R., Pierce, D. W., and Vale, R. D. (1998) A developmentally regulated kinesin-related motor protein from *Dictyostelium discoideum*. *Mol Biol Cell* **9**, 2093-2106
86. Pollock, N., de Hostos, E. L., Turck, C. W., and Vale, R. D. (1999) Reconstitution of membrane transport powered by a novel dimeric kinesin motor of the Unc104/KIF1A family purified from *Dictyostelium*. *J Cell Biol* **147**, 493-506
87. Endow, S. A., Henikoff, S., and Soler-Niedziela, L. (1990) Mediation of meiotic and early mitotic chromosome segregation in *Drosophila* by a protein related to kinesin. *Nature* **345**, 81-83
88. Zhang, P., Knowles, B. A., Goldstein, L. S., and Hawley, R. S. (1990) A kinesin-like protein required for distributive chromosome segregation in *Drosophila*. *Cell* **62**, 1053-1062
89. Stewart, R. J., Pesavento, P. A., Woerpel, D. N., and Goldstein, L. S. (1991) Identification and partial characterization of six members of the kinesin superfamily in *Drosophila*. *Proc Natl Acad Sci U.S.A.* **88**, 8470-8474
90. Adams, M. D., Celniker, S. E., Holt, R. A., Evans, C. A., Gocayne, J. D., Amanatides, P. G., Scherer, S. E., Li, P. W., Hoskins, R. A., Galle, R. F., George, R. A., Lewis, S. E., Richards, S., Ashburner, M., Henderson, S. N., Sutton, G. G., Wortman, J. R., Yandell, M. D., Zhang, Q., Chen, L. X., Brandon, R. C., Rogers, Y. H., Blazej, R. G., Champe, M., Pfeiffer, B. D., Wan, K. H., Doyle, C., Baxter, E. G., Helt, G., Nelson, C. R., Gabor, G. L., Abril, J. F., Agbayani, A., An, H. J., Andrews-Pfannkoch, C., Baldwin, D., Ballew, R. M., Basu, A., Baxendale, J., Bayraktaroglu, L., Beasley, E. M., Beeson, K. Y., Benos, P. V., Berman, B. P., Bhandari, D., Bolshakov, S., Borkova, D., Botchan, M. R., Bouck, J., Brokstein, P., Brottier, P., Burtis, K. C., Busam, D. A., Butler, H., Cadieu, E., Center, A., Chandra, I., Cherry, J. M., Cawley, S., Dahlke, C., Davenport, L. B., Davies, P., de Pablos, B., Delcher, A., Deng, Z., Mays, A. D., Dew, I., Dietz, S. M., Dodson, K., Doup, L. E., Downes, M., Dugan-Rocha, S., Dunkov, B. C., Dunn, P., Durbin, K. J., Evangelista, C. C., Ferraz, C., Ferriera, S., Fleischmann, W., Fosler, C., Gabrielian, A. E., Garg, N. S., Gelbart, W. M., Glasser, K., Glodek, A., Gong, F., Gorrell, J. H., Gu, Z., Guan, P., Harris, M., Harris, N. L., Harvey, D., Heiman, T. J., Hernandez, J. R., Houck, J., Hostin, D., Houston, K. A., Howland, T. J., Wei, M. H., Ibegwam, C., Jalali, M., Kalush, F., Karpen, G. H., Ke, Z., Kennison, J. A., Ketchum, K. A., Kimmel, B. E., Kodira, C. D., Kraft, C.,

Kravitz, S., Kulp, D., Lai, Z., Lasko, P., Lei, Y., Levitsky, A. A., Li, J., Li, Z., Liang, Y., Lin, X., Liu, X., Mattei, B., McIntosh, T. C., McLeod, M. P., McPherson, D., Merkulov, G., Milshina, N. V., Mobarry, C., Morris, J., Moshrefi, A., Mount, S. M., Moy, M., Murphy, B., Murphy, L., Muzny, D. M., Nelson, D. L., Nelson, D. R., Nelson, K. A., Nixon, K., Nusskern, D. R., Pacleb, J. M., Palazzolo, M., Pittman, G. S., Pan, S., Pollard, J., Puri, V., Reese, M. G., Reinert, K., Remington, K., Saunders, R. D., Scheeler, F., Shen, H., Shue, B. C., Siden-Kiamos, I., Simpson, M., Skupski, M. P., Smith, T., Spier, E., Spradling, A. C., Stapleton, M., Strong, R., Sun, E., Svirskas, R., Tector, C., Turner, R., Venter, E., Wang, A. H., Wang, X., Wang, Z. Y., Wassarman, D. A., Weinstock, G. M., Weissenbach, J., Williams, S. M., WoodageT, Worley, K. C., Wu, D., Yang, S., Yao, Q. A., Ye, J., Yeh, R. F., Zaveri, J. S., Zhan, M., Zhang, G., Zhao, Q., Zheng, L., Zheng, X. H., Zhong, F. N., Zhong, W., Zhou, X., Zhu, S., Zhu, X., Smith, H. O., Gibbs, R. A., Myers, E. W., Rubin, G. M., and Venter, J. C. (2000) The genome sequence of *Drosophila melanogaster*. *Science* **287**, 2185-2195

91. Yucel, J. K., Marszalek, J. D., McIntosh, J. R., Goldstein, L. S., Cleveland, D. W., and Philp, A. V. (2000) CENP-meta, an essential kinetochore kinesin required for the maintenance of metaphase chromosome alignment in *Drosophila*. *J Cell Biol* **150**, 1-11
92. Shi, W., Stampas, A., Zapata, C., and Baker, N. E. (2003) The pineapple eye gene is required for survival of *Drosophila* imaginal disc cells. *Genetics* **165**, 1869-1879
93. Hoskins, R. A., Carlson, J. W., Kennedy, C., Acevedo, D., Evans-Holm, M., Frise, E., Wan, K. H., Park, S., Mendez-Lago, M., Rossi, F., Villasante, A., Dimitri, P., Karpen, G. H., and Celniker, S. E. (2007) Sequence finishing and mapping of *Drosophila melanogaster* heterochromatin. *Science* **316**, 1625-1628
94. Yang, J. T., Laymon, R. A., and Goldstein, L. S. (1989) A three-domain structure of kinesin heavy chain revealed by DNA sequence and microtubule binding analyses. *Cell* **56**, 879-889
95. Heck, M. M., Pereira, A., Pesavento, P., Yannoni, Y., Spradling, A. C., and Goldstein, L. S. (1993) The kinesin-like protein KLP61F is essential for mitosis in *Drosophila*. *J Cell Biol* **123**, 665-679
96. Pesavento, P. A., Stewart, R. J., and Goldstein, L. S. (1994) Characterization of the KLP68D kinesin-like protein in *Drosophila*: possible roles in axonal transport. *J Cell Biol* **127**, 1041-1048
97. Sisson, J. C., Ho, K. S., Suyama, K., and Scott, M. P. (1997) Costal2, a novel kinesin-related protein in the Hedgehog signaling pathway. *Cell* **90**, 235-245
98. Richards, S., Liu, Y., Bettencourt, B. R., Hradecky, P., Letovsky, S., Nielsen, R., Thornton, K., Hubisz, M. J., Chen, R., Meisel, R. P., Couronne, O., Hua, S., Smith, M. A., Zhang, P., Liu, J., Bussemaker, H. J., van Batenburg, M. F., Howells, S. L., Scherer, S. E., Sodergren, E., Matthews, B. B., Crosby, M. A., Schroeder, A. J., Ortiz-Barrientos, D., Rives, C. M., Metzker, M. L., Muzny, D. M., Scott, G., Steffen, D., Wheeler, D. A., Worley, K. C., Havlak, P., Durbin, K. J., Egan, A., Gill, R., Hume, J., Morgan, M. B., Miner, G., Hamilton, C., Huang, Y., Waldron, L., Verduzco, D., Clerc-Blankenburg, K. P., Dubchak, I., Noor, M. A., Anderson, W., White, K. P., Clark, A. G., Schaeffer, S. W., Gelbart, W., Weinstock, G. M., and Gibbs, R. A. (2005) Comparative genome sequencing of *Drosophila pseudoobscura*: chromosomal, gene, and cis-element evolution. *Genome Res* **15**, 1-18
99. Requena, N., Alberti-Segui, C., Winzenburg, E., Horn, C., Schliwa, M., Philippsen, P., Liese, R., and Fischer, R. (2001) Genetic evidence for a microtubule-destabilizing effect of conventional kinesin and analysis of its consequences for the control of nuclear distribution in *Aspergillus nidulans*. *Mol Microbiol* **42**, 121-132
100. Rischitor, P. E., Konzack, S., and Fischer, R. (2004) The Kip3-like kinesin KipB moves along microtubules and determines spindle position during synchronized mitoses in *Aspergillus nidulans* hyphae. *Eukaryot Cell* **3**, 632-645
101. Enos, A. P., and Morris, N. R. (1990) Mutation of a gene that encodes a kinesin-like protein blocks nuclear division in *A. nidulans*. *Cell* **60**, 1019-1027

102. Wade, C. M., Giulotto, E., Sigurdsson, S., Zoli, M., Gnerre, S., Imsland, F., Lear, T. L., Adelson, D. L., Bailey, E., Bellone, R. R., Blocker, H., Distl, O., Edgar, R. C., Garber, M., Leeb, T., Mauceli, E., MacLeod, J. N., Penedo, M. C., Raison, J. M., Sharpe, T., Vogel, J., Andersson, L., Antczak, D. F., Biagi, T., Binns, M. M., Chowdhary, B. P., Coleman, S. J., Della Valle, G., Fryc, S., Guerin, G., Hasegawa, T., Hill, E. W., Jurka, J., Kiialainen, A., Lindgren, G., Liu, J., Magnani, E., Mickelson, J. R., Murray, J., Nergadze, S. G., Onofrio, R., Pedroni, S., Piras, M. F., Raudsepp, T., Rocchi, M., Roed, K. H., Ryder, O. A., Searle, S., Skow, L., Swinburne, J. E., Syvanen, A. C., Tozaki, T., Valberg, S. J., Vaudin, M., White, J. R., Zody, M. C., Lander, E. S., and Lindblad-Toh, K. (2009) Genome sequence, comparative analysis, and population genetics of the domestic horse. *Science* **326**, 865-867

103. Wang, S. Z., and Adler, R. (1994) A developmentally regulated basic-leucine zipper-like gene and its expression in embryonic retina and lens. *Proc Natl Acad Sci U.S.A.* **91**, 1351-1355

104. Caldwell, R. B., Kierzek, A. M., Arakawa, H., Bezzubov, Y., Zaim, J., Fiedler, P., Kutter, S., Blagodatski, A., Kostovska, D., Koter, M., Plachy, J., Carninci, P., Hayashizaki, Y., and Buerstedde, J. M. (2005) Full-length cDNAs from chicken bursal lymphocytes to facilitate gene function analysis. *Genome Biol* **6**, R6

105. Miller, M. M., Bacon, L. D., Hala, K., Hunt, H. D., Ewald, S. J., Kaufman, J., Zoorob, R., and Briles, W. E. (2004) 2004 Nomenclature for the chicken major histocompatibility (B and Y) complex. *Immunogenetics* **56**, 261-279

106. Schoch, C. L., Aist, J. R., Yoder, O. C., and Gillian Turgeon, B. (2003) A complete inventory of fungal kinesins in representative filamentous ascomycetes. *Fungal Genet Biol* **39**, 1-15

107. Xu, T., Sun, X., Jiang, S., Ren, D., and Liu, G. (2007) Cotton GhKCH2, a plant-specific kinesin, is low-affinitive and nucleotide-independent as binding to microtubule. *J Biochem Mol Biol* **40**, 723-730

108. Navone, F., Niclas, J., Hom-Booher, N., Sparks, L., Bernstein, H. D., McCaffrey, G., and Vale, R. D. (1992) Cloning and expression of a human kinesin heavy chain gene: interaction of the COOH-terminal domain with cytoplasmic microtubules in transfected CV-1 cells. *J Cell Biol* **117**, 1263-1275

109. Sardella, M., Navone, F., Rocchi, M., Rubartelli, A., Viggiano, L., Vignali, G., Consalez, G. G., Sitia, R., and Cabibbo, A. (1998) KIF3C, a novel member of the kinesin superfamily: sequence, expression, and mapping to human chromosome 2 at 2p23. *Genomics* **47**, 405-408

110. Nagase, T., Ishikawa, K., Nakajima, D., Ohira, M., Seki, N., Miyajima, N., Tanaka, A., Kotani, H., Nomura, N., and Ohara, O. (1997) Prediction of the coding sequences of unidentified human genes. VII. The complete sequences of 100 new cDNA clones from brain which can code for large proteins *in vitro*. *DNA Res* **4**, 141-150

111. Nagase, T., Ishikawa, K., Miyajima, N., Tanaka, A., Kotani, H., Nomura, N., and Ohara, O. (1998) Prediction of the coding sequences of unidentified human genes. IX. The complete sequences of 100 new cDNA clones from brain which can code for large proteins *in vitro*. *DNA Res* **5**, 31-39

112. Tokai, N., Fujimoto-Nishiyama, A., Toyoshima, Y., Yonemura, S., Tsukita, S., Inoue, J., and Yamamota, T. (1996) Kid, a novel kinesin-like DNA binding protein, is localized to chromosomes and the mitotic spindle. *EMBO J* **15**, 457-467

113. Janitz, K., Wild, A., Beck, S., Savasta, S., Beluffi, G., Ziegler, A., and Volz, A. (1999) Genomic organization of the HSET locus and the possible association of HLA-linked genes with immotile cilia syndrome (ICS). *Immunogenetics* **49**, 644-652

114. Okamoto, S., Matsushima, M., and Nakamura, Y. (1998) Identification, genomic organization, and alternative splicing of KNSL3, a novel human gene encoding a kinesin-like protein. *Cytogenet Cell Genet* **83**, 25-29

115. Kim, I. G., Jun, D. Y., Sohn, U., and Kim, Y. H. (1997) Cloning and expression of human mitotic centromere-associated kinesin gene. *Biochim Biophys Acta* **1359**, 181-186

116. Hanada, T., Lin, L., Tibaldi, E. V., Reinherz, E. L., and Chishti, A. H. (2000) GAKIN, a novel kinesin-like protein associates with the human homologue of the *Drosophila* discs large tumor suppressor in T lymphocytes. *J Biol Chem* **275**, 28774-28784

117. Nomura, N., Nagase, T., Miyajima, N., Sazuka, T., Tanaka, A., Sato, S., Seki, N., Kawarabayasi, Y., Ishikawa, K., and Tabata, S. (1994) Prediction of the coding sequences of unidentified human genes. II. The coding sequences of 40 new genes (KIAA0041-KIAA0080) deduced by analysis of cDNA clones from human cell line KG-1. *DNA Res* **1**, 223-229

118. Gerhard, D. S., Wagner, L., Feingold, E. A., Shenmen, C. M., Grouse, L. H., Schuler, G., Klein, S. L., Old, S., Rasooly, R., Good, P., Guyer, M., Peck, A. M., Derge, J. G., Lipman, D., Collins, F. S., Jang, W., Sherry, S., Feolo, M., Misquitta, L., Lee, E., Rotmistrovsky, K., Greenhut, S. F., Schaefer, C. F., Buetow, K., Bonner, T. I., Haussler, D., Kent, J., Kiekhaus, M., Furey, T., Brent, M., Prange, C., Schreiber, K., Shapiro, N., Bhat, N. K., Hopkins, R. F., Hsie, F., Driscoll, T., Soares, M. B., Casavant, T. L., Scheetz, T. E., Brown-stein, M. J., Usdin, T. B., Toshiyuki, S., Carninci, P., Piao, Y., Dudekula, D. B., Ko, M. S., Kawakami, K., Suzuki, Y., Sugano, S., Gruber, C. E., Smith, M. R., Simmons, B., Moore, T., Waterman, R., Johnson, S. L., Ruan, Y., Wei, C. L., Mathavan, S., Gunaratne, P. H., Wu, J., Garcia, A. M., Hulyk, S. W., Fuh, E., Yuan, Y., Sneed, A., Kowis, C., Hodgson, A., Muzny, D. M., McPherson, J., Gibbs, R. A., Fahey, J., Helton, E., Ketteman, M., Madan, A., Rodrigues, S., Sanchez, A., Whiting, M., Madari, A., Young, A. C., Wetherby, K. D., Granite, S. J., Kwong, P. N., Brinkley, C. P., Pearson, R. L., Bouffard, G. G., Blakesly, R. W., Green, E. D., Dickson, M. C., Rodriguez, A. C., Grimwood, J., Schmutz, J., Myers, R. M., Butterfield, Y. S., Griffith, M., Griffith, O. L., Krzywinski, M. I., Liao, N., Morin, R., Palmquist, D., Petrescu, A. S., Skalska, U., Smailus, D. E., Stott, J. M., Schnerch, A., Schein, J. E., Jones, S. J., Holt, R. A., Baross, A., Marra, M. A., Clifton, S., Makowski, K. A., Bosak, S., Malek, J., and MGC Project Team. (2004) The status, quality, and expansion of the NIH full-length cDNA project: the Mammalian Gene Collection (MGC). *Genome Res* **14**, 2121-2127

119. Gregory, S. G., Barlow, K. F., McLay, K. E., Kaul, R., Swarbreck, D., Dunham, A., Scott, C. E., Howe, K. L., Woodfine, K., Spencer, C. C., Jones, M. C., Gillson, C., Searle, S., Zhou, Y., Kokocinski, F., McDonald, L., Evans, R., Phillips, K., Atkinson, A., Cooper, R., Jones, C., Hall, R. E., Andrews, T. D., Lloyd, C., Ainscough, R., Almeida, J. P., Ambrose, K. D., Anderson, F., Andrew, R. W., Ashwell, R. I., Aubin, K., Babbage, A. K., Bagguley, C. L., Bailey, J., Beasley, H., Bethel, G., Bird, C. P., Bray-Allen, S., Brown, J. Y., Brown, A. J., Buckley, D., Burton, J., Bye, J., Carder, C., Chapman, J. C., Clark, S. Y., Clarke, G., Clee, C., Cobley, V., Collier, R. E., Corby, N., Coville, G. J., Davies, J., Deadman, R., Dunn, M., Earthrowl, M., Ellington, A. G., Errington, H., Frankish, A., Frankland, J., French, L., Garner, P., Garnett, J., Gay, L., Ghori, M. R., Gibson, R., Gilby, L. M., Gillett, W., Glithero, R. J., Grafham, D. V., Griffiths, C., Griffiths-Jones, S., Grocock, R., Hammond, S., Harrison, E. S., Hart, E., Haugen, E., Heath, P. D., Holmes, S., Holt, K., Howden, P. J., Hunt, A. R., Hunt, S. E., Hunter, G., Isherwood, J., James, R., Johnson, C., Johnson, D., Joy, A., Kay, M., Kershaw, J. K., Kibukawa, M., Kimberley, A. M., King, A., Knights, A. J., Lad, H., Laird, G., Lawlor, S., Leongamornlert, D. A., Lloyd, D. M., Loveland, J., Lovell, J., Lush, M. J., Lyne, R., Martin, S., Mashreghi-Mohammadi, M., Matthews, L., Matthews, N. S., McLaren, S., Milne, S., Mistry, S., Moore, M. J., Nickerson, T., O'Dell, C. N., Oliver, K., Palmeiri, A., Palmer, S. A., Parker, A., Patel, D., Pearce, A. V., Peck, A. I., Pelan, S., Phelps, K., Phillimore, B. J., Plumb, R., Rajan, J., Raymond, C., Rouse, G., Saenphimmachak, C., Sehra, H. K., Sheridan, E., Shownkeen, R., Sims, S., Skuce, C. D., Smith, M., Steward, C., Subramanian, S., Sycamore, N., Tracey, A., Tromans, A., Van Helmond, Z., Wall, M., Wallis, J. M., White, S., Whitehead, S. L., Wilkinson, J. E., Willey, D. L., Williams, H., Wilming, L., Wray, P. W., Wu, Z., Coulson, A., Vaudin, M., Sulston, J. E., Durbin, R., Hubbard, T., Wooster, R., Dunham, I., Carter, N. P., McVean, G., Ross, M. T., Harrow, J., Olson, M. V., Beck, S., Rogers, J., Bentley, D. R., Banerjee, R., Bryant, S. P., Burford, D. C., Burrill, W. D., Clegg, S. M., Dhami, P., Dovey, O., Faulkner, L. M., Gribble, S. M., Langford, C. F., Pandian, R. D.,

Porter, K. M., and Prigmore, E. (2006) The DNA sequence and biological annotation of human chromosome 1. *Nature* **441**, 315-321

120. Yamada, K., Andrews, C., Chan, W. M., McKeown, C. A., Magli, A., de Berardinis, T., Loewenstein, A., Lazar, M., O'Keefe, M., Letson, R., London, A., Ruttum, M., Matsumoto, N., Saito, N., Morris, L., Del Monte, M., Johnson, R. H., Uyama, E., Houtman, W. A., de Vries, B., Carlow, T. J., Hart, B. L., Krawiecki, N., Shoffner, J., Vogel, M. C., Katowitz, J., Goldstein, S. M., Levin, A. V., Sener, E. C., Ozturk, B. T., Akarsu, A. N., Brodsky, M. C., Hanisch, F., Cruse, R. P., Zubcov, A. A., Robb, R. M., Roggenkaemper, P., Gottlob, I., Kowal, L., Battu, R., Traboulsi, E. I., Franceschini, P., Newlin, A., Demer, J. L., and Engle, E. C. (2003) Heterozygous mutations of the kinesin KIF21A in congenital fibrosis of the extraocular muscles type 1 (CFEOM1). *Nat Genet* **35**, 318-321

121. Nagase, T., Kikuno, R., Nakayama, M., Hirosawa, M., and Ohara, O. (2000) Prediction of the coding sequences of unidentified human genes. XVIII. The complete sequences of 100 new cDNA clones from brain which code for large proteins *in vitro*. *DNA Res* **7**, 273-281

122. Seki, N., Ohira, M., Nagase, T., Ishikawa, K., Miyajima, N., Nakajima, D., Nomura, N., and Ohara, O. (1997) Characterization of cDNA clones in size-fractionated cDNA libraries from human brain. *DNA Res* **4**, 345-349

123. Luboshits, G., and Benayahu, D. (2005) MS-KIF18A, new kinesin; structure and cellular expression. *Gene* **351**, 19-28

124. Oduru, S., Campbell, J. L., Karri, S., Hendry, W. J., Khan, S. A., and Williams, S. C. (2003) Gene discovery in the hamster: a comparative genomics approach for gene annotation by sequencing of hamster testis cDNAs. *BMC genomics* **4**, 22

125. Sueishi, M., Takagi, M., and Yoneda, Y. (2000) The forkhead-associated domain of Ki-67 antigen interacts with the novel kinesin-like protein Hklp2. *J Biol Chem* **275**, 28888-28892

126. Humphray, S. J., Oliver, K., Hunt, A. R., Plumb, R. W., Loveland, J. E., Howe, K. L., Andrews, T. D., Searle, S., Hunt, S. E., Scott, C. E., Jones, M. C., Ainscough, R., Almeida, J. P., Ambrose, K. D., Ashwell, R. I., Babbage, A. K., Babbage, S., Bagguley, C. L., Bailey, J., Banerjee, R., Barker, D. J., Barlow, K. F., Bates, K., Beasley, H., Beasley, O., Bird, C. P., Bray-Allen, S., Brown, A. J., Brown, J. Y., Burford, D., Burrill, W., Burton, J., Carder, C., Carter, N. P., Chapman, J. C., Chen, Y., Clarke, G., Clark, S. Y., Clee, C. M., Clegg, S., Collier, R. E., Corby, N., Crosier, M., Cummings, A. T., Davies, J., Dhami, P., Dunn, M., Dutta, I., Dyer, L. W., Earthrowl, M. E., Faulkner, L., Fleming, C. J., Frankish, A., Frankland, J. A., French, L., Fricker, D. G., Garner, P., Garnett, J., Ghori, J., Gilbert, J. G., Glison, C., Grafham, D. V., Gribble, S., Griffiths, C., Griffiths-Jones, S., Grocock, R., Guy, J., Hall, R. E., Hammond, S., Harley, J. L., Harrison, E. S., Hart, E. A., Heath, P. D., Henderson, C. D., Hopkins, B. L., Howard, P. J., Howden, P. J., Huckle, E., Johnson, C., Johnson, D., Joy, A. A., Kay, M., Keenan, S., Kershaw, J. K., Kimberley, A. M., King, A., Knights, A., Laird, G. K., Langford, C., Lawlor, S., Leongamornlert, D. A., Leversha, M., Lloyd, C., Lloyd, D. M., Lovell, J., Martin, S., Mashreghi-Mohammadi, M., Matthews, L., McLaren, S., McLay, K. E., McMurray, A., Milne, S., Nickerson, T., Nisbett, J., Nordsiek, G., Pearce, A. V., Peck, A. I., Porter, K. M., Pandian, R., Pelan, S., Phillimore, B., Povey, S., Ramsey, Y., Rand, V., Scharfe, M., Sehra, H. K., Shownkeen, R., Sims, S. K., Skuce, C. D., Smith, M., Steward, C. A., Swarbreck, D., Sycamore, N., Tester, J., Thorpe, A., Tracey, A., Tromans, A., Thomas, D. W., Wall, M., Wallis, J. M., West, A. P., Whitehead, S. L., Willey, D. L., Williams, S. A., Wilming, L., Wray, P. W., Young, L., Ashurst, J. L., Coulson, A., Blocker, H., Durbin, R., Sulston, J. E., Hubbard, T., Jackson, M. J., Bentley, D. R., Beck, S., Rogers, J., and Dunham, I. (2004) DNA sequence and analysis of human chromosome 9. *Nature* **429**, 369-374

127. Blangy, A., Lane, H. A., d'Herin, P., Harper, M., Kress, M., and Nigg, E. A. (1995) Phosphorylation by p34cdc2 regulates spindle association of human Eg5, a kinesin-related motor essential for bipolar spindle formation *in vivo*. *Cell* **83**, 1159-1169

128. Nagai, M., Ichimiya, S., Ozaki, T., Seki, N., Mihara, M., Furuta, S., Ohira, M., Tomioka, N., Nomura, N., Sakiyama, S., Kubo, O., Takakura, K., Hori, T., and Nakagawara, A. (2000) Identification of the full-length KIAA0591 gene encoding a novel kinesin-related protein which is mapped to the neuroblastoma suppressor gene locus at 1p36.2. *Int J Oncol* **16**, 907-916
129. Dorner, C., Ciossek, T., Muller, S., Moller, P. H., Ullrich, A., and Lammers, R. (1998) Characterization of KIF1C, a new kinesin-like protein involved in vesicle transport from the Golgi apparatus to the endoplasmic reticulum. *J Biol Chem* **273**, 20267-20275
130. Jamain, S., Quach, H., Fellous, M., and Bourgeron, T. (2001) Identification of the human KIF13A gene homologous to *Drosophila* kinesin-73 and candidate for schizophrenia. *Genomics* **74**, 36-44
131. Furlong, R. A., Zhou, C. Y., Ferguson-Smith, M. A., and Affara, N. A. (1996) Characterization of a kinesin-related gene ATSV, within the tuberous sclerosis locus (TSC1) candidate region on chromosome 9Q34. *Genomics* **33**, 421-429
132. Hoang, E. H., Whitehead, J. L., Dose, A. C., and Burnside, B. (1998) Cloning of a novel C-terminal kinesin (KIFC3) that maps to human chromosome 16q13-q21 and thus is a candidate gene for Bardet-Biedl syndrome. *Genomics* **52**, 219-222
133. Niclas, J., Navone, F., Hom-Booher, N., and Vale, R. D. (1994) Cloning and localization of a conventional kinesin motor expressed exclusively in neurons. *Neuron* **12**, 1059-1072
134. Oh, S., Hahn, H., Torrey, T. A., Shin, H., Choi, W., Lee, Y. M., Morse, H. C., and Kim, W. (2000) Identification of the human homologue of mouse KIF4, a kinesin superfamily motor protein. *Biochim Biophys Acta* **1493**, 219-224
135. Debernardi, S., Fontanella, E., De Gregorio, L., Pierotti, M. A., and Delia, D. (1997) Identification of a novel human kinesin-related gene (HK2) by the cDNA differential display technique. *Genomics* **42**, 67-73
136. Heilig, R., Eckenberg, R., Petit, J. L., Fonknechten, N., Da Silva, C., Cattolico, L., Levy, M., Barbe, V., de Berardinis, V., Ureta-Vidal, A., Pelletier, E., Vico, V., Anthouard, V., Rowen, L., Madan, A., Qin, S., Sun, H., Du, H., Pepin, K., Artiguenave, F., Robert, C., Cruaud, C., Bruls, T., Jaillon, O., Friedlander, L., Samson, G., Brottier, P., Cure, S., Segurens, B., Aniere, F., Samain, S., Crespeau, H., Abbasi, N., Aiach, N., Boscus, D., Dickhoff, R., Dors, M., Dubois, I., Friedman, C., Gouyvenoux, M., James, R., Mairey-Estrada, B., Mangenot, S., Martins, N., Menard, M., Oztas, S., Ratcliffe, A., Shaffer, T., Trask, B., Vacherie, B., Bellemere, C., Belser, C., Besnard-Gonnet, M., Bartol-Mavel, D., Boutard, M., Briez-Silla, S., Combette, S., Dufosse-Laurent, V., Ferron, C., Lechaplais, C., Louesse, C., Muselet, D., Magdelenat, G., Pateau, E., Petit, E., Sirvain-Trukniewicz, P., Trybou, A., Vega-Czarny, N., Bataille, E., Bluet, E., Bordelais, I., Dubois, M., Dumont, C., Guerin, T., Haffray, S., Hammadi, R., Muanga, J., Pellouin, V., Robert, D., Wunderle, E., Gauguet, G., Roy, A., Sainte-Marthe, L., Verdier, J., Verdier-Discala, C., Hillier, L., Fulton, L., McPherson, J., Matsuda, F., Wilson, R., Scarpelli, C., Gyapay, G., Wincker, P., Saurin, W., Quetier, F., Waterston, R., Hood, L., and Weissenbach, J. (2003) The DNA sequence and analysis of human chromosome 14. *Nature* **421**, 601-607
137. Nagase, T., Kikuno, R., Ishikawa, K. I., Hirosawa, M., and Ohara, O. (2000) Prediction of the coding sequences of unidentified human genes. XVI. The complete sequences of 150 new cDNA clones from brain which code for large proteins *in vitro*. *DNA Res* **7**, 65-73
138. Yen, T. J., Li, G., Schaar, B. T., Szilak, I., and Cleveland, D. W. (1992) CENP-E is a putative kinetochore motor that accumulates just before mitosis. *Nature* **359**, 536-539
139. Clark, H. F., Gurney, A. L., Abaya, E., Baker, K., Baldwin, D., Brush, J., Chen, J., Chow, B., Chui, C., Crowley, C., Currell, B., Deuel, B., Dowd, P., Eaton, D., Foster, J., Grimaldi, C., Gu, Q., Hass, P. E., Heldens, S., Huang, A., Kim, H. S., Klimowski, L., Jin, Y., Johnson, S., Lee, J., Lewis, L., Liao, D., Mark, M., Robbie, E., Sanchez, C., Schoenfeld, J., Seshagiri, S., Simmons, L., Singh, J., Smith, V., Stinson, J., Vagts, A., Vandlen, R., Watanabe, C., Wieand, D., Woods, K., Xie, M. H., Yansura, D., Yi, S., Yu, G., Yuan, J., Zhang, M., Zhang, Z., Goddard, A., Wood, W. I., Godowski, P., and Gray, A. (2003) The secreted protein discovery initiative (SPDI), a

large-scale effort to identify novel human secreted and transmembrane proteins: a bioinformatics assessment. *Genome Res* **13**, 2265-2270

140. Whitehead, J. L., Wang, S. Y., Bost-Usinger, L., Hoang, E., Frazer, K. A., and Burnside, B. (1999) Photoreceptor localization of the KIF3A and KIF3B subunits of the heterotrimeric microtubule motor kinesin II in vertebrate retina. *Exp Eye Res* **69**, 491-503
141. Peacock, C. S., Seeger, K., Harris, D., Murphy, L., Ruiz, J. C., Quail, M. A., Peters, N., Adlem, E., Tivey, A., Aslett, M., Kerhornou, A., Ivens, A., Fraser, A., Rajandream, M. A., Carver, T., Norbertczak, H., Chillingworth, T., Hance, Z., Jagels, K., Moule, S., Ormond, D., Rutter, S., Squares, R., Whitehead, S., Rabbinowitsch, E., Arrowsmith, C., White, B., Thurston, S., Bringaud, F., Baldauf, S. L., Faulconbridge, A., Jeffares, D., Depledge, D. P., Oyola, S. O., Hilley, J. D., Brito, L. O., Tosi, L. R., Barrell, B., Cruz, A. K., Mottram, J. C., Smith, D. F., and Berriman, M. (2007) Comparative genomic analysis of three *Leishmania* species that cause diverse human disease. *Nat Genet* **39**, 839-847
142. Burns, J. M., Jr., Shreffler, W. G., Benson, D. R., Ghalib, H. W., Badaro, R., and Reed, S. G. (1993) Molecular characterization of a kinesin-related antigen of *Leishmania chagasi* that detects specific antibody in African and American visceral leishmaniasis. *Proc Natl Acad Sci U.S.A.* **90**, 775-779
143. Gerald, N. J., Coppens, I., and Dwyer, D. M. (2007) Molecular dissection and expression of the LdK39 kinesin in the human pathogen, *Leishmania donovani*. *Mol Microbiol* **63**, 962-979
144. Ivens, A. C., Lewis, S. M., Bagherzadeh, A., Zhang, L., Chan, H. M., and Smith, D. F. (1998) A physical map of the *Leishmania major* Friedlin genome. *Genome Res* **8**, 135-145
145. Myler, P. J., Audleman, L., deVos, T., Hixson, G., Kiser, P., Lemley, C., Magness, C., Rickel, E., Sisk, E., Sunkin, S., Swartzell, S., Westlake, T., Bastien, P., Fu, G., Ivens, A., and Stuart, K. (1999) *Leishmania major* Friedlin chromosome 1 has an unusual distribution of protein-coding genes. *Proc Natl Acad Sci U.S.A.* **96**, 2902-2906
146. Butler, G., Rasmussen, M. D., Lin, M. F., Santos, M. A., Sakthikumar, S., Munro, C. A., Rheinbay, E., Grabherr, M., Forche, A., Reedy, J. L., Agrafioti, I., Arnaud, M. B., Bates, S., Brown, A. J., Brunke, S., Costanzo, M. C., Fitzpatrick, D. A., de Groot, P. W., Harris, D., Hoyer, L. L., Hube, B., Klis, F. M., Kodira, C., Lennard, N., Logue, M. E., Martin, R., Neiman, A. M., Nikolaou, E., Quail, M. A., Quinn, J., Santos, M. C., Schmitzberger, F. F., Sherlock, G., Shah, P., Silverstein, K. A., Skrzypek, M. S., Soll, D., Staggs, R., Stansfield, I., Stumpf, M. P., Sudbery, P. E., Srikantha, T., Zeng, Q., Berman, J., Berriman, M., Heitman, J., Gow, N. A., Lorenz, M. C., Birren, B. W., Kellis, M., and Cuomo, C. A. (2009) Evolution of pathogenicity and sexual reproduction in eight *Candida* genomes. *Nature* **459**, 657-662
147. Kosik, K. S., Orecchio, L. D., Schnapp, B., Inouye, H., and Neve, R. L. (1990) The primary structure and analysis of the squid kinesin heavy chain. *J Biol Chem* **265**, 3278-3283
148. Osada, N., Hida, M., Kusuda, J., Tanuma, R., Hirata, M., Suto, Y., Hirai, M., Terao, K., Sugano, S., and Hashimoto, K. (2002) Cynomolgus monkey testicular cDNAs for discovery of novel human genes in the human genome sequence. *BMC genomics* **3**, 36
149. Osada, N., Hirata, M., Tanuma, R., Kusuda, J., Hida, M., Suzuki, Y., Sugano, S., Gojobori, T., Shen, C. K., Wu, C. I., and Hashimoto, K. (2005) Substitution rate and structural divergence of 5'UTR evolution: comparative analysis between human and cynomolgus monkey cDNAs. *Mol Biol Evol* **22**, 1976-1982
150. Rhesus Macaque Genome Sequencing and Analysis Consortium, Gibbs, R. A., Rogers, J., Katze, M. G., Bumgarner, R., Weinstock, G. M., Mardis, E. R., Remington, K. A., Strausberg, R. L., Venter, J. C., Wilson, R. K., Batzer, M. A., Bustamante, C. D., Eichler, E. E., Hahn, M. W., Hardison, R. C., Makova, K. D., Miller, W., Milosavljevic, A., Palermo, R. E., Siepel, A., Sikela, J. M., Attaway, T., Bell, S., Bernard, K. E., Buhay, C. J., Chandrabose, M. N., Dao, M., Davis, C., Delehaunty, K. D., Ding, Y., Dinh, H. H., Dugan-Rocha, S., Fulton, L. A., Gabisi, R. A., Garner, T. T., Godfrey, J., Hawes, A. C., Hernandez, J., Hines, S., Holder, M., Hume, J., Jhangiani, S. N., Joshi, V., Khan, Z. M., Kirkness, E. F., Cree, A., Fowler, R. G., Lee, S., Lewis,

L. R., Li, Z., Liu, Y. S., Moore, S. M., Muzny, D., Nazareth, L. V., Ngo, D. N., Okwuonu, G. O., Pai, G., Parker, D., Paul, H. A., Pfannkoch, C., Pohl, C. S., Rogers, Y. H., Ruiz, S. J., Sabo, A., Santibanez, J., Schneider, B. W., Smith, S. M., Sodergren, E., Svatek, A. F., Utterback, T. R., Vattathil, S., Warren, W., White, C. S., Chinwalla, A. T., Feng, Y., Halpern, A. L., Hillier, L. W., Huang, X., Minx, P., Nelson, J. O., Pepin, K. H., Qin, X., Sutton, G. G., Venter, E., Walenz, B. P., Wallis, J. W., Worley, K. C., Yang, S. P., Jones, S. M., Marra, M. A., Rocchi, M., Schein, J. E., Baertsch, R., Clarke, L., Csuros, M., Glasscock, J., Harris, R. A., Havlak, P., Jackson, A. R., Jiang, H., Liu, Y., Messina, D. N., Shen, Y., Song, H. X., Wylie, T., Zhang, L., Birney, E., Han, K., Konkel, M. K., Lee, J., Smit, A. F., Ullmer, B., Wang, H., Xing, J., Burhans, R., Cheng, Z., Karro, J. E., Ma, J., Raney, B., She, X., Cox, M. J., Demuth, J. P., Dumas, L. J., Han, S. G., Hopkins, J., Karimpour-Fard, A., Kim, Y. H., Pollack, J. R., Vinar, T., Addo-Quaye, C., Degenhardt, J., Denby, A., Hubisz, M. J., Indap, A., Kosiol, C., Lahn, B. T., Lawson, H. A., Marklein, A., Nielsen, R., Vallender, E. J., Clark, A. G., Ferguson, B., Hernandez, R. D., Hirani, K., Kehrer-Sawatzki, H., Kolb, J., Patil, S., Pu, L. L., Ren, Y., Smith, D. G., Wheeler, D. A., Schenck, I., Ball, E. V., Chen, R., Cooper, D. N., Giardine, B., Hsu, F., Kent, W. J., Lesk, A., Nelson, D. L., O'Brien W, E., Prufer, K., Stenson, P. D., Wallace, J. C., Ke, H., Liu, X. M., Wang, P., Xiang, A. P., Yang, F., Barber, G. P., Haussler, D., Karolchik, D., Kern, A. D., Kuhn, R. M., Smith, K. E., and Zwieg, A. S. (2007) Evolutionary and biomedical insights from the *rhesus macaque* genome. *Science* **316**, 222-234

151. Dean, R. A., Talbot, N. J., Ebbole, D. J., Farman, M. L., Mitchell, T. K., Orbach, M. J., Thon, M., Kulkarni, R., Xu, J. R., Pan, H., Read, N. D., Lee, Y. H., Carbone, I., Brown, D., Oh, Y. Y., Donofrio, N., Jeong, J. S., Soanes, D. M., Djonovic, S., Kolomiets, E., Rehmeyer, C., Li, W., Harding, M., Kim, S., Lebrun, M. H., Bohnert, H., Coughlan, S., Butler, J., Calvo, S., Ma, L. J., Nicol, R., Purcell, S., Nusbaum, C., Galagan, J. E., and Birren, B. W. (2005) The genome sequence of the rice blast fungus *Magnaporthe grisea*. *Nature* **434**, 980-986

152. Cannon, S. B., Sterck, L., Rombauts, S., Sato, S., Cheung, F., Gouzy, J., Wang, X., Mudge, J., Vasdewani, J., Schiex, T., Spannagl, M., Monaghan, E., Nicholson, C., Humphray, S. J., Schoof, H., Mayer, K. F., Rogers, J., Quetier, F., Oldroyd, G. E., Debelle, F., Cook, D. R., Retzel, E. F., Roe, B. A., Town, C. D., Tabata, S., Van de Peer, Y., and Young, N. D. (2006) Legume genome evolution viewed through the *Medicago truncatula* and *Lotus japonicus* genomes. *Proc Natl Acad Sci U.S.A.* **103**, 14959-14964

153. Mikkelsen, T. S., Wakefield, M. J., Aken, B., Amemiya, C. T., Chang, J. L., Duke, S., Garber, M., Gentles, A. J., Goodstadt, L., Heger, A., Jurka, J., Kamal, M., Mauceli, E., Searle, S. M., Sharpe, T., Baker, M. L., Batzer, M. A., Benos, P. V., Belov, K., Clamp, M., Cook, A., Cuff, J., Das, R., Davidow, L., Deakin, J. E., Fazzari, M. J., Glass, J. L., Grabherr, M., Greally, J. M., Gu, W., Hore, T. A., Huttley, G. A., Kleber, M., Jirtle, R. L., Koina, E., Lee, J. T., Mahony, S., Marra, M. A., Miller, R. D., Nicholls, R. D., Oda, M., Papenfuss, A. T., Parra, Z. E., Pollock, D. D., Ray, D. A., Schein, J. E., Speed, T. P., Thompson, K., VandeBerg, J. L., Wade, C. M., Walker, J. A., Waters, P. D., Webber, C., Weidman, J. R., Xie, X., Zody, M. C., Graves, J. A., Ponting, C. P., Breen, M., Samollow, P. B., Lander, E. S., and Lindblad-Toh, K. (2007) Genome of the marsupial *Monodelphis domestica* reveals innovation in non-coding sequences. *Nature* **447**, 167-177

154. Bost-Usinger, L., Chen, R. J., Hillman, D., Park, H., and Burnside, B. (1997) Multiple kinesin family members expressed in teleost retina and RPE include a novel C-terminal kinesin. *Exp Eye Res* **64**, 781-794

155. Aizawa, H., Sekine, Y., Takemura, R., Zhang, Z., Nangaku, M., and Hirokawa, N. (1992) Kinesin family in murine central nervous system. *J Cell Biol* **119**, 1287-1296

156. Sekine, Y., Okada, Y., Noda, Y., Kondo, S., Aizawa, H., Takemura, R., and Hirokawa, N. (1994) A novel microtubule-based motor protein (KIF4) for organelle transports, whose expression is regulated developmentally. *J Cell Biol* **127**, 187-201

157. Saito, N., Okada, Y., Noda, Y., Kinoshita, Y., Kondo, S., and Hirokawa, N. (1997) KIFC2 is a novel neuron-specific C-terminal type kinesin superfamily motor for dendritic transport of multivesicular body-like organelles. *Neuron* **18**, 425-438
158. Meng, Y. X., Wilson, G. W., Avery, M. C., Varden, C. H., and Balczon, R. (1997) Suppression of the expression of a pancreatic beta-cell form of the kinesin heavy chain by antisense oligonucleotides inhibits insulin secretion from primary cultures of mouse beta-cells. *Endocrinology* **138**, 1979-1987
159. Nitta, R., Okada, Y., and Hirokawa, N. (2008) Structural model for strain-dependent microtubule activation of Mg-ADP release from kinesin. *Nat Struct Mol Biol* **15**, 1067-1075
160. Yamazaki, H., Nakata, T., Okada, Y., and Hirokawa, N. (1995) KIF3A/B: a heterodimeric kinesin superfamily protein that works as a microtubule plus end-directed motor for membrane organelle transport. *J Cell Biol* **130**, 1387-1399
161. Yang, Z., and Goldstein, L. S. (1998) Characterization of the KIF3C neural kinesin-like motor from mouse. *Mol Biol Cell* **9**, 249-261
162. Navolanic, P. M., and Sperry, A. O. (2000) Identification of isoforms of a mitotic motor in mammalian spermatogenesis. *Biol Reprod* **62**, 1360-1369
163. Kato, K. (1990) A collection of cDNA clones with specific expression patterns in mouse brain. *Eur J Neurosci* **2**, 704-711
164. Nangaku, M., Sato-Yoshitake, R., Okada, Y., Noda, Y., Takemura, R., Yamazaki, H., and Hirokawa, N. (1994) KIF1B, a novel microtubule plus end-directed monomeric motor protein for transport of mitochondria. *Cell* **79**, 1209-1220
165. Nakagawa, T., Tanaka, Y., Matsuoka, E., Kondo, S., Okada, Y., Noda, Y., Kanai, Y., and Hirokawa, N. (1997) Identification and classification of 16 new kinesin superfamily (KIF) proteins in mouse genome. *Proc Natl Acad Sci U.S.A.* **94**, 9654-9659
166. Nakagawa, T., Setou, M., Seog, D., Ogasawara, K., Dohmae, N., Takio, K., and Hirokawa, N. (2000) A novel motor, KIF13A, transports mannose-6-phosphate receptor to plasma membrane through direct interaction with AP-1 complex. *Cell* **103**, 569-581
167. Setou, M., Nakagawa, T., Seog, D. H., and Hirokawa, N. (2000) Kinesin superfamily motor protein KIF17 and mLin-10 in NMDA receptor-containing vesicle transport. *Science* **288**, 1796-1802
168. Carninci, P., Kasukawa, T., Katayama, S., Gough, J., Frith, M. C., Maeda, N., Oyama, R., Ravasi, T., Lenhard, B., Wells, C., Kodzius, R., Shimokawa, K., Bajic, V. B., Brenner, S. E., Batalov, S., Forrest, A. R., Zavolan, M., Davis, M. J., Wilming, L. G., Aidinis, V., Allen, J. E., Ambesi-Impiombato, A., Apweiler, R., Aturaliya, R. N., Bailey, T. L., Bansal, M., Baxter, L., Beisel, K. W., Bersano, T., Bono, H., Chalk, A. M., Chiu, K. P., Choudhary, V., Christoffels, A., Clutterbuck, D. R., Crowe, M. L., Dalla, E., Dalrymple, B. P., de Bono, B., Della Gatta, G., di Bernardo, D., Down, T., Engstrom, P., Fagiolini, M., Faulkner, G., Fletcher, C. F., Fukushima, T., Furuno, M., Futaki, S., Gariboldi, M., Georgii-Hemming, P., Gingeras, T. R., Gojobori, T., Green, R. E., Gustincich, S., Harbers, M., Hayashi, Y., Hensch, T. K., Hirokawa, N., Hill, D., Huminiecki, L., Iacono, M., Ikeo, K., Iwama, A., Ishikawa, T., Jakt, M., Kanapin, A., Katoh, M., Kawasawa, Y., Kelso, J., Kitamura, H., Kitano, H., Kollias, G., Krishnan, S. P., Kruger, A., Kummerfeld, S. K., Kurochkin, I. V., Lareau, L. F., Lazarevic, D., Lipovich, L., Liu, J., Liuni, S., McWilliam, S., Madan Babu, M., Madera, M., Marchionni, L., Matsuda, H., Matsuzawa, S., Miki, H., Mignone, F., Miyake, S., Morris, K., Mottagui-Tabar, S., Mulder, N., Nakano, N., Nakauchi, H., Ng, P., Nilsson, R., Nishiguchi, S., Nishikawa, S., Nori, F., Ohara, O., Okazaki, Y., Orlando, V., Pang, K. C., Pavan, W. J., Pavesi, G., Pesole, G., Petrovsky, N., Piazza, S., Reed, J., Reid, J. F., Ring, B. Z., Ringwald, M., Rost, B., Ruan, Y., Salzberg, S. L., Sandelin, A., Schneider, C., Schonbach, C., Sekiguchi, K., Semple, C. A., Seno, S., Sessa, L., Sheng, Y., Shibata, Y., Shimada, H., Shimada, K., Silva, D., Sinclair, B., Sperling, S., Stupka, E., Sugiura, K., Sultana, R., Takenaka, Y., Taki, K., Tammoja, K., Tan, S. L., Tang, S., Taylor, M. S., Tegner, J., Teichmann, S. A., Ueda, H. R., van Nimwegen, E., Verardo, R., Wei, C. L., Yagi, K.,

Yamanishi, H., Zabarovsky, E., Zhu, S., Zimmer, A., Hide, W., Bult, C., Grimmond, S. M., Teasdale, R. D., Liu, E. T., Brusic, V., Quackenbush, J., Wahlestedt, C., Mattick, J. S., Hume, D. A., Kai, C., Sasaki, D., Tomaru, Y., Fukuda, S., Kanamori-Katayama, M., Suzuki, M., Aoki, J., Arakawa, T., Iida, J., Imamura, K., Itoh, M., Kato, T., Kawaji, H., Kawagashira, N., Kawashima, T., Kojima, M., Kondo, S., Konno, H., Nakano, K., Ninomiya, N., Nishio, T., Okada, M., Plessy, C., Shibata, K., Shiraki, T., Suzuki, S., Tagami, M., Waki, K., Watahiki, A., Okamura-Oho, Y., Suzuki, H., Kawai, J., Hayashizaki, Y., FANTOM Consortium, Group, Riken Genome Exploration Research Group and Genome Science Group (Genome Network Project Core Group). (2005) The transcriptional landscape of the mammalian genome. *Science* **309**, 1559-1563

169. Marszalek, J. R., Weiner, J. A., Farlow, S. J., Chun, J., and Goldstein, L. S. (1999) Novel dendritic kinesin sorting identified by different process targeting of two related kinesins: KIF21A and KIF21B. *J Cell Biol* **145**, 469-479

170. Buster, D. W., Baird, D. H., Yu, W., Solowska, J. M., Chauviere, M., Mazurek, A., Kress, M., and Baas, P. W. (2003) Expression of the mitotic kinesin Kif15 in postmitotic neurons: implications for neuronal migration and development. *J Neurocytol* **32**, 79-96

171. Weaver, B. A., Bonday, Z. Q., Putkey, F. R., Kops, G. J., Silk, A. D., and Cleveland, D. W. (2003) Centromere-associated protein-E is essential for the mammalian mitotic checkpoint to prevent aneuploidy due to single chromosome loss. *J Cell Biol* **162**, 551-563

172. Xia, C., Rahman, A., Yang, Z., and Goldstein, L. S. (1998) Chromosomal localization reveals three kinesin heavy chain genes in mouse. *Genomics* **52**, 209-213

173. Noda, Y., Okada, Y., Saito, N., Setou, M., Xu, Y., Zhang, Z., and Hirokawa, N. (2001) KIFC3, a microtubule minus end-directed motor for the apical transport of annexin XIIIb-associated Triton-insoluble membranes. *J Cell Biol* **155**, 77-88

174. Miki, H., Setou, M., Kaneshiro, K., and Hirokawa, N. (2001) All kinesin superfamily protein, KIF, genes in mouse and human. *Proc Natl Acad Sci U.S.A.* **98**, 7004-7011

175. Wu, Q., Sandrock, T. M., Turgeon, B. G., Yoder, O. C., Wirsel, S. G., and Aist, J. R. (1998) A fungal kinesin required for organelle motility, hyphal growth, and morphogenesis. *Mol Biol Cell* **9**, 89-101

176. Steinberg, G., and Schliwa, M. (1995) The *Neurospora* organelle motor: a distant relative of conventional kinesin with unconventional properties. *Mol Biol Cell* **6**, 1605-1618

177. Wang, W., Takezawa, D., Narasimhulu, S. B., Reddy, A. S., and Poovaiah, B. W. (1996) A novel kinesin-like protein with a calmodulin-binding domain. *Plant Mol Biol* **31**, 87-100

178. Asada, T., Kuriyama, R., and Shibaoka, H. (1997) TKRP125, a kinesin-related protein involved in the centrosome-independent organization of the cytokinetic apparatus in tobacco BY-2 cells. *J Cell Sci* **110** (Pt 2), 179-189

179. Warren, W. C., Hillier, L. W., Marshall Graves, J. A., Birney, E., Ponting, C. P., Grutzner, F., Belov, K., Miller, W., Clarke, L., Chinwalla, A. T., Yang, S. P., Heger, A., Locke, D. P., Miethke, P., Waters, P. D., Veyrunes, F., Fulton, L., Fulton, B., Graves, T., Wallis, J., Puente, X. S., Lopez-Otin, C., Ordonez, G. R., Eichler, E. E., Chen, L., Cheng, Z., Deakin, J. E., Alsop, A., Thompson, K., Kirby, P., Papenfuss, A. T., Wakefield, M. J., Olender, T., Lancet, D., Huttley, G. A., Smit, A. F., Pask, A., Temple-Smith, P., Batzer, M. A., Walker, J. A., Konkel, M. K., Harris, R. S., Whittington, C. M., Wong, E. S., Gemmell, N. J., Buschiazzo, E., Vargas Jentzsch, I. M., Merkel, A., Schmitz, J., Zemann, A., Churakov, G., Kriegs, J. O., Brosius, J., Murchison, E. P., Sachidanandam, R., Smith, C., Hannon, G. J., Tsend-Ayush, E., McMillan, D., Attenborough, R., Rens, W., Ferguson-Smith, M., Lefevre, C. M., Sharp, J. A., Nicholas, K. R., Ray, D. A., Kube, M., Reinhardt, R., Pringle, T. H., Taylor, J., Jones, R. C., Nixon, B., Dacheux, J. L., Niwa, H., Sekita, Y., Huang, X., Stark, A., Kheradpour, P., Kellis, M., Flicek, P., Chen, Y., Webber, C., Hardison, R., Nelson, J., Hallsworth-Pepin, K., Delehaunty, K., Markovic, C., Minx, P., Feng, Y., Kremitzki, C., Mitreva, M., Glasscock, J., Wylie, T., Wohldmann, P., Thiru, P., Nhan, M. N., Pohl, C. S., Smith, S. M., Hou, S., Nefedov, M., de Jong, P. J., Renfree, M. B., Mardis, E. R., and

Wilson, R. K. (2008) Genome analysis of the platypus reveals unique signatures of evolution. *Nature* **453**, 175-183

180. International Rice Genome Sequencing Project (2005) The map-based sequence of the rice genome. *Nature* **436**, 793-800

181. Buell, C. R., Yuan, Q., Ouyang, S., Liu, J., Zhu, W., Wang, A., Maiti, R., Haas, B., Wortman, J., Pertea, M., Jones, K. M., Kim, M., Overton, L., Tsitrin, T., Fadrosh, D., Bera, J., Weaver, B., Jin, S., Johri, S., Reardon, M., Webb, K., Hill, J., Moffat, K., Tallon, L., Van Aken, S., Lewis, M., Utterback, T., Feldblyum, T., Zismann, V., Iobst, S., Hsiao, J., de Vazeille, A. R., Salzberg, S. L., White, O., Fraser, C., Yu, Y., Kim, H., Rambo, T., Currie, J., Collura, K., Kernodle-Thompson, S., Wei, F., Kudrna, K., Ammiraju, J. S., Luo, M., Goicoechea, J. L., Wing, R. A., Henry, D., Oates, R., Palmer, M., Pries, G., Saski, C., Simmons, J., Soderlund, C., Nelson, W., de la Bastide, M., Spiegel, L., Nascimento, L., Huang, E., Preston, R., Zutavern, T., Palmer, L., O'Shaughnessy, A., Dike, S., McCombie, W. R., Minx, P., Cordum, H., Wilson, R., Jin, W., Lee, H. R., Jiang, J., Jackson, S., and Rice Chromosome 3 Sequencing Consortium. (2005) Sequence, annotation, and analysis of synteny between rice chromosome 3 and diverged grass species. *Genome Res* **15**, 1284-1291

182. Ohyanagi, H., Tanaka, T., Sakai, H., Shigemoto, Y., Yamaguchi, K., Habara, T., Fujii, Y., Antonio, B. A., Nagamura, Y., Imanishi, T., Ikeo, K., Itoh, T., Gojobori, T., and Sasaki, T. (2006) The Rice Annotation Project Database (RAP-DB): hub for *Oryza sativa* ssp. japonica genome information. *Nucleic Acids Res* **34**, D741-744

183. Feng, Q., Zhang, Y., Hao, P., Wang, S., Fu, G., Huang, Y., Li, Y., Zhu, J., Liu, Y., Hu, X., Jia, P., Zhao, Q., Ying, K., Yu, S., Tang, Y., Weng, Q., Zhang, L., Lu, Y., Mu, J., Zhang, L. S., Yu, Z., Fan, D., Liu, X., Lu, T., Li, C., Wu, Y., Sun, T., Lei, H., Li, T., Hu, H., Guan, J., Wu, M., Zhang, R., Zhou, B., Chen, Z., Chen, L., Jin, Z., Wang, R., Yin, H., Cai, Z., Ren, S., Lv, G., Gu, W., Zhu, G., Tu, Y., Jia, J., Chen, J., Kang, H., Chen, X., Shao, C., Sun, Y., Hu, Q., Zhang, X., Zhang, W., Wang, L., Ding, C., Sheng, H., Gu, J., Chen, S., Ni, L., Zhu, F., Chen, W., Lan, L., Lai, Y., Cheng, Z., Gu, M., Jiang, J., Li, J., Hong, G., Xue, Y., and Han, B. (2002) Sequence and analysis of rice chromosome 4. *Nature* **420**, 316-320

184. Yu, J., Wang, J., Lin, W., Li, S., Li, H., Zhou, J., Ni, P., Dong, W., Hu, S., Zeng, C., Zhang, J., Zhang, Y., Li, R., Xu, Z., Li, X., Zheng, H., Cong, L., Lin, L., Yin, J., Geng, J., Li, G., Shi, J., Liu, J., Lv, H., Li, J., Deng, Y., Ran, L., Shi, X., Wang, X., Wu, Q., Li, C., Ren, X., Li, D., Liu, D., Zhang, X., Ji, Z., Zhao, W., Sun, Y., Zhang, Z., Bao, J., Han, Y., Dong, L., Ji, J., Chen, P., Wu, S., Xiao, Y., Bu, D., Tan, J., Yang, L., Ye, C., Xu, J., Zhou, Y., Yu, Y., Zhang, B., Zhuang, S., Wei, H., Liu, B., Lei, M., Yu, H., Li, Y., Xu, H., Wei, S., He, X., Fang, L., Huang, X., Su, Z., Tong, W., Tong, Z., Ye, J., Wang, L., Lei, T., Chen, C., Chen, H., Huang, H., Zhang, F., Li, N., Zhao, C., Huang, Y., Li, L., Xi, Y., Qi, Q., Li, W., Hu, W., Tian, X., Jiao, Y., Liang, X., Jin, J., Gao, L., Zheng, W., Hao, B., Liu, S., Wang, W., Yuan, L., Cao, M., McDermott, J., Samudrala, R., Wong, G. K., and Yang, H. (2005) The Genomes of *Oryza sativa*: a history of duplications. *PLoS Biol* **3**, e38

185. Palenik, B., Grimwood, J., Aerts, A., Rouze, P., Salamov, A., Putnam, N., Dupont, C., Jorgensen, R., Derelle, E., Rombauts, S., Zhou, K., Otillar, R., Merchant, S. S., Podell, S., Gaasterland, T., Napoli, C., Gendler, K., Manuell, A., Tai, V., Vallon, O., Piganeau, G., Jancek, S., Heijde, M., Jabbari, K., Bowler, C., Lohr, M., Robbens, S., Werner, G., Dubchak, I., Pazour, G. J., Ren, Q., Paulsen, I., Delwiche, C., Schmutz, J., Rokhsar, D., Van de Peer, Y., Moreau, H., and Grigoriev, I. V. (2007) The tiny eukaryote *Ostreococcus* provides genomic insights into the paradox of plankton speciation. *Proc Natl Acad Sci U S A* **104**, 7705-7710

186. Derelle, E., Ferraz, C., Rombauts, S., Rouze, P., Worden, A. Z., Robbens, S., Partensky, F., Degroeve, S., Echeynie, S., Cooke, R., Saeys, Y., Wuyts, J., Jabbari, K., Bowler, C., Panaud, O., Piegu, B., Ball, S. G., Ral, J. P., Bouget, F. Y., Piganeau, G., De Baets, B., Picard, A., Delseny, M., Demaille, J., Van de Peer, Y., and Moreau, H. (2006) Genome analysis of the smallest free-

living eukaryote *Ostreococcus tauri* unveils many unique features. *Proc Natl Acad Sci U.S A.* **103**, 11647-11652
187. The Chimpanzee Sequencing and Analysis Consortium. (2005) Initial sequence of the chimpanzee genome and comparison with the human genome. *Nature* **437**, 69-87
188. Touitou, I., Lhomond, G., and Pruliere, G. (2001) Boursin, a sea urchin bimC kinesin protein, plays a role in anaphase and cytokinesis. *J Cell Sci* **114**, 481-491
189. Aury, J. M., Jaillon, O., Duret, L., Noel, B., Jubin, C., Porcel, B. M., Segurens, B., Daubin, V., Anthouard, V., Aiach, N., Arnaiz, O., Billaut, A., Beisson, J., Blanc, I., Bouhouche, K., Camara, F., Duharcourt, S., Guigo, R., Gogendeau, D., Katinka, M., Keller, A. M., Kissmehl, R., Klotz, C., Koll, F., Le Mouel, A., Lepere, G., Malinsky, S., Nowacki, M., Nowak, J. K., Plattner, H., Poulain, J., Ruiz, F., Serrano, V., Zagulski, M., Dessen, P., Betermier, M., Weissenbach, J., Scarpelli, C., Schachter, V., Sperling, L., Meyer, E., Cohen, J., and Wincker, P. (2006) Global trends of whole-genome duplications revealed by the ciliate *Paramecium tetraurelia*. *Nature* **444**, 171-178
190. Hane, J. K., Lowe, R. G., Solomon, P. S., Tan, K. C., Schoch, C. L., Spatafora, J. W., Crous, P. W., Kodira, C., Birren, B. W., Galagan, J. E., Torriani, S. F., McDonald, B. A., and Oliver, R. P. (2007) Dothideomycete plant interactions illuminated by genome sequencing and EST analysis of the wheat pathogen *Stagonospora nodorum*. *Plant Cell* **19**, 3347-3368
191. Jeffries, T. W., Grigoriev, I. V., Grimwood, J., Laplaza, J. M., Aerts, A., Salamov, A., Schmutz, J., Lindquist, E., Dehal, P., Shapiro, H., Jin, Y. S., Passoth, V., and Richardson, P. M. (2007) Genome sequence of the lignocellulose-bioconverting and xylose-fermenting yeast *Pichia stipitis*. *Nat Biotechnol* **25**, 319-326
192. Hall, N., Pain, A., Berriman, M., Churcher, C., Harris, B., Harris, D., Mungall, K., Bowman, S., Atkin, R., Baker, S., Barron, A., Brooks, K., Buckee, C. O., Burrows, C., Cherevach, I., Chillingworth, C., Chillingworth, T., Christodoulou, Z., Clark, L., Clark, R., Corton, C., Cronin, A., Davies, R., Davis, P., Dear, P., Dearden, F., Doggett, J., Feltwell, T., Goble, A., Goodhead, I., Gwilliam, R., Hamlin, N., Hance, Z., Harper, D., Hauser, H., Hornsby, T., Holroyd, S., Horrocks, P., Humphray, S., Jagels, K., James, K. D., Johnson, D., Kerhornou, A., Knights, A., Konfortov, B., Kyes, S., Larke, N., Lawson, D., Lennard, N., Line, A., Maddison, M., McLean, J., Mooney, P., Moule, S., Murphy, L., Oliver, K., Ormond, D., Price, C., Quail, M. A., Rabbinowitsch, E., Rajandream, M. A., Rutter, S., Rutherford, K. M., Sanders, M., Simmonds, M., Seeger, K., Sharp, S., Smith, R., Squares, R., Squares, S., Stevens, K., Taylor, K., Tivey, A., Unwin, L., Whitehead, S., Woodward, J., Sulston, J. E., Craig, A., Newbold, C., and Barrell, B. G. (2002) Sequence of *Plasmodium falciparum* chromosomes 1, 3-9 and 13. *Nature* **419**, 527-531
193. Carlton, J. M., Adams, J. H., Silva, J. C., Bidwell, S. L., Lorenzi, H., Caler, E., Crabtree, J., Angiuoli, S. V., Merino, E. F., Amedeo, P., Cheng, Q., Coulson, R. M., Crabb, B. S., Del Portillo, H. A., Essien, K., Feldblyum, T. V., Fernandez-Becerra, C., Gilson, P. R., Gueye, A. H., Guo, X., Kang'a, S., Kooij, T. W., Korsinczky, M., Meyer, E. V., Nene, V., Paulsen, I., White, O., Ralph, S. A., Ren, Q., Sargeant, T. J., Salzberg, S. L., Stoeckert, C. J., Sullivan, S. A., Yamamoto, M. M., Hoffman, S. L., Wortman, J. R., Gardner, M. J., Galinski, M. R., Barnwell, J. W., and Fraser-Liggett, C. M. (2008) Comparative genomics of the neglected human malaria parasite *Plasmodium vivax*. *Nature* **455**, 757-763
194. Bloecker, H., Boecher, M., Brandt, P., Mewes, H. W., Weil, B., Amid, C., Osanger, A., Fobo, G., Han, M., and Wiemann, S. (2004) The German cDNA Consortium. MIPS, Neuherberg. Direct submission to EMBL/GenBank/DDBJ databases
195. Stout, J. R., Rizk, R. S., Kline, S. L., and Walczak, C. E. (2006) Deciphering protein function during mitosis in PtK cells using RNAi. *BMC Cell Biol* **7**, 26
196. Muresan, V., Abramson, T., Lyass, A., Winter, D., Porro, E., Hong, F., Chamberlin, N. L., and Schnapp, B. J. (1998) KIF3C and KIF3A form a novel neuronal heteromeric kinesin that associates with membrane vesicles. *Mol Biol Cell* **9**, 637-652

197. Rogers, K. R., Griffin, M., and Brophy, P. J. (1998) The secretory epithelial cells of the choroid plexus employ a novel kinesin-related protein. *Brain Res Mol Brain Res* **55**, 355
198. Sperry, A. O., and Zhao, L. P. (1996) Kinesin-related proteins in the mammalian testes: candidate motors for meiosis and morphogenesis. *Mol Biol Cell* **7**, 289-305
199. Gibbs, R. A., Weinstock, G. M., Metzker, M. L., Muzny, D. M., Sodergren, E. J., Scherer, S., Scott, G., Steffen, D., Worley, K. C., Burch, P. E., Okwuonu, G., Hines, S., Lewis, L., DeRamo, C., Delgado, O., Dugan-Rocha, S., Miner, G., Morgan, M., Hawes, A., Gill, R., Celera, Holt, R. A., Adams, M. D., Amanatides, P. G., Baden-Tillson, H., Barnstead, M., Chin, S., Evans, C. A., Ferriera, S., Fosler, C., Glodek, A., Gu, Z., Jennings, D., Kraft, C. L., Nguyen, T., Pfannkoch, C. M., Sitter, C., Sutton, G. G., Venter, J. C., Woodage, T., Smith, D., Lee, H. M., Gustafson, E., Cahill, P., Kana, A., Doucette-Stamm, L., Weinstock, K., Fechtel, K., Weiss, R. B., Dunn, D. M., Green, E. D., Blakesley, R. W., Bouffard, G. G., De Jong, P. J., Osoegawa, K., Zhu, B., Marra, M., Schein, J., Bosdet, I., Fjell, C., Jones, S., Krzywinski, M., Mathewson, C., Siddiqui, A., Wye, N., McPherson, J., Zhao, S., Fraser, C. M., Shetty, J., Shatsman, S., Geer, K., Chen, Y., Abramzon, S., Nierman, W. C., Havlak, P. H., Chen, R., Durbin, K. J., Egan, A., Ren, Y., Song, X. Z., Li, B., Liu, Y., Qin, X., Cawley, S., Cooney, A. J., D'Souza, L. M., Martin, K., Wu, J. Q., Gonzalez-Garay, M. L., Jackson, A. R., Kalafus, K. J., McLeod, M. P., Milosavljevic, A., Virk, D., Volkov, A., Wheeler, D. A., Zhang, Z., Bailey, J. A., Eichler, E. E., Tuzun, E., Birney, E., Mongin, E., Ureta-Vidal, A., Woodwark, C., Zdobnov, E., Bork, P., Suyama, M., Torrents, D., Alexandersson, M., Trask, B. J., Young, J. M., Huang, H., Wang, H., Xing, H., Daniels, S., Gietzen, D., Schmidt, J., Stevens, K., Vitt, U., Wingrove, J., Camara, F., Mar Alba, M., Abril, J. F., Guigo, R., Smit, A., Dubchak, I., Rubin, E. M., Couronne, O., Poliakov, A., Hubner, N., Ganten, D., Goesele, C., Hummel, O., Kreitler, T., Lee, Y. A., Monti, J., Schulz, H., Zimdahl, H., Himmelbauer, H., Lehrach, H., Jacob, H. J., Bromberg, S., Gullings-Handley, J., Jensen-Seaman, M. I., Kwitek, A. E., Lazar, J., Pasko, D., Tonellato, P. J., Twigger, S., Ponting, C. P., Duarte, J. M., Rice, S., Goodstadt, L., Beatson, S. A., Emes, R. D., Winter, E. E., Webber, C., Brandt, P., Nyakatura, G., Adetobi, M., Chiaromonte, F., Elnitski, L., Eswara, P., Hardison, R. C., Hou, M., Kolbe, D., Makova, K., Miller, W., Nekrutenko, A., Riemer, C., Schwartz, S., Taylor, J., Yang, S., Zhang, Y., Lindpaintner, K., Andrews, T. D., Caccamo, M., Clamp, M., Clarke, L., Curwen, V., Durbin, R., Eyras, E., Searle, S. M., Cooper, G. M., Batzoglou, S., Brudno, M., Sidow, A., Stone, E. A., Payseur, B. A., Bourque, G., Lopez-Otin, C., Puente, X. S., Chakrabarti, K., Chatterji, S., Dewey, C., Pachter, L., Bray, N., Yap, V. B., Caspi, A., Tesler, G., Pevzner, P. A., Haussler, D., Roskin, K. M., Baertsch, R., Clawson, H., Furey, T. S., Hinrichs, A. S., Karolchik, D., Kent, W. J., Rosenbloom, K. R., Trumbower, H., Weirauch, M., Cooper, D. N., Stenson, P. D., Ma, B., Brent, M., Arumugam, M., Shteynberg, D., Copley, R. R., Taylor, M. S., Riethman, H., Mudunuri, U., Peterson, J., Guyer, M., Felsenfeld, A., Old, S., Mockrin, S., and Collins, F. (2004) Genome sequence of the Brown Norway rat yields insights into mammalian evolution. *Nature* **428**, 493-521
200. Faire, K., Gruber, D., and Bulinski, J. C. (1998) Identification of kinesin-like molecules in myogenic cells. *Eur J Cell Biol* **77**, 27-34
201. Junco, A., Bhullar, B., Tarnasky, H. A., and van der Hoorn, F. A. (2001) Kinesin light-chain KLC3 expression in testis is restricted to spermatids. *Biol Reprod* **64**, 1320-1330
202. Roof, D. M., Meluh, P. B., and Rose, M. D. (1992) Kinesin-related proteins required for assembly of the mitotic spindle. *J Cell Biol* **118**, 95-108
203. Lillie, S. H., and Brown, S. S. (1992) Suppression of a myosin defect by a kinesin-related gene. *Nature* **356**, 358-361
204. Rieger, M., Bruckner, M., Schafer, M., and Muller-Auer, S. (1997) Sequence analysis of 203 kilobases from *Saccharomyces cerevisiae* chromosome VII. *Yeast* **13**, 1077-1090
205. Lukacs, M. F., Harstad, H., Grimholt, U., Beetz-Sargent, M., Cooper, G. A., Reid, L., Bakke, H. G., Phillips, R. B., Miller, K. M., Davidson, W. S., and Koop, B. F. (2007) Genomic organization

of duplicated major histocompatibility complex class I regions in Atlantic salmon (*Salmo salar*). *BMC Genomics* **8**, 251
206. Hagan, I., and Yanagida, M. (1990) Novel potential mitotic motor protein encoded by the fission yeast cut7+ gene. *Nature* **347**, 563-566
207. Garcia, M. A., Koonrugsa, N., and Toda, T. (2002) Two kinesin-like Kin I family proteins in fission yeast regulate the establishment of metaphase and the onset of anaphase A. *Curr Biol* **12**, 610-621
208. Brazer, S. C., Williams, H. P., Chappell, T. G., and Cande, W. Z. (2000) A fission yeast kinesin affects Golgi membrane recycling. *Yeast* **16**, 149-166
209. Wood, V., Gwilliam, R., Rajandream, M. A., Lyne, M., Lyne, R., Stewart, A., Sgouros, J., Peat, N., Hayles, J., Baker, S., Basham, D., Bowman, S., Brooks, K., Brown, D., Brown, S., Chillingworth, T., Churcher, C., Collins, M., Connor, R., Cronin, A., Davis, P., Feltwell, T., Fraser, A., Gentles, S., Goble, A., Hamlin, N., Harris, D., Hidalgo, J., Hodgson, G., Holroyd, S., Hornsby, T., Howarth, S., Huckle, E. J., Hunt, S., Jagels, K., James, K., Jones, L., Jones, M., Leather, S., McDonald, S., McLean, J., Mooney, P., Moule, S., Mungall, K., Murphy, L., Niblett, D., Odell, C., Oliver, K., O'Neil, S., Pearson, D., Quail, M. A., Rabbinowitsch, E., Rutherford, K., Rutter, S., Saunders, D., Seeger, K., Sharp, S., Skelton, J., Simmonds, M., Squares, R., Squares, S., Stevens, K., Taylor, K., Taylor, R. G., Tivey, A., Walsh, S., Warren, T., Whitehead, S., Woodward, J., Volckaert, G., Aert, R., Robben, J., Grymonprez, B., Weltjens, I., Vanstreels, E., Rieger, M., Schafer, M., Muller-Auer, S., Gabel, C., Fuchs, M., Dusterhoft, A., Fritzc, C., Holzer, E., Moestl, D., Hilbert, H., Borzym, K., Langer, I., Beck, A., Lehrach, H., Reinhardt, R., Pohl, T. M., Eger, P., Zimmermann, W., Wedler, H., Wambutt, R., Purnelle, B., Goffeau, A., Cadieu, E., Dreano, S., Gloux, S., Lelaure, V., Mottier, S., Galibert, F., Aves, S. J., Xiang, Z., Hunt, C., Moore, K., Hurst, S. M., Lucas, M., Rochet, M., Gaillardin, C., Tallada, V. A., Garzon, A., Thode, G., Daga, R. R., Cruzado, L., Jimenez, J., Sanchez, M., del Rey, F., Benito, J., Dominguez, A., Revuelta, J. L., Moreno, S., Armstrong, J., Forsburg, S. L., Cerutti, L., Lowe, T., McCombie, W. R., Paulsen, I., Potashkin, J., Shpakovski, G. V., Ussery, D., Barrell, B. G., and Nurse, P. (2002) The genome sequence of *Schizosaccharomyces pombe*. *Nature* **415**, 871-880
210. Browning, H., Hayles, J., Mata, J., Aveline, L., Nurse, P., and McIntosh, J. R. (2000) Tea2p is a kinesin-like protein required to generate polarized growth in fission yeast. *J Cell Biol* **151**, 15-28
211. Reddy, A. S., Narasimhulu, S. B., Safadi, F., and Golovkin, M. (1996) A plant kinesin heavy chain-like protein is a calmodulin-binding protein. *Plant J* **10**, 9-21
212. Wright, B. D., Henson, J. H., Wedaman, K. P., Willy, P. J., Morand, J. N., and Scholey, J. M. (1991) Subcellular localization and sequence of sea urchin kinesin heavy chain: evidence for its association with membranes in the mitotic apparatus and interphase cytoplasm. *J Cell Biol* **113**, 817-833
213. Cole, D. G., Chinn, S. W., Wedaman, K. P., Hall, K., Vuong, T., and Scholey, J. M. (1993) Novel heterotrimeric kinesin-related protein purified from sea urchin eggs. *Nature* **366**, 268-270
214. Rogers, G. C., Chui, K. K., Lee, E. W., Wedaman, K. P., Sharp, D. J., Holland, G., Morris, R. L., and Scholey, J. M. (2000) A kinesin-related protein, KRP(180), positions prometaphase spindle poles during early sea urchin embryonic cell division. *J Cell Biol* **150**, 499-512
215. Grummt, M., Pistor, S., Lottspeich, F., and Schliwa, M. (1998) Cloning and functional expression of a 'fast' fungal kinesin. *FEBS Lett* **427**, 79-84
216. Brown, J. M., Marsala, C., Kosoy, R., and Gaertig, J. (1999) Kinesin-II is preferentially targeted to assembling cilia and is required for ciliogenesis and normal cytokinesis in Tetrahymena. *Mol Biol Cell* **10**, 3081-3096
217. Eisen, J. A., Coyne, R. S., Wu, M., Wu, D., Thiagarajan, M., Wortman, J. R., Badger, J. H., Ren, Q., Amedeo, P., Jones, K. M., Tallon, L. J., Delcher, A. L., Salzberg, S. L., Silva, J. C., Haas, B. J., Majoros, W. H., Farzad, M., Carlton, J. M., Smith, R. K., Jr., Garg, J., Pearlman, R. E., Karrer, K. M., Sun, L., Manning, G., Elde, N. C., Turkewitz, A. P., Asai, D. J., Wilkes, D. E., Wang, Y., Cai, H., Collins, K., Stewart, B. A., Lee, S. R., Wilamowska, K., Weinberg, Z., Ruzzo, W. L.,

Wloga, D., Gaertig, J., Frankel, J., Tsao, C. C., Gorovsky, M. A., Keeling, P. J., Waller, R. F., Patron, N. J., Cherry, J. M., Stover, N. A., Krieger, C. J., del Toro, C., Ryder, H. F., Williamson, S. C., Barbeau, R. A., Hamilton, E. P., and Orias, E. (2006) Macronuclear genome sequence of the ciliate *Tetrahymena thermophila*, a model eukaryote. *PLoS Biol* **4**, e286

218. Carlton, J. M., Hirt, R. P., Silva, J. C., Delcher, A. L., Schatz, M., Zhao, Q., Wortman, J. R., Bidwell, S. L., Alsmark, U. C., Besteiro, S., Sicheritz-Ponten, T., Noel, C. J., Dacks, J. B., Foster, P. G., Simillion, C., Van de Peer, Y., Miranda-Saavedra, D., Barton, G. J., Westrop, G. D., Muller, S., Dessi, D., Fiori, P. L., Ren, Q., Paulsen, I., Zhang, H., Bastida-Corcuera, F. D., Simoes-Barbosa, A., Brown, M. T., Hayes, R. D., Mukherjee, M., Okumura, C. Y., Schneider, R., Smith, A. J., Vanacova, S., Villalvazo, M., Haas, B. J., Pertea, M., Feldblyum, T. V., Utterback, T. R., Shu, C. L., Osoegawa, K., de Jong, P. J., Hrdy, I., Horvathova, L., Zubacova, Z., Dolezal, P., Malik, S. B., Logsdon, J. M., Jr., Henze, K., Gupta, A., Wang, C. C., Dunne, R. L., Upcroft, J. A., Upcroft, P., White, O., Salzberg, S. L., Tang, P., Chiu, C. H., Lee, Y. S., Embley, T. M., Coombs, G. H., Mottram, J. C., Tachezy, J., Fraser-Liggett, C. M., and Johnson, P. J. (2007) Draft genome sequence of the sexually transmitted pathogen *Trichomonas vaginalis*. *Science* **315**, 207-212

219. Dutoya, S., Gibert, S., Lemercier, G., Santarelli, X., Baltz, D., Baltz, T., and Bakalara, N. (2001) A novel C-terminal kinesin is essential for maintaining functional acidocalcisomes in *Trypanosoma brucei*. *J Biol Chem* **276**, 49117-49124

220. Lehmler, C., Steinberg, G., Snetselaar, K. M., Schliwa, M., Kahmann, R., and Bolker, M. (1997) Identification of a motor protein required for filamentous growth in *Ustilago maydis*. *EMBO J* **16**, 3464-3473

221. Velasco, R., Zharkikh, A., Troggio, M., Cartwright, D. A., Cestaro, A., Pruss, D., Pindo, M., Fitzgerald, L. M., Vezzulli, S., Reid, J., Malacarne, G., Iliev, D., Coppola, G., Wardell, B., Micheletti, D., Macalma, T., Facci, M., Mitchell, J. T., Perazzolli, M., Eldredge, G., Gatto, P., Oyzerski, R., Moretto, M., Gutin, N., Stefanini, M., Chen, Y., Segala, C., Davenport, C., Dematte, L., Mraz, A., Battilana, J., Stormo, K., Costa, F., Tao, Q., Si-Ammour, A., Harkins, T., Lackey, A., Perbost, C., Taillon, B., Stella, A., Solovyev, V., Fawcett, J. A., Sterck, L., Vandepoele, K., Grando, S. M., Toppo, S., Moser, C., Lanchbury, J., Bogden, R., Skolnick, M., Sgaramella, V., Bhatnagar, S. K., Fontana, P., Gutin, A., Van de Peer, Y., Salamini, F., and Viola, R. (2007) A high quality draft consensus sequence of the genome of a heterozygous grapevine variety. *PLoS One* **2**, e1326

222. Le Guellec, R., Paris, J., Couturier, A., Roghi, C., and Philippe, M. (1991) Cloning by differential screening of a *Xenopus* cDNA that encodes a kinesin-related protein. *Mol Cell Biol* **11**, 3395-3398

223. Walczak, C. E., Verma, S., and Mitchison, T. J. (1997) XCTK2: a kinesin-related protein that promotes mitotic spindle assembly in *Xenopus laevis* egg extracts. *J Cell Biol* **136**, 859-870

224. Vernos, I., Heasman, J., and Wylie, C. (1993) Multiple kinesin-like transcripts in *Xenopus* oocytes. *Dev Biol* **157**, 232-239

225. De Marco, V., Burkhard, P., Le Bot, N., Vernos, I., and Hoenger, A. (2001) Analysis of heterodimer formation by Xklp3A/B, a newly cloned kinesin-II from *Xenopus laevis*. *EMBO J* **20**, 3370-3379

226. Walczak, C. E., Mitchison, T. J., and Desai, A. (1996) XKCM1: a *Xenopus* kinesin-related protein that regulates microtubule dynamics during mitotic spindle assembly. *Cell* **84**, 37-47

227. Westerholm-Parvinen, A., Vernos, I., and Serrano, L. (2000) Kinesin subfamily UNC104 contains a FHA domain: boundaries and physicochemical characterization. *FEBS Lett* **486**, 285-290

228. Vernos, I., Raats, J., Hirano, T., Heasman, J., Karsenti, E., and Wylie, C. (1995) Xklp1, a chromosomal *Xenopus* kinesin-like protein essential for spindle organization and chromosome positioning. *Cell* **81**, 117-127

229. Klein, S. L., Strausberg, R. L., Wagner, L., Pontius, J., Clifton, S. W., and Richardson, P. (2002) Genetic and genomic tools for *Xenopus* research: The NIH *Xenopus* initiative. *Dev Dyn* **225**, 384-391
230. Boleti, H., Karsenti, E., and Vernos, I. (1996) Xklp2, a novel *Xenopus* centrosomal kinesin-like protein required for centrosome separation during mitosis. *Cell* **84**, 49-59
231. Funabiki, H., and Murray, A. W. (2000) The *Xenopus* chromokinesin Xkid is essential for metaphase chromosome alignment and must be degraded to allow anaphase chromosome movement. *Cell* **102**, 411-424
232. Messitt, T. J., Gagnon, J. A., Kreiling, J. A., Pratt, C. A., Yoon, Y. J., and Mowry, K. L. (2008) Multiple kinesin motors coordinate cytoplasmic RNA transport on a subpopulation of microtubules in *Xenopus* oocytes. *Dev Cell* **15**, 426-436
233. Kim, Y., Heuser, J. E., Waterman, C. M., and Cleveland, D. W. (2008) CENP-E combines a slow, processive motor and a flexible coiled coil to produce an essential motile kinetochore tether. *J Cell Biol* **181**, 411-419
234. Antonio, C., Ferby, I., Wilhelm, H., Jones, M., Karsenti, E., Nebreda, A. R., and Vernos, I. (2000) Xkid, a chromokinesin required for chromosome alignment on the metaphase plate. *Cell* **102**, 425-435
