# Supplementary figures and images for: Allostery Wiring Map for Kinesin Energy Transduction and Its Evolution
Source: J Biol Chem. 2016 Aug 8;291(40):20932–45. doi: 10.1074/jbc.M116.733675 (PMC5076506; doi:10.1074/jbc.M116.733675)

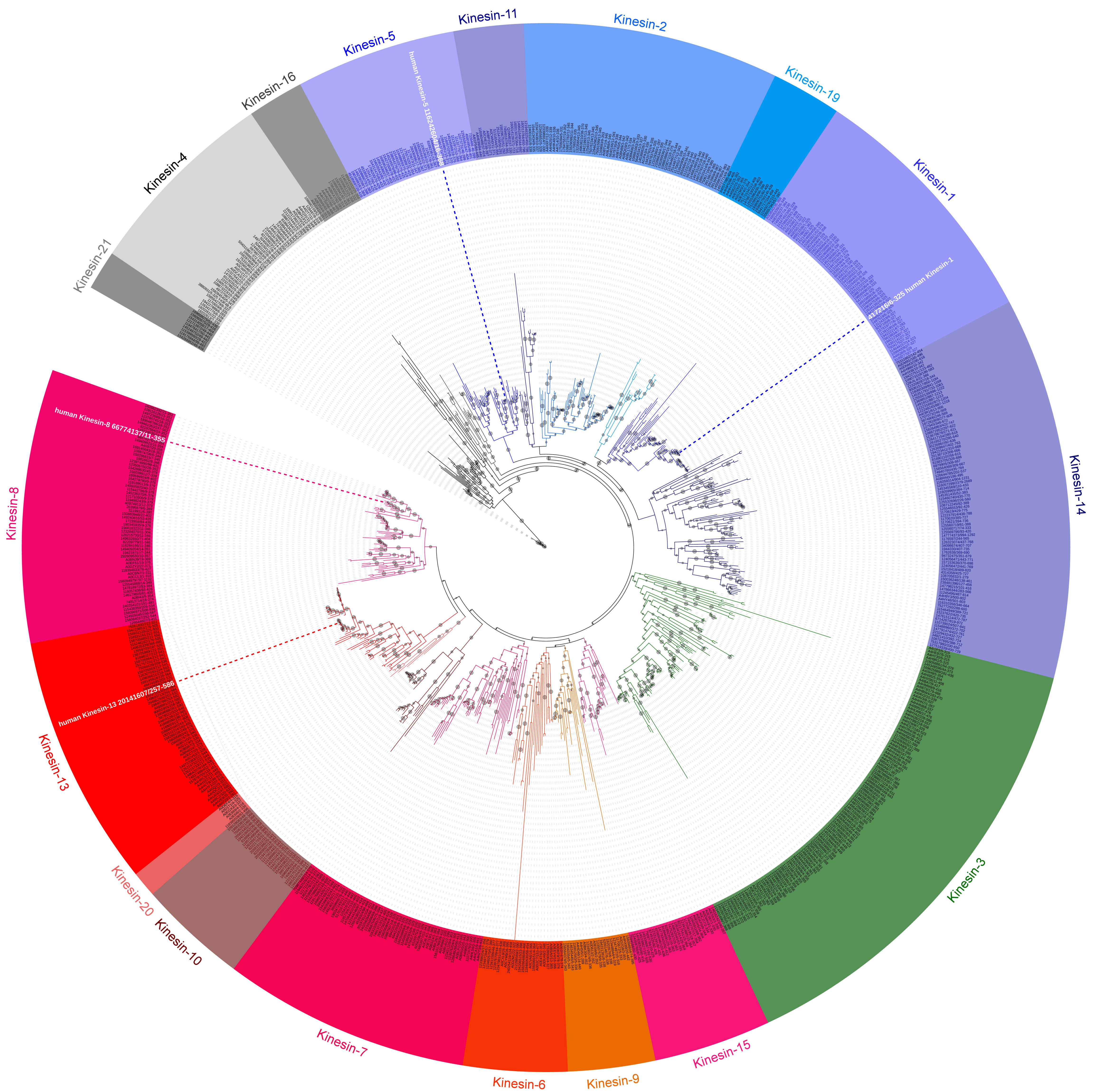

Supplement: Supplemental Data [file supp_M116.733675_SI_File4.pdf]
